# Bistelluridopnictanes as gateways to telluradipnictiranes and a cyclic [Bi_2_Te_2_]^2+^ dication

**DOI:** 10.1039/d6sc02907h

**Published:** 2026-06-29

**Authors:** Charlotte E. R. van Halteren, Christoph Wölper, Leon Kapp, Stephan Schulz

**Affiliations:** a Institute of Inorganic Chemistry, University of Duisburg-Essen Universitätsstraße 5-7 45141 Essen Germany stephan.schulz@uni-due.de; b Center for Nanointegration Duisburg-Essen (CENIDE), University of Duisburg-Essen Carl-Benz-Straße 199 47057 Duisburg Germany

## Abstract

The controlled assembly of molecular pnictogen–tellurium (Pn–Te) frameworks remains a longstanding synthetic challenge due to the intrinsic lability of Pn–Te bonds, particularly of the heaviest pnictogens, resulting in a scarcity of well-defined compounds. Here we present a general synthesis strategy that enables reliable access to a homologous series of bistelluridopnictanes DMPPn(TePh)_2_ (Pn = As (1), Sb (2), Bi (3); DMP = 2,6-Mes_2_C_6_H_3_, Mes = 2,4,6-Me_3_C_6_H_2_), providing a systematic platform for comparing structure, bonding, and reactivity across the heavier pnictogens. Comprehensive spectroscopic (^1^H, ^13^C, ^125^Te NMR, IR) and crystallographic studies (sc-XRD) reveal clear periodic trends in Pn–Te interactions and coordination geometries. Decomposition experiments uncover a unified initial reductive elimination of diphenylditelluride (Ph_2_Te_2_), followed by consecutive pnictogen-dependent follow-up reactions including the formation of dipnictenes and three-membered telluradipnictiranes, respectively. Our studies finally resulted in the isolation of the first telluradiarsirane DMPAs(µ-Te)AsDMP (4) and very rare examples of the corresponding Sb and Bi congeners DMPPn(µ-Te)PnDMP (Pn = Sb (5), Bi (6)). The attempted synthesis of cationic species by reaction of compounds 1–3 with silver triflate yielded [DMPBi(TePh)]_2_[SbF_6_]_2_ (7) containing an unprecedented dicationic Bi_2_Te_2_ four-membered heterocycle, representing the first mixed Bi–Te analogue of a structural motif previously restricted to lighter and homonuclear pnictogen systems. Our findings expand the accessible chemical space of heavy pnictogen–tellurium compounds, illuminate fundamental trends in bonding and reactivity, and provide a modular entry point toward tailored molecular precursors for chalcogenide materials and advanced main-group architectures.

## Abstract 2

Bistelluridopnictanes are promising reagents for the controlled synthesis of a variety of cyclic Pn–Te structure motifs, including rare telluradipnictiranes and a cyclic [Bi_2_Te_2_]^2+^ dication.

## Graphical abstract

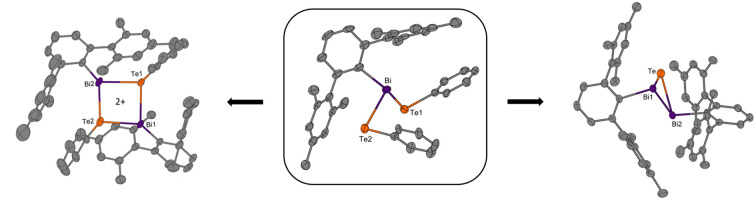

## Introduction

The rational design of molecular compounds featuring covalent bonds between group 15 (pnictogen) and group 16 (chalcogen) elements, particularly Sb, Bi, and Te, has emerged as a versatile platform at the interface of synthetic inorganic chemistry and functional materials science.^1^ Pnictogen–chalcogen (Pn–Ch, Pn = As–Bi; Ch = S–Te) bonded systems combine diverse oxidation states,^2,3^ stereochemically active lone pairs,^4,5^ and tunable electronic properties,^6^ enabling fundamental studies of bonding,^3^ structure–reactivity relationships,^3,7^ and solid-state phenomena such as thermochromism.^8^ Moreover, well-defined Pn–Ch compounds can serve as tailored molecular precursors^9^ for metal chalcogenide materials, which are of interest in photovoltaics,^10^ thermoelectrics,^11^ catalysis,^12^ and topological insulators,^13^ respectively.

**Fig. 1:**
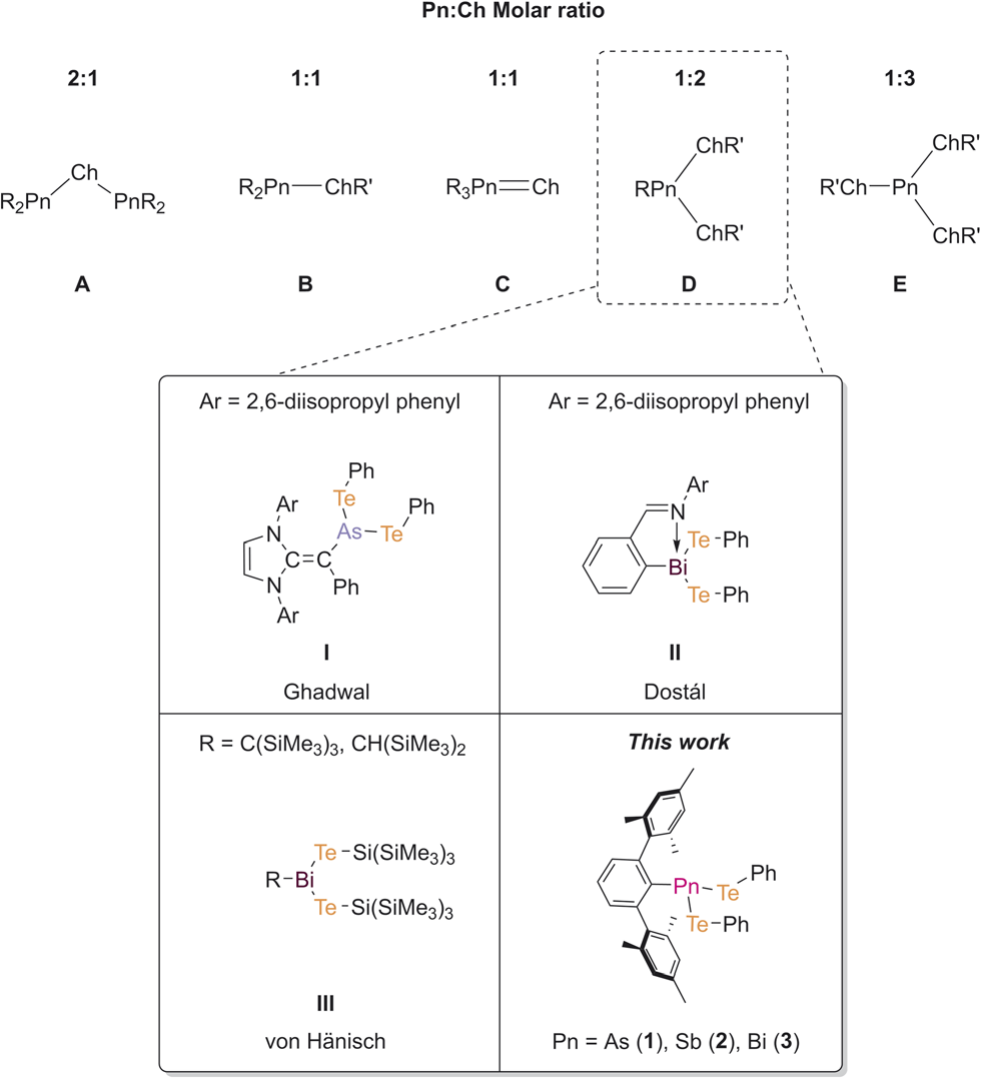
Structurally characterised compounds of the general form RPn(TeR′)_2_ (Pn = As–Bi; R, R′ = organic or organosilicon substituents).^14,17,18^

Despite their high potential for technical applications, molecular compounds with multiple Pn–Te bonds, namely compounds of the types Pn(TeR)_3_ and R′Pn(TeR)_2_ (R, R′ = organic ligand), respectively, are extremely rare. Synthetic access to well-defined organometallic Pn–Ch compounds is fundamentally limited by the intrinsic weakness of the Pn–Ch bonds and their pronounced sensitivity toward hydrolysis.^14^ While several polyionic or cluster-type species^15^ with multiple Pn-centred Te bonds have been reported, neutral molecules remain scarce. For example, (*t*Bu_2_PhSiTe)_3_Bi, which was synthesised by von Hänisch *et al.* by dechlorosilylation reaction of BiCl_3_ with *t*Bu_2_PhSiTeSiMe_3_, represents the only structurally characterised example of the general form Pn(TeR′)_3_ (Pn = As–Bi; R′ = organic substituent).^16^ Reducing the number of Pn–Te bonds to two gives rise to compounds of the general type RPn(TeR′)_2_ (Pn = As–Bi; R, R′ = organic substituent), which are slightly more developed. While {(IPr)C(Ph)}As(TePh)_2_ (IPr = C(CHNDipp)_2_, Dipp = 2,6-(*i*Pr)_2_C_6_H_3_, Fig. 1, I),^17^ which was synthesised by Ghadwal *et al.* through As–As bond cleavage reaction of the corresponding diarsene with diphenylditelluride, is the only arsenic derivative of the desired type, the number of corresponding antimony compounds is larger. They have been typically synthesised by reactions of diorganoditelluranes R′TeTeR′ (R′ = Ph, Me, *p*-Tol) with oligomeric low-valent organoantimony species (RSb)_*x*_ (R = *o*-CHNDipp-C_6_H_4_, Et, Mes, CH_2_SiMe_3_),^18,19^ in which the Sb atoms adopt the formal oxidation state +I. However, these molecules are typically obtained in rather low yields and their characterisation remains incomplete, with no crystallographic data and insufficient NMR analysis. For bismuth, only three structurally characterised examples have been reported to the best of our knowledge. Dostál *et al.* reported on the synthesis of *o*-CHNDippC_6_H_4_Bi(TePh)_2_ (Fig. 1, II), which was obtained by reaction of diphenylditelluride with the *in situ* generated bismuthinindene.^18^ Furthermore, von Hänisch *et al.* synthesised RBi(TeSi(SiMe_3_)_3_)_2_ (R = C(SiMe_3_)_3_ and CH(SiMe_3_)_2_) (Fig. 1, III) *via* dechlorosilylation reaction of RBiCl_2_ with two equivalents of (Me_3_Si)_2_SiTeSiMe_3_.^14^ All of these compounds suffer from limited stability in solution and were only obtained in poor yields.

Collectively, the scarcity of neutral molecular species bearing multiple Pn-centred Pn–Te bonds, combined with the limited structural and reactivity data available, underscores the need for a systematic exploration of this compound class. Motivated by these gaps, we sought to access homologous derivatives featuring two Pn–Te bonds (Pn = As–Bi) to enable reliable structural comparison and to provide a basis for evaluating their reactivity.

We herein report the synthesis, full characterisation and initial reactivity studies of a series of bistelluridopnictanes DMPAs(TePh)_2_ (1), DMPSb(TePh)_2_ (2), and DMPBi(TePh)_2_ (3) (DMP = 2,6-Mes_2_C_6_H_3_, Mes = 2,4,6-Me_3_C_6_H_2_, Fig. 1), respectively. These compounds provide a direct comparison, enabling insight into structural trends, bond metrics, and decomposition pathways. Moreover, they reveal unique reactivity patterns and were found to serve as valuable starting compounds to heterocyclic compounds, including rare three-membered telluradipnictiranes DMPPn(µ-Te)PnDMP as well as a four-memebered Bi_2_Te_2_ dication, respectively.

## Results and discussion

### Synthesis of bistelluridopnictanes 1–3

**Scheme 1:**
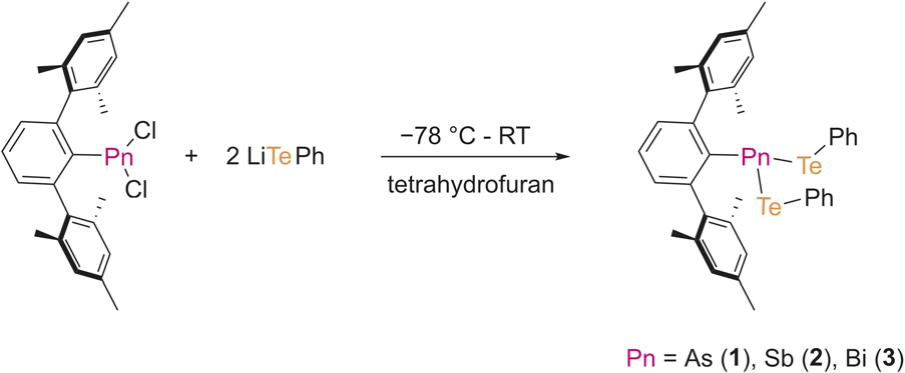
Synthesis of the compounds 1–3.

In principle, molecular pnictogen–tellurium compounds bearing two Pn–Te bonds can be accessed through oxidative addition reactions of ditellurides with low-valent pnictogen precursors. Although substantial progress has been made in the synthesis and isolation of low-valent heavy Pn(i) compounds,^20^ including both Lewis base-stabilised^21^ and Lewis base-free derivatives,^22^ their application as conceptual building blocks remains comparatively less explored, partly due to their limited stability that can impede controlled synthetic transformations. To overcome these limitations and to establish a more flexible and broadly applicable synthetic route toward the targeted molecular frameworks RPn(TeR′)_2_, we opted for an alternative strategy based on salt elimination reaction, which bypasses the need for Pn(i) precursors and enables the controlled construction of Pn–Te bonds under rather mild conditions. To achieve kinetic stabilisation of the targeted bonding motif, we employed the sterically demanding DMP substituent, which has previously proven effective in stabilising highly reactive main-group species.^23^ Treatment of the corresponding dichloropnictanes DMPPnCl_2_ (Pn = As–Bi) with two equivalents of *in situ* generated LiTePh at −78 °C in tetrahydrofuran (THF) afforded solutions of the new compounds DMPAs(TePh)_2_ (1), DMPSb(TePh)_2_ (2), and DMPBi(TePh)_2_ (3) (see Scheme 1). Following warming to room temperature, removal of THF, and extraction of the crude material with *n*-hexane, the desired products 1–3 were isolated as orange (1 and 2) or red crystals (3) in yields of 46–48%.

### Spectroscopic characterisation of 1–3

Compounds 1–3 were characterised by multinuclear NMR spectroscopy (see SI, Section I). In all cases, the ^1^H and ^13^C NMR spectra display the expected sets of resonances corresponding to the aromatic protons and carbon nuclei of both the DMP and TePh substituents, along with the two singlets for the magnetically equivalent methyl groups of the DMP ligand. ^125^Te NMR spectra show one resonance for each compound, appearing at 620.2 ppm for 1, 311.8 ppm for 2, and 323.3 ppm for 3. The evidently more downfield shifted ^125^Te resonance observed for 1 is consistent with the higher electronegativity of As (2.18) relative to Sb (2.05) and Bi (2.02).^24^ The increased electron-withdrawing character of As results in a more pronounced deshielding of the tellurium nuclei. In contrast, the similar electronegativities of Sb and Bi are reflected in the comparable ^125^Te chemical shifts of compounds 2 and 3, which display nearly identical resonance frequencies. The ^125^Te NMR chemical shift of the arsenic compound 1 is fully consistent with the literature value of 619 ppm reported for {(IPr)C(Ph)}As(TePh)_2_ (I),^17^ which features the same –As(TePh)_2_ structural motif. In contrast, the ^125^Te resonance of the bismuth analogue 3 deviates markedly from those of the related complexes RBi(TeSi(SiMe_3_)_3_)_2_ (R = C(SiMe_3_)_3_ or CH(SiMe_3_)_2_, III),^14^ each containing a –Bi(TeR)_2_ fragment. These reference compounds exhibit substantially upfield-shifted resonances at −675.7 and −761.7 ppm, respectively, reflecting the significantly enhanced electron density at the tellurium centres imparted by the more electron donating silyl substituents, in marked contrast to phenyl groups present in 3. In comparison to the antimony compound 2, structurally related tellurium–antimony species exhibit upfield-shifted ^125^Te NMR resonances, *i.e.*, Me_2_SbTeTol (137.2 ppm) and Me_2_SbTeMe (−271 ppm), respectively.^25^

### Single crystal X-ray structures of 1–3

**Fig. 2:**
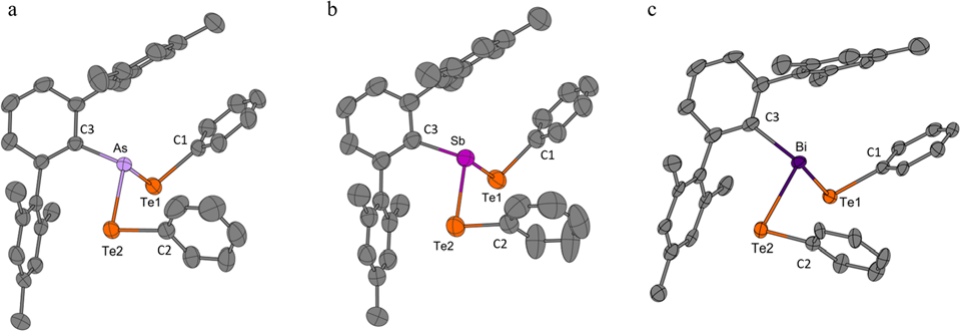
Solid-state molecular structures of (a) DMPAs(TePh)_2_ (1), (b) DMPSb(TePh)_2_ (2), and (c) DMPBi(TePh)_2_ (3). H atoms and alternate positions have been omitted for clarity and displacement ellipsoids are drawn at the 50% probability level.

Single crystals of 1–3 were obtained by storing *n*-hexane solutions of the respective compounds at −30 °C for 24 hours. Interestingly, compounds 1 and 2 undergo a distinct thermochromic phase transition, exhibiting a reversible colour change from orange to yellow upon cooling below 200 K. Unfortunately, the crystals pulverised upon cooling below 200 K and several attempts to elucidate the corresponding solid-state structures at 100 K did not yield crystallographic data of sufficient quality to allow definitive structural assignments. Nevertheless, in both cases the diffraction patterns provide clear indications of a structural modulation associated with the low-temperature phase. Consequently, the solid-state structures of compounds 1 and 2 were determined at 200 K instead. Compound 1 (Fig. 2a) crystallises in the triclinic space group *P*1̄ with four molecules within the asymmetric unit, whereas 2 and 3 (Fig. 2b and c) are isomorphous and crystallise in the monoclinic space group *C*2/*c* containing only one molecule in their asymmetric units. Selected bond lengths and angles are summarised in Tables 1 and 2, respectively. Each pnictogen atom in compounds 1–3 features a distorted trigonal-pyramidal coordination geometry (sum of bond angles: 308.7° (1), 304.8° (2), 304.3° (3)), while the tellurium atoms show a bent geometry with Pn–Te–C bond angles ranging from 85.0(9)° to 94.18(9)°, consistent with limited orbital hybridisation and predominantly p-type character of the Te–Pn and Te–C bonding orbitals, as is commonly observed for heavy p-block elements.

**Table 1:** Selected bond lengths [Å] of DMPAs(TePh)_2_ (1), DMPSb(TePh)_2_ (2), and DMPBi(TePh)_2_ (3). For 1, only one of the four residues is described: Te1 = Te1_1, Te2 = Te2_1, As = As1_1, C1 = C25_1, C2 = C31_1, and C3 = C1_1 in cif

	Pn–Te1	Pn–Te2	Pn–C3	Te1–C1	Te2–C2
1	2.6028(4)	2.6013(4)	1.987(3)	2.119(3)	2.129(3)
2	2.7756(4)	2.7768(4)	2.181(4)	2.118(4)	2.128(5)
3	2.8608(9)	2.8579(9)	2.279(10)	2.117(10)	2.213(19)/2.047(19)

**Table 2:** Selected bond angles [°] of DMPAs(TePh)_2_ (1), DMPSb(TePh)_2_ (2), and DMPBi(TePh)_2_ (3). For 1, only one of the four residues is described: Te1 = Te1_1, Te2 = Te2_1, As = As1_1, C1 = C25_1, C2 = C31_1, and C3 = C1_1 in cif

	Te2–Pn–Te1	C3–Pn–Te1	C3–Pn–Te2	Pn–Te1–C1	Pn–Te2–C2
1	97.752(14)	104.32(9)	106.74(9)	94.18(9)	89.05(9)
2	96.100(14)	103.52(11)	105.16(12)	94.05(11)	87.51(13)
3	96.30(3)	103.3(2)	104.7(3)	91.5(2)	86.7(8)/85.0(9)

The structural data of DMPAs(TePh)_2_ (1), DMPSb(TePh)_2_ (2), and DMPBi(TePh)_2_ (3) furthermore exhibit clear periodic trends across the pnictogen series. The Pn–Te bond lengths increase from 2.60 Å (1) to 2.78 Å (2) and 2.86 Å (3), reflecting the atom radius expansion and diminished orbital overlap from As to Bi. The near-equivalence of Pn–Te1 and Pn–Te2 distances within each compound indicates symmetric coordination. The Pn–C3 bonds follow the same trend, lengthening significantly along the series. In contrast, the Te–C bond lengths remain essentially constant (≈2.12 Å), highlighting the structural robustness of the TePh groups and the minor influence of the pnictogen centre on these bonds. The angular parameters further support these conclusions. The Te1–Pn–Te2 and C3–Pn–Te angles vary only slightly, indicating a conserved coordination geometry across the series. More pronounced changes occur at the Pn–Te–C bond angles, which decrease from 1 to 3, pointing to increased geometric flexibility and bond directionality for the heavier pnictogens. The experimentally determined bond lengths are in excellent agreement with previously reported values for the corresponding single bonds in related molecular systems as well as with the sums of the respective single bond covalent radii (C: 0.75 Å; Te: 1.36 Å; As: 1.21 Å; Sb: 1.40 Å; Bi: 1.51 Å).^26,27^ In compound 1, the As–Te distances (2.60 Å) are marginally shorter than those observed for {(IPr)C(Ph)}As(TePh)_2_ (I) (2.62–2.63 Å), whereas the As–C bond in 1 (1.99 Å) is elongated compared to that in I (1.91 Å).^17^ Furthermore, the C–As–Te bond angles in I (≈101.5°) are slightly narrower than those observed in the bistelluridoarsane 1 (≈104°).^17^ The Sb–Te bond lengths in compound 2 are equal to the corresponding single bond reported for Et_2_SbTeEt (2.78 Å).^28^ The Bi–Te bond lengths in compound 3 (2.86 Å) closely match those reported for RBi(TeSi(SiMe_3_)_3_)_2_III (R = CSi(SiMe_3_)_3_: 2.86–2.88 Å, CHSi(SiMe_3_)_2_: 2.84–2.86 Å),^14^ yet are approximately 0.07 Å shorter than the corresponding distances in *o*-CHNDipp-C_6_H_4_Bi(TePh)_2_ (II) (2.90–2.96 Å).^18^ The Te–Bi–Te angle in 3 (96.30(3)°) is consistent with values reported for RBi(TeSi(SiMe_3_)_3_)_2_ (R = CH(SiMe_3_)_2_, 96.49(3)°) and slightly wider than that in the C(SiMe_3_)_3_-substituted analogue (91.19(3)°).^14^ In contrast to the monomeric structure of compound 3, the previously reported bistelluridobismuthane II forms a dimer in the solid state, in which the coordination sphere around the bismuth centre adopts a distorted square-pyramidal geometry owing to the chelating nature of the supporting ligand.^18^

### Reactivity studies

#### Decomposition in solution

Bistelluridopnictanes were frequently reported to display limited stability in solution, with decomposition processes becoming increasingly pronounced for the heavier combinations within groups 15 and 16, particularly bismuth and tellurium. Representative examples include the degradation of *o*-CHNDipp-C_6_H_4_Sb(TePh)_2_ to diphenylditelluride Ph_2_Te_2_ alongside the formation of the corresponding distibene R_2_Sb_2_, whereas the corresponding bismuth analogue decomposed to *o*-CHNDipp-C_6_H_4_Bi(TePh) together with the formation of unidentified products.^18^ Motivated by these findings, we investigated the thermal stability of compounds 1–3 in solution to delineate the decomposition pathways associated with heavier pnictogen–tellurium systems.

**Scheme 2:**
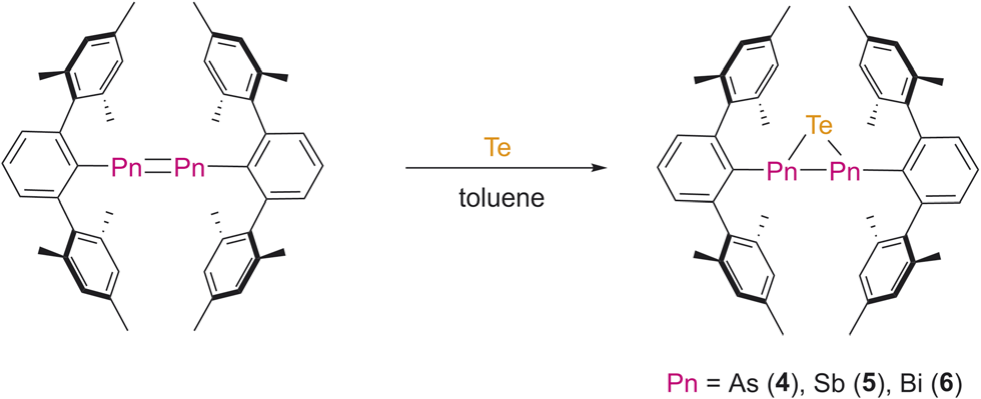
Synthesis of the telluradipnictiranes DMPPn(µ-Te)PnDMP 4–6.

All three compounds exhibit moderate stability in non-polar solvents and undergo noticeable decomposition in benzene and toluene solutions at ambient temperature within 24 to 48 hours. However, temperature-dependent *in situ*^1^H NMR studies with 1–3 in toluene-*d*_8_ solution, conducted from room temperature to 100 °C in 20 °C increments with an equilibration period of 30 min at each temperature, revealed no discernible alterations in their spectral features (see SI, Fig. S29–S31). In contrast, UV-irradiation of the corresponding toluene-*d*_8_ or benzene-*d*_6_ solutions for two hours resulted in the formation of a broad variety of new resonances, pointing towards a light-induced decomposition of 1–3 rather than a thermal activation (see SI, Fig. S32–S34). Under standard storage conditions, in each case, the initial decomposition step involves the formation of Ph_2_Te_2_, which can be readily monitored by ^125^Te and ^1^H NMR spectroscopy (see SI, Fig. S35–S37) and whose formation can be rationalised by a reductive elimination pathway, concomitant with the formation of the corresponding pnictinidenes DMPPn. This is supported by the spectroscopic identification of diarsene DMP_2_As_2_ by *in situ*^1^H NMR spectroscopy. However, although the elimination of Ph_2_Te_2_ is observed for all three compounds 1–3, the emergence of the double-bonded pnictogen species is exclusively detected by NMR for 1. In contrast, the heavier congeners 2 and 3 immediately form other decomposition products which have not yet been clearly identified. Remarkably, from the mixture of decomposition products derived from compound 1, we successfully isolated single crystals of the first example of a three-membered heterocyclic telluradiarsirane DMPAs(µ-Te)AsDMP (4). Given that diphenylditelluride is known to disproportionate with formation of Ph_2_Te and elemental tellurium,^29^ we hypothesised that the isolated telluradiarsirane 4 forms *via* reaction of elemental tellurium with the *in situ* formed diarsene DMP_2_As_2_ (see Scheme 2), which was previously reported for similar doubly bonded heavy group 14 elements^30–32^ and diphosphenes^29^ as well as for reactions of dipnictenes with elemental selenium,^33–36^ respectively. In contrast, the only structurally characterised examples of a telluradistibirane and a telluradibismirane were not obtained *via* direct reaction of the corresponding dipnictenes with elemental tellurium, but instead through the use of *n*Bu_3_PTe as an efficient tellurium-transfer reagent.^37^ Indeed, in a control experiment, we reacted isolated diarsene DMP_2_As_2_ with elemental tellurium, leading to the formation of telluradiarsirane 4, which was isolated as a yellow solid in 60% yield (see Scheme 2).

Extension of this reactivity study to the heavier homologues DMP_2_Pn_2_ (Pn = Sb, Bi) gave the corresponding telluradistibirane 5 and -dibismirane 6 as orange solids in 88% and 68% isolated yield, respectively (see Scheme 2). 5 and 6 represent the second reported members of these compound classes.

**Scheme 3:**
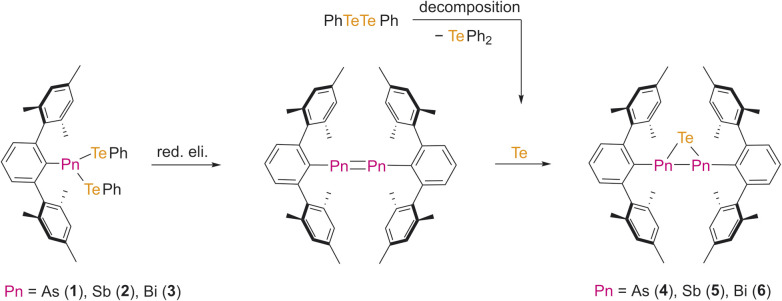
Proposed decomposition pathway of bistelluridopnictanes 1–3.

Interestingly, the isolated heterocycles 5 and 6 form the same decomposition products in benzene and toluene solution as the corresponding bistelluridopnictanes 2 and 3 as evidenced by ^1^H NMR spectroscopy (see SI, Fig. S36 and S37). These findings indicate that they may constitute transient intermediates in the decomposition pathways of 2 and 3, despite not being detected by NMR spectroscopy. The fact that 5 and 6 in marked contrast to 4 could not be isolated from the decomposition studies of 2 and 3 is consistent with the expected higher thermodynamic stability of the arsenic derivatives relative to their heavier pnictogen congeners, whose reduced bond strengths facilitate more rapid and complex decomposition reactions.^38^ This conclusion is further corroborated by the comparatively higher solution stability of the arsenic heterocycle 4, which remains unchanged in benzene-*d*_6_ under ambient conditions for a period of at least 7 days, whereas the corresponding antimony and bismuth analogues 5 and 6 undergo profound decomposition reactions as evidenced by ^1^H NMR spectroscopy (see SI, Fig. S36 and S37). On this basis, we propose a reaction pathway in which the pnictogen centres show a remarkably high flexibility of their formal oxidation states ranging from +III to +I. Compounds 1–3 (Pn in the formal oxidation state +III) first decompose *via* reductive elimination generating diphenylditelluride and the corresponding dipnictene DMP_2_Pn_2_ (Pn in the formal oxidation state +I), which subsequently react in an oxidation reaction with elemental tellurium to form the telluradipnictiranes 4–6 (Pn in the formal oxidation state +II, see Scheme 3). These heterocycles then appear to engage in further decomposition processes and form unidentified products accompanied by black insoluble materials.

#### Spectroscopic characterisation of 4–6

The three-membered heterocyclic telluradipnictiranes DMPPn(µ-Te)PnDMP 4–6 were characterised by multinuclear NMR spectroscopy (see SI, Section I). In all cases, the ^1^H and ^13^C NMR spectra display the expected sets of resonances corresponding to the aromatic and aliphatic protons and carbon nuclei of the DMP substituents. Owing to the inherent asymmetry of the three-membered ring, the two mesityl groups of the DMP ligand are magnetically inequivalent, resulting in two distinct aromatic singlets as well as three methyl singlets in the ^1^H and ^13^C NMR spectra. ^125^Te NMR spectroscopy reveals a single resonance for compound 4 at −251.2 ppm and for compound 5 at −833.2 ppm. In contrast, no ^125^Te resonance was detected for the bismuth analogue 6, most likely due to line broadening induced by the two adjacent bismuth nuclei (*I* = 9/2), a phenomenon previously reported for related systems.^37^ The comparatively higher-frequency chemical shift observed for compound 4 relative to 5 is consistent with the greater electronegativity of arsenic *versus* antimony.^24^ Both resonances fall within the expected range for structurally related three-membered tellurium-containing heterocycles, such as ArP(µ-Te)PAr (7.7 ppm, Ar = 2,4,6-*i*Pr_3_C_6_H_2_),^39^ BbtSb(µ-Te)SbBbt (−622.3 ppm, Bbt = 2,6-CH(SiMe_3_)_2_-4-C(SiMe_3_)_3_C_6_H_2_),^37^ Mes_2_Si(µ-Te)SiMes_2_ (−784 ppm, Mes = 2,4,6-Me_3_C_6_H_2_),^32^ and Ar_2_Sn(µ-Te)SnAr_2_ (−903 ppm, Ar = 2,4,6-*i*Pr_3_C_6_H_2_).^31^

#### Single crystal X-ray structures of 4–6

**Fig. 3:**
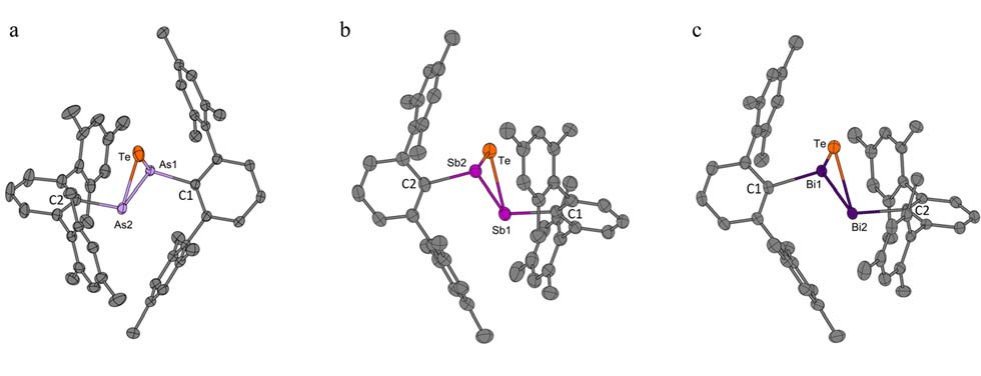
Solid-state molecular structures of (a) DMPAs(µ-Te)AsDMP (4), (b) DMPSb(µ-Te)SbDMP (5), and (c) DMPBi(µ-Te)BiDMP (6). H atoms have been omitted for clarity and displacement ellipsoids are drawn at the 50% probability level.

Single crystals of telluradipnictiranes 4–6 were obtained by storing toluene solutions of the respective compounds at −30 °C for 24 hours. Their solid-state structures are depicted in Fig. 3. All three heterocycles crystallise in the monoclinic space group 14 (4 setting *P*2_1_/*n*, 5 and 6 isomorphous in setting *P*2_1_/*c*) with one molecule in their asymmetric units. The DMP groups adopt transoid orientations to each other in regard to the central three-membered rings. Selected bond lengths and angles are summarised in Table 3.

**Table 3:** Selected bond lengths [Å] and bond angles [°] of DMPAs(µ-Te)AsDMP (4), DMPSb(µ-Te)SbDMP (5), and DMPBi(µ-Te)BiDMP (6)

	Pn1–Te	Pn2–Te	Pn1–Pn2	Pn2–Te–Pn1	Te–Pn2–Pn1	Te–Pn1–Pn2
4	2.5854(3)	2.5815(3)	2.4546(3)	56.73(2)	61.72(2)	61.56(1)
5	2.7736(3)	2.7811(3)	2.8324(3)	61.32(1)	59.21(1)	59.47(1)
6	2.8760(3)	2.8624(3)	2.9832(2)	62.65(1)	58.90(1)	58.45(1)

The telluradipnictirane scaffolds of 4–6 can be closely described as isosceles triangles with bond lengths comparable to previously reported values for the respective single bonds and sum of single bond covalent radii (Te: 1.36 Å, As: 1.21 Å, Sb: 1.40 Å, Bi: 1.51 Å).^26,27^ In this regard, the Sb–Te bond lengths of 5 (2.77–2.78 Å) perfectly agree with the corresponding bond lengths of the only other reported telluradistibirane, BbtSb(µ-Te)SbBbt (2.76–2.77 Å),^37^ whereas the Sb–Sb bond length in 5 (2.83 Å) is 0.05 Å shorter. A similar trend emerges when comparing the telluradibismirane 6 (Bi–Bi: 2.98 Å, Bi–Te: 2.86–2.87 Å) with the only structurally characterised telluradibismirane BbtBi(µ-Te)BiBbt (Bi–Bi: 3.03 Å, Bi–Te: 2.86–2.87 Å).^37^ Notably, the bond angles reported for BbtSb(µ-Te)SbBbt and BbtBi(µ-Te)BiBbt deviate only slightly from those in 5 and 6, with maximum deviations of approximately 1°.^37^ The structural metrics of 4–6 reveal pronounced periodic trends across the pnictogen series. The Pn–Te (2.58 Å (4), 2.78 Å (5), 2.87 Å (6)) and Pn–Pn bond lengths (2.45 Å (4), 2.83 Å (5), 2.98 Å (6)) increase within the series, consistent with the atomic radius expansion from As to Bi. The Te–Pn–Pn bond angles decrease from 4 to 6, whereas the opposite trend is observed for the Pn–Te–Pn bond angle, which was also reported for BbtPn(µ-Te)PnBbt (Pn = Sb, Bi).^37^ These structural trends most likely rely on the increasing size of the pnictogen atoms from 4 to 6.

#### Quantum chemical calculations of 4–6

To gain deeper insight into the electronic structures of telluradipnictiranes 4–6, density functional theory (DFT) calculations were performed (details are given in the SI, Section X). In all cases, the optimized geometries closely reproduce the experimentally determined X-ray structures (Δ*r* < 0.03 Å; Δ*φ* < 1°). Natural bond orbital (NBO) analysis (see SI, Tables S4–S7) identifies the Pn–Te (Pn = As, Sb, Bi) and Pn–Pn bonds as covalent σ-interactions with occupation numbers close to two electrons (ON = 1.94–1.97 |*e*|) and bond indices consistent with single bonds (WBI = 0.92–0.95; MBO = 0.88–0.96). Natural population analysis (NPA) reveals moderate polarisation of the Pn–Te bonds toward Te, which increases along the series from 4 to 6 (P(Te) = 55–62%), accompanied by progressively higher positive charges at the pnictogen centres (As: +0.39 |*e*|; Sb: +0.55–0.56 |*e*|; Bi: +0.61–0.62 |*e*|) and increasingly negative charge at Te (−0.13 to −0.40 |*e*|).

The Pn–Te and Pn–Pn σ-bonds are dominated by p-orbital contributions (92–98%), whereas the pnictogen lone pairs display pronounced s-character that increases from As (78–79%) to Sb (83–84%) and finally Bi (≈90%), consistent with the increasing influence of the inert pair effect down group 15.^4^ In contrast, the Te centre exhibits two electronically distinct lone pairs throughout the series: one predominantly s-type (≈86–87%) and one almost purely p-type (>99%). Frontier molecular orbital analysis reveals a conserved topology for 4–6, with both the HOMO (−5.275 to −5.126 eV) and the LUMO (−1.583 to −1.441 eV) localized at the Pn_2_Te unit (see SI, Fig. S39–S41) rendering 4–6 attractive candidates for one-electron-oxidation and reduction reactions.

#### Synthesis and spectroscopic characterisation of cation 7

**Scheme 4:**
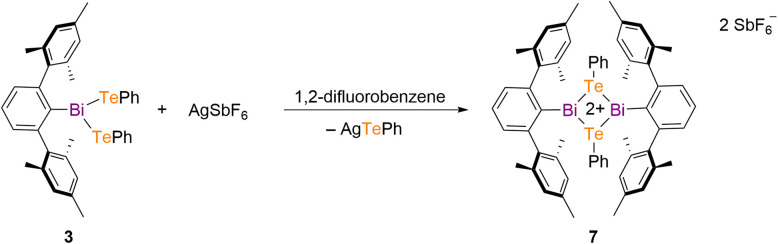
Synthesis of compound 7.

Beginning with the bismuth compound 3, we explored the generation of a radical cation *via* single electron oxidation with AgSbF_6_ based on the observation of an irreversible oxidation event occurring at 0.18 V (1 mM in 1,2-dichlorobenzene solution, measured *versus* Fc/Fc^+^) in cyclic voltammetric studies, while similarly irreversible oxidation reactions were observed for compound 2 at 0.82 V and for compound 1 at 0.46 V (1 mM in THF, measured *versus* Fc/Fc^+^) (CV, SI Section III, Fig. S28). Unexpectedly, instead of the anticipated radical cation, compound 7 containing a cyclic [Bi_2_Te_2_]^2+^ dication was formed, arising from the formal abstraction of a –TePh unit (see Scheme 4). Although a pathway involving initial single electron oxidation followed by Bi–TePh bond homolysis and radical recombination is conceivable, the absence of metallic silver deposition and of detectable amounts of Ph_2_Te_2_ in *in situ*^1^H NMR experiments performed in benzene-*d*_6_ (see SI, Fig. S43), despite the high solubility of Ph_2_Te_2_ in benzene, together with the formation of a yellow precipitate consistent with AgTePh,^40^ argues against PhTe˙ radical recombination as the dominant reaction pathway and instead supports formal –TePh abstraction under the applied reaction conditions. The dication proved to be highly unstable at ambient conditions, even in 1,2-difluorobenzene, and rapidly decomposed to form ditelluride DMP_2_Te_2_ along with other, so far unidentified, byproducts and insoluble black material. Solutions of 7 in tetrahydrofuran, acetonitrile, or dichloromethane decomposed immediately upon dissolution, whereas isolated crystals of 7 can be stored in a glovebox at −30 °C for several weeks. We further attempted to extend these reactivity studies to the arsenic and antimony congeners 1 and 2, but unfortunately, failed to isolate the corresponding dicationic species, most likely due to their insufficient stability in solution.

Compound 7 was characterised by multinuclear NMR spectroscopy (see SI, Section I). The ^1^H and ^13^C NMR spectra of 7 in *o*-C_6_D_4_Cl_2_ show the expected sets of resonances for the DMP and phenyl substituents. ^125^Te NMR spectroscopy reveals one resonance at 157.1 ppm, which is remarkably upfield shifted in comparison to the parent compound 1 (323.31 ppm).

#### Reactivity studies

Since the calculated LUMO of compound 7 (see below) is largely localized on the Bi_2_Te_2_ core, a potential reduction of this framework appeared chemically plausible. To probe the redox behavior of compound 7, cyclic voltammetry measurements were carried out in 1,2-difluorobenzene solution. However, within the accessible potential window, no distinct reversible or irreversible reduction or oxidation events were observed. These results suggest that compound 7 is electrochemically rather inert under the employed conditions, or that potential electron transfer processes are associated with rapid follow-up decomposition pathways that do not result in observable electrochemical features.

Since the polarised Bi–Te bond renders compound 7 potentially attractive for small molecule activation reaction, we performed several reactivity studies with selected substrates. However, exposure of compound 7 to H_2_ did not result in any observable conversion under the applied reaction conditions. In contrast, reactions with polar substrates such as CO, HBPin or N(*n*-Bu)_4_Cl led to rapid consumption of compound 7 and the formation of new species, indicating that the Bi_2_Te_2_ core indeed exhibits chemically accessible reactivity. Unfortunately, the resulting complex reaction mixtures proved to be rather unstable and despite repeated efforts, no crystalline products suitable for structural characterisation could be isolated. Moreover, the corresponding NMR spectra remained broad and poorly resolved due to the pronounced instability and apparent decomposition behavior of the generated species and parent compound.

#### Single crystal X-ray structure of 7

**Fig. 4:**
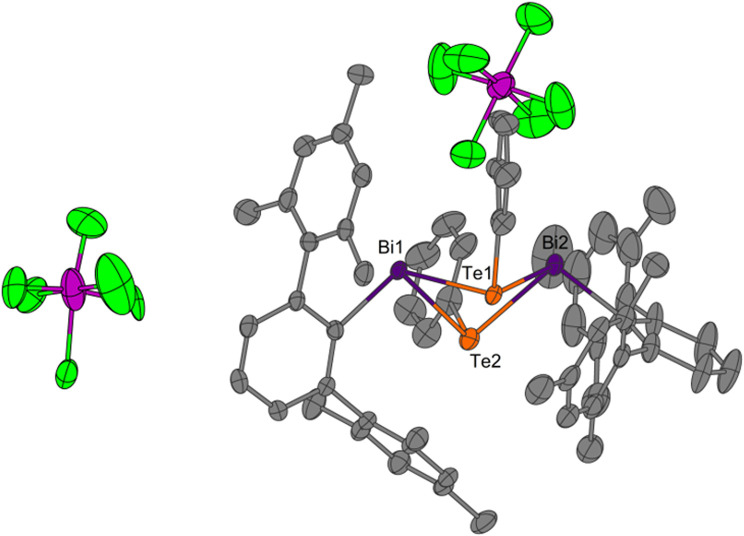
Solid-state molecular structure of [DMPBi(TePh)]_2_[SbF_6_]_2_ (7). H atoms, minor components of the disorder and solvent molecules have been omitted for clarity and displacement ellipsoids are drawn at the 50% probability level. Selected bond lengths [Å]: Bi1–Te1, 2.9606(4); Bi1–Te2, 2.9862(4); Bi2–Te1, 2.9428(4); Bi2–Te2, 2.9762(4); selected bond angles [°]: Te1–Bi1–Te2, 77.69(1); Te2–Bi2–Te1, 78.13(1); Bi1–Te1–Bi2, 81.28(1); Bi2–Te2–Bi1, 81.41(1).

Dark red single crystals of 7 were obtained by slow diffusion of benzene into a solution of 7 in 1,2-difluorobenzene at 8 °C for 12 h (Fig. 4). Prolonged crystallisation times and deviations in the benzene-to-difluorobenzene ratio led to the formation of insoluble black material as the main product.

Compound 7 crystallizes in the triclinic space group *P*1̄ containing one bimetallic dication in its asymmetric unit, accompanied by one benzene and 1,2-difluorobenzene molecule. The central structural motif is represented by a dicationic, folded Bi_2_Te_2_^2+^ four-membered ring, which can be derived from the butterfly-type core missing the transannular Bi–Bi bond. Within this ring, all Bi and Te atoms adopt distorted trigonal pyramidal geometries. While such bonding environments are well documented for homonuclear Pn_4_ frameworks (Pn = P,^41^ As,^42^ Sb^43^ and Bi^44,45^), they have not been reported for the heaviest mixed pnictogen–chalcogen molecular systems (Bi, Te). The four Bi–Te bonds are remarkably uniform, ranging from 2.94–2.99 Å, exceeding the sum of covalent single-bond radii by 0.07 to 0.12 Å (Bi: 1.51 Å, Te: 1.36 Å).^26,27^ These bonds are elongated relative to those in comparable compounds with a trigonal-pyramidal coordinated Bi atom, including (*t*Bu_2_PhSiTe)_3_Bi (2.84 Å)^16^ and Et_2_BiTeEt (2.91 Å).^28^ In contrast, the compound (Et_2_Bi)_2_Te exhibits Bi–Te bonds (2.98 Å)^46^ that agree well with those in our compound 7. The long bond distances were explained due to intermolecular interactions between the molecules. As a consequence of the considerable folding of the four-membered ring (angle between Bi–Te–Bi planes: 111.97(1)°), the Te–Bi–Te bond angles (77.69(1)° and 78.13(1)°) are relatively small for three-coordinate Bi atoms. The Bi–Te–Bi bond angles in 7 are a little wider (81.41(1)° and 81.28(1)°). These angular values agree well with those reported for the folded four-membered Bi_4_ ring in R_4_Bi_4_ (R = CH(SiMe_3_)_2_, Bi–Bi–Bi: 79.93(6)°, 78.97(8)°, 79.82(8)°, and 79.52(8)°, folding angle: 112.8(2)°),^45^ underscoring the structural analogy between these systems.

#### Quantum chemical calculations of 7

To gain further information on the bonding situation of compound 7, quantum chemical calculations were performed (details can be found in the SI, Section X). The geometric parameters of 7 determined by DFT calculations agree well with those of the sc-XRD structure (Δ*r* < 0.05 Å, Δ*φ* < 3°). The NPA charges reveal a strongly polarised charge distribution within the four-membered Bi_2_Te_2_ heterocycle. The majority of the positive charge resides at the bismuth centres (Bi1: +0.90 |*e*|, Bi2: +0.87 |*e*|), whereas the tellurium atoms carry substantially smaller positive charges (Te1: +0.43 |*e*|, Te2: +0.45 |*e*|). This distribution reflects the higher electronegativity of tellurium^24^ and indicates significant charge localisation at the electropositive bismuth atoms within the overall dicationic framework. Consistent with this picture, NBO analysis shows that the Bi–Te bonding orbitals are primarily composed of p-type atomic orbitals at both atoms (>92%), corresponding to σ-type p–p overlap as the dominant bonding interaction (ON = 1.94–1.95 |*e*|). The bonding electron density is polarised toward tellurium, which contributes approximately 64–68% to the bonding orbitals, while the bismuth atoms contribute only 29–35%. This pronounced polarisation is fully consistent with the NPA charge distribution and highlights the heteropolar character of the Bi–Te bonds. The NBO analysis (see SI, Table S7) further reveals well-localised electron lone pairs at the bismuth centres with an overwhelming s-character of approximately 90%, which is characteristic of the stereochemically active 6s^2^ inert pair commonly observed in Bi(iii) compounds.^4^ The tellurium atoms likewise possess one localised lone pair each with mainly s- (76–77%) but larger p-type character (23%). The bonding metrics obtained from Mayer and Wiberg bond order analyses further support this bonding description. The Bi–Te interactions exhibit MBOs between 0.74 and 0.85, while the corresponding WBIs range from 0.73 to 0.81, consistent with moderately strong but polarised Bi–Te single bonds. The slight variation within this range likely reflects subtle differences in orbital overlap and local geometric constraints within the four-membered Bi_2_Te_2_ core. Frontier orbital analysis shows that the HOMO (−10.75 eV) is predominantly ligand-centred, whereas the LUMO (−7.57 eV) is located at the four-membered heterocycle (see SI, Fig. S24). The lone pairs at the Bi and Te atoms are located at significantly lower energies and therefore remain electronically inactive within the frontier orbital manifold.

## Conclusions

This study establishes a unified and modular synthetic strategy that enables reliable access to a complete series of neutral, well-defined pnictogen–tellurium compounds of the type DMPPn(TePh)_2_ (Pn = As (1), Sb (2), Bi (3)), a compound class that has long remained synthetically elusive due to the intrinsic lability of multiple Pn–Te bonds. The salt elimination route presented herein overcomes limitations associated with low-valent pnictogen precursors and delivers synthetically robust, isolable derivatives in reproducible yields, thereby providing a systematic platform for comparing structure, bonding, and reactivity across the heavier pnictogen series.

Comprehensive spectroscopic and crystallographic analyses of 1–3 reveal clear periodic trends in Pn–Te bonding metrics and coordination geometries. Importantly, controlled decomposition studies uncover a unified reactivity manifold in which all compounds undergo initial reductive elimination reactions of diphenylditelluride, followed by divergent, pnictogen-specific reaction pathways. These experiments allowed the isolation of the first telluradiarsirane DMPAs(µ-Te)AsDMP 4 and the corresponding Sb (5) and Bi (6) congeners, representing only the second reported examples of such three-membered heterocyclis telluradipnictiranes. Their structural analysis highlights pronounced periodic trends and demonstrates that these strained heterocycles may constitute key, previously overlooked intermediates in the breakdown of heavy Pn–Te compounds.

Beyond neutral species, the bistelluridobismuthane 3 gives rise to the unprecedented dicationic, folded Bi_2_Te_2_ ring 7, a structural motif known previously for homonuclear four-membered pnictogen systems but not for mixed heavy Pn–Ch molecular frameworks. This finding underscores the propensity of heavy main-group elements to assemble into complex architectures through subtle interplay of electronic and steric effects.

Overall, the results presented here significantly broaden the accessible chemical space of heavy pnictogen–tellurium compounds, providing new molecular benchmarks, previously unknown heterocyclic motifs, and reactivity principles that collectively advance the fundamental understanding of Pn–Ch bonding. The modularity of the synthetic approach and the rich reactivity of the resulting compounds open pathways toward designed precursors for functional chalcogenide materials, as well as new opportunities to probe oxidation-state flexibility, multimetal cooperation, and electron-rich bonding environments in the heavy main group element chemistry. Continued exploration of these systems promises to uncover further unconventional structural motifs and mechanistic insights central to the chemistry of the heavier p-block elements.

## Experimental section

### General procedure

All work was performed under inert gas conditions using standard Schlenk and glovebox techniques. Argon gas (purity grade 5.0, Air Liquide) was purified by passing through columns containing molecular sieves (4 Å), P_4_O_10_ and finally BTS catalyst (MBraun). Toluene and *n*-hexane were dried by use of a Solvent Purification System (MBraun), while benzene and tetrahydrofuran were refluxed over Na/K alloys and 1,3-difluorobenzene over CaH_2_. All dried solvents were stored in flasks with molecular sieves (4 Å) under inert gas conditions. The anhydrous nature of the solvents was verified by Karl-Fischer titration. Elemental analyses were performed at the Elementaranalyse Labor of University of Duisburg-Essen. Melting points were measured using a Thermo Scientific 9300 apparatus. Cyclic voltammetry studies were performed in a glovebox using a Metrohm Autolab PGSTAT 204 potentiostat with a three electrodes setup consisting of a Pt disc (*d* ¼ 1 mm) working electrode, Pt wire counter electrode, and Ag wire pseudoreference electrode, and ferrocene as internal standard. Positive feedback compensation was used to reduce solvent resistance effects.

Elemental tellurium powder (99.999%, TCI), phenyllithium (1.9 M in dibutyl ether, Merck) and AgSbF_6_ (98%, Merck) were commercially available.

DMPPnCl_2_ (Pn = As, Sb, Bi) and DMP_2_Pn_2_ (Pn = As, Sb, Bi) were prepared according to literature methods.^47^

### Spectroscopic methods

^1^H, ^13^C{^1^H} and ^125^Te{^1^H} NMR spectra were recorded using a Bruker Avance III 600 MHz, Bruker AV NEO 400 MHz or Jeol JNM-ECZL 400 MHz spectrometer. Chemical shifts (*δ*) are given in ppm and are referenced to the residual signal of the solvent (benzene: ^1^H: *δ* = 7.16; ^13^C: *δ* = 128.06; toluene: ^1^H: *δ* = 7.09, 7.01, 6.97, 2.08; ^13^C: *δ* = 137.48, 128.87, 127.96, 125.12, 20.43; 1,2-dichlorobenzene: ^1^H: *δ* = 7.19, 6.93; ^13^C: *δ* = 132.39, 130.04, 127.19). ^125^Te{^1^H} NMR spectra were referenced using IUPAC recommendation of NMR nomenclature.^48^

IR spectra were recorded using an ALPHA-T FT-IR spectrometer equipped with a single-reflection ATR sampling module, which was placed in a glovebox to measure under inert gas conditions.

#### Synthesis of DMPAs(TePh)_2_ (1)

To a stirred suspension of elemental tellurium (293 mg, 2.29 mmol, 2.0 equiv.) in anhydrous THF (20 mL), a solution of phenyllithium in dibutyl ether (1.2 mL, 1.9 M, 2.29 mmol, 2.0 equiv.) was added at room temperature. The reaction mixture was stirred until a persistent red colouration developed, indicating the formation of the organotellurium intermediate. The resulting suspension was subsequently filtered, and the red filtrate was added dropwise to a cooled solution of DMPAsCl_2_ (525 mg, 1.14 mmol, 1.0 equiv.) in THF (30 mL) at −78 °C, yielding an orange solution. The reaction mixture was warmed to room temperature within 12 h. After completion, the solvent was removed under reduced pressure, and the crude product was extracted with hot *n*-hexane (50 mL). Upon cooling, compound 1 was isolated as orange crystals suitable for sc-XRD analysis (yield: 415 mg, 46%). M.p.: 198 °C. Elemental anal. calcd. (%) for C_36_H_35_AsTe_2_ (797.80 g mol^−1^): C, 54.20; H, 4.42. Found: C, 54.58; H, 4.45. ^1^H NMR (400 MHz, *d*_8_-toluene, 298 K): *δ* = 7.33–7.28 (m, 4H, Ar*H*^*o*-Ph^), 7.12 (t, ^3^*J*_HH_ = 7.5 Hz, 1H, Ar*H*^*p*-DMP^), 6.85–6.79 (m, 2H, Ar*H*^*p*-Ph^), 6.82 (d, ^3^*J*_HH_ = 7.5 Hz, 2H, Ar*H*^*m*-DMP^), 6.72 (s, 4H, Ar*H*^Mes^), 6.70–6.64 (m, 4H, Ar*H*^*m*-Ph^), 2.24 (s, 12H, *o*-C*H*_3_), 2.14 (s, 6H, *p*-C*H*_3_). ^13^C{^1^H} NMR (101 MHz, *d*_8_-toluene, 298 K): *δ* = 146.66, 139.53, 138.77, 138.01, 137.79, 136.64, 130.21, 129.99, 129.31, 128.96, 127.25, 114.76, 21.91, 21.21.^125^Te{^1^H} NMR (189 MHz, *d*_8_-toluene, 298 K): *δ* = 620.2. ATR-IR: *v* = 3046, 2967, 2909, 2850, 2728, 1609, 1570, 1469, 1443, 1431, 1317, 1291, 1256, 1230, 1172, 1151, 1096, 1087, 1064, 1023, 1014, 995, 965, 898, 850, 803, 772, 730, 689, 685, 650, 609, 589, 574, 542, 501, 478, 451 cm^−1^.

#### Synthesis of DMPSb(TePh)_2_ (2)

To a stirred suspension of elemental tellurium (243 mg, 1.90 mmol, 2.0 equiv.) in anhydrous THF (15 mL), a solution of phenyllithium in dibutyl ether (1.0 mL, 1.9 M, 1.90 mmol, 2.0 equiv.) was added at room temperature. The reaction mixture was stirred until a persistent red colouration developed, indicating the formation of the organotellurium intermediate. The resulting suspension was subsequently filtered, and the red filtrate was added dropwise to a cooled solution of DMPSbCl_2_ (480 mg, 0.95 mmol, 1.0 equiv.) in THF (20 mL) at −78 °C, resulting in an orange solution. The reaction mixture was warmed to room temperature overnight. After completion, the solvent was removed under reduced pressure, and the crude product was extracted with hot *n*-hexane (50 mL). Upon cooling, compound 2 was isolated as orange crystals suitable for sc-XRD analysis (yield: 384 mg, 48%). M.p.: 211 °C. Elemental anal. calcd. (%) for C_36_H_35_SbTe_2_ (844.64 g mol^−1^): C, 51.19; H, 4.18. Found: C, 51.26; H, 4.22. ^1^H NMR (400 MHz, C_6_D_6_, 298 K): *δ* = 7.57–7.52 (m, 4H, Ar*H*^*o*-Ph^), 7.14 (t, ^3^*J*_HH_ = 7.6 Hz, 1H, Ar*H*^*p*-DMP^), 6.89 (d, ^3^*J*_HH_ = 7.5 Hz, 2H, Ar*H*^*m*-DMP^), 6.87–6.80 (m, 2H, Ar*H*^*p*-Ph^), 6.76 (s, 4H, Ar*H*^Mes^), 6.71–6.64 (m, 4H, Ar*H*^*m*-Ph^), 2.26 (s, 12H, *o*-C*H*_3_), 2.13 (s, 6H, *p*-C*H*_3_). ^13^C{^1^H} NMR (101 MHz, C_6_D_6_, 298 K): *δ* = 149.35, 140.54, 140.41, 138.03, 136.48, 130.54, 129.49, 129.20, 127.36, 111.66, 21.97, 21.23. ^125^Te{^1^H} NMR (189 MHz, *d*_8_-toluene, 298 K): *δ* = 311.8. ATR-IR: *v* = 3046, 2967, 2909, 2850, 2728, 1609, 1570, 1469, 1443, 1431, 1379, 1317, 1291, 1256, 1230, 1172, 1151, 1096, 1087, 1064, 1023, 1014, 995, 965, 898, 850, 803, 730, 689, 652, 609, 589, 574, 542, 501, 451 cm^−1^.

#### Synthesis of DMPBi(TePh)_2_ (3)

To a stirred suspension of elemental tellurium (51 mg, 0.40 mmol, 2.0 equiv.) in anhydrous THF (5 mL), a solution of phenyllithium in dibutyl ether (0.21 mL, 1.9 M, 0.40 mmol, 2.0 equiv.) was added at room temperature. The reaction mixture was stirred until a persistent red colouration developed, indicating the formation of the organotellurium intermediate. The resulting suspension was subsequently filtered, and the red filtrate was added dropwise to a cooled solution of DMPBiCl_2_ (119 mg, 0.20 mmol, 1.0 equiv.) in THF (5 mL) at −78 °C, resulting in a red solution. The reaction mixture was warmed to room temperature overnight. After completion, the solvent was removed under reduced pressure, and the crude product was extracted with hot *n*-hexane (10 mL). Upon cooling, compound 3 was isolated as red crystals suitable for sc-XRD analysis (yield: 90 mg, 48%). M.p.: decomp. 160 °C. Elemental anal. calcd. (%) for C_36_H_35_BiTe_2_ (931.86 g mol^−1^): C, 46.40; H, 3.79. Found: C, 46.62; H, 3.95. ^1^H NMR (600 MHz, *d*_8_-toluene, 298 K): *δ* = 7.49–7.44 (m, 4H, Ar*H*^*o*-Ph^), 7.25 (dd, ^3^*J*_HH_ = 8.1, 6.7 Hz, 1H, Ar*H*^*p*-DMP^), 7.20–7.17 (m, 2H, Ar*H*^*m*-DMP^), 6.83–6.78 (m, 2H, Ar*H*^*p*-Ph^), 6.74 (s, 4H, Ar*H*^Mes^), 6.70–6.65 (m, 4H, Ar*H*^*m*-Ph^), 2.21 (s, 12H, *o*-C*H*_3_), 2.13 (s, 6H, *p*-C*H*_3_). ^13^C{^1^H} NMR (101 MHz, C_6_D_6_, 298 K): *δ* = 151.14, 141.92, 141.60, 138.04, 136.19, 130.50, 129.75, 129.47, 128.99, 127.55, 108.64, 21.96, 21.17. ^125^Te{^1^H} NMR (189 MHz, *d*_8_-toluene, 298 K): *δ* = 323.3. ATR-IR: *v* = 3047, 2964, 2931, 2908, 2848, 2731, 1609, 1568, 1469, 1444, 1429, 1376, 1322, 1295, 1260, 1175, 1154, 1094, 1086, 1059, 1030, 1014, 997, 906, 848, 800, 730, 691, 652, 585, 569, 546, 505, 451, 410 cm^−1^.

#### Synthesis of DMPAs(µ-Te)AsDMP (4)

A suspension of elemental tellurium (8.4 mg, 0.065 mmol, 1.0 equiv.) and diarsene DMP_2_As_2_ (50 mg, 0.065 mmol, 1.0 equiv.) in toluene (2 mL) was heated at 90 °C for seven days under inert atmosphere. Upon completion, the reaction mixture was cooled to room temperature and filtered. The solvent was removed under reduced pressure, and the resulting solid was washed with *n*-hexane (2 mL), affording the product as a yellow powder (yield: 35 mg, 60%). Single crystals suitable for X-ray diffraction analysis were obtained by cooling a toluene solution to −30 °C. M.p.: decomp. 144 °C. Elemental anal. calcd. (%) for C_48_H_50_As_2_Te (904.37 g mol^−1^): C, 63.75; H, 5.57. Found: C, 63.97; H, 5.84. ^1^H NMR (400 MHz, C_6_D_6_, 298 K): *δ* = 6.95–6.90 (m, 2H, Ar*H*^*p*-DMP^), 6.94 (s, 4H, Ar*H*^Mes^) 6.88 (s, 4H, Ar*H*^Mes^), 6.70 (d, ^3^*J*_HH_ = 7.5 Hz, 4H, Ar*H*^*m*-DMP^), 2.28 (s, 12H, –C*H*_3_), 2.01 (s, 12H, –C*H*_3_), 1.95 (s, 12H, –C*H*_3_). ^13^C{^1^H} NMR (101 MHz, C_6_D_6_, 298 K): *δ* = 146.48, 141.92, 140.14, 137.07, 137.03, 136.20, 129.28, 128.96, 128.80, 128.67, 22.03, 21.68, 21.39. ^125^Te{^1^H} NMR (189 MHz, C_6_D_6_, 298 K): *δ* = −251.2. ATR-IR: *v* = 3047, 3002, 2964, 2931, 2908, 2848, 2731, 1609, 1568, 1479, 1469, 1444, 1429, 1376, 1322, 1260, 1237, 1175, 1154, 1094, 1086, 1059, 1030, 1014, 848, 800, 780, 730, 691, 652, 614, 585, 569, 546, 523, 505, 451, 410 cm^−1^.

#### Synthesis of DMPSb(µ-Te)SbDMP (5)

A suspension of elemental tellurium (45 mg, 0.35 mmol, 1.0 equiv.) and distibene DMP_2_Sb_2_ (30 mg, 0.35 mmol, 1.0 equiv.) in toluene (2 mL) was heated at 90 °C for 24 hours under inert atmosphere. Upon completion, the reaction mixture was cooled to room temperature and filtered. The solvent was removed under reduced pressure, and the resulting solid was washed with *n*-hexane (2 mL), affording the product as an orange powder (yield: 153 mg, 88%). Single crystals suitable for X-ray diffraction analysis were obtained by cooling a toluene solution to −30 °C. M.p.: decomp. 155 °C. Elemental anal. calcd. (%) for C_48_H_50_Sb_2_Te (998.05 g mol^−1^): C, 57.77; H, 5.05. Found: C, 58.03; H, 5.12. ^1^H NMR (600 MHz, C_6_D_6_, 298 K): *δ* = 6.95 (t, ^3^*J*_HH_ = 7.5 Hz, 2H, Ar*H*^*p*-DMP^), 6.88 (s, 8H, Ar*H*^Mes^), 6.71 (d, ^3^*J*_HH_ = 7.5 Hz, 4H, Ar*H*^*m*-DMP^), 2.26 (s, 12H, –C*H*_3_), 2.01 (s, 12H, –C*H*_3_), 1.98 (s, 12H, –C*H*_3_). ^13^C{^1^H} NMR (101 MHz, C_6_D_6_, 298 K): *δ* = 149.18, 142.05, 137.30, 136.65, 136.31, 129.47, 129.34, 129.15, 129.05, 128.57, 22.04, 21.71, 21.35. ^125^Te{^1^H} NMR (189 MHz, C_6_D_6_, 298 K): *δ* = −833.2. ATR-IR: *v* = 3047, 2964, 2931, 2908, 2848, 2731, 1609, 1568, 1479, 1469, 1444, 1429, 1376, 1322, 1260, 1237, 1175, 1154, 1094, 1086, 1059, 1030, 1014, 906, 848, 800, 780, 730, 691, 652, 585, 569, 546, 505, 451, 410 cm^−1^.

#### Synthesis of DMPBi(µ-Te)BiDMP (6)

A suspension of elemental tellurium (6 mg, 0.05 mmol, 1.0 equiv.) and dibismuthene DMP_2_Bi_2_ (50 mg, 0.05 mmol, 1.0 equiv.) in toluene (2 mL) was heated at 90 °C for four days under inert atmosphere. Upon completion, the reaction mixture was cooled to room temperature and filtered. The solvent was removed under reduced pressure, and the resulting solid was washed with *n*-hexane (2 mL), affording the product as a red powder (yield: 40 mg, 68%). Single crystals suitable for X-ray diffraction analysis were obtained by cooling a benzene solution to 7 °C. M.p.: decomp. 123 °C. Elemental anal. calcd. (%) for C_48_H_50_Bi_2_Te (1172.49 g mol^−1^): C, 49.17; H, 4.30. Found: C, 49.38; H, 4.61. ^1^H NMR (600 MHz, C_6_D_6_, 298 K): *δ* = 7.00 (dd, ^3^*J*_HH_ = 7.7, 7.2 Hz, 2H, Ar*H*^*p*-DMP^), 6.89 (s, 4H, Ar*H*^Mes^), 6.87 (d, ^3^*J*_HH_ = 7.5 Hz, 4H, Ar*H*^*m*-DMP^), 6.86 (s, 4H, Ar*H*^Mes^), 2.29 (s, 12H, –C*H*_3_), 2.05 (s, 12H, –C*H*_3_), 1.96 (s, 12H, –C*H*_3_). ^13^C{^1^H} NMR (151 MHz, C_6_D_6_, 298 K): *δ* = 151.16, 144.42, 137.31, 136.91, 136.22, 129.34, 128.91, 128.69, 128.35, 127.35, 22.15, 21.90, 21.30. ATR-IR: *v* = 3025, 2999, 2964, 2915, 2845, 1609, 1556, 1475, 1446, 1373, 1262, 1087, 1023, 944, 889, 848, 796, 737, 688, 659, 618, 571, 478, 426 cm^−1^.

#### Synthesis of [DMPBi(TePh)]_2_[SbF_6_]_2_ (7)

A solution of DMPBi(TePh)_2_ (400 mg, 0.43 mmol, 1 equiv.) and AgSbF_6_ (147.5 mg, 0.43 mmol, 1 equiv.) in 5 mL of 1,2-difluorobenzene was stirred at room temperature for 2 hours and subsequently filtered. The resulting solution was concentrated under reduced pressure to a volume of approximately 1 mL and layered with 1.5 mL of benzene. Slow diffusion at 8 °C afforded dark red crystals of 7 suitable for single crystal X-ray diffraction (yield: 355 mg, 86%). M.p.: decomp. 121 °C. Elemental anal. calcd. (%) for C_48_H_50_Bi_2_Te_2_Sb_2_F_12_ (1925.8 g mol^−1^): C, 32.54; H, 2.84. Found: C, 32.63; H, 2.88. ^1^H NMR (400 MHz, *o*-C_6_D_4_Cl_2_, 298 K): *δ* = 7.72 (d, ^3^*J*_HH_ = 6.9 Hz, 4H, Ar*H*^*o*-Ph^), 7.65 (dd, ^3^*J*_HH_ = 8.3, 6.5 Hz, 2H, Ar*H*^*p*-DMP^), 7.53 (t, ^3^*J*_HH_ = 7.7 Hz, 4H, Ar*H*^*m*-Ph^), 7.43 (d, ^3^*J*_HH_ = 7.6 Hz, 4H, Ar*H*^*m*-DMP^), 7.27 (t, ^3^*J*_HH_ = 7.4 Hz, 2H, Ar*H*^*p*-Ph^), 6.57 (s, 8H, Ar*H*^Mes^), 2.14 (s, 12H, –C*H*_3_), 1.84 (s, 24H, –C*H*_3_). ^13^C{^1^H} NMR (101 MHz, *o*-C_6_D_4_Cl_2_, 298 K): *δ* = 149.31, 141.32, 138.93, 133.02, 136.86, 134.85, 132.39, 132.09, 131.79, 130.56, 124.65, 106.38, 21.11, 21.03. ^125^Te{^1^H} NMR (189 MHz, *o*-C_6_D_4_Cl_2_, 298 K): *δ* = 157.1. ATR-IR: *v* = 3051, 2939, 2916, 2908, 2850, 1607, 1568, 1471, 1448, 1435, 1378, 1295, 1264, 1179, 1160, 1094, 1063, 1051, 1030, 995, 865, 844, 803, 734, 687, 654, 585, 556, 505, 447, 412 cm^−1^.

## Author contributions

Conceptualisation: C. E. R. v. H. and S. S. Supervision: S. S. Experimental methodology: C. E. R. v. H. and S. S. Crystallographic methodology: C. W. Synthesis: C. E. R. v. H. Investigation: C. E. R. v. H. Computation: L. K. Writing – original draft: C. E. R. v. H. Writing – review & editing: all.

## Conflicts of interest

There are no conflicts to declare.

## Supplementary Material

SC-017-D6SC02907H-s001

SC-017-D6SC02907H-s002

## Data Availability

Crystallographic data in cif format and other experimental data, *e.g.*, NMR and IR spectra, cyclic voltammograms as well as details from quantum chemical calculations supporting this article have been included as part of the supplementary information (SI). Supplementary information is available. See DOI: https://doi.org/10.1039/d6sc02907h. CCDC 2540651 (1), 2540652 (2), 2540653 (3), 2540654 (4), 2540676 (5), 2540677 (6) and 2540678 (7) contain the supplementary crystallographic data for this paper.^49*a*–*g*^
