# Supplementary material for: Bistelluridopnictanes as gateways to telluradipnictiranes and a cyclic [Bi2Te2]2+ dication
Source: Chem Sci. 2026 Jun 29. Online ahead of print. doi: 10.1039/d6sc02907h (PMC13321814; doi:10.1039/d6sc02907h)
Supplement: SC-OLF-D6SC02907H-s001 [file SC-OLF-D6SC02907H-s001.pdf]

Supplementary Information (SI) for Chemical Science.
This journal is © The Royal Society of Chemistry 2026

Supporting Information

Bistelluridopnictanes as Gateways to Telluradipnictiranes and a cyclic $[Bi_2Te_2]^{2+}$ Dication

Charlotte E. R. van Halteren[a], Christoph Wölper[a], Leon Kapp[a], Stephan Schulz[a,b]*

[a] C. E. R. van Halteren, Dr. C. Wölper, L. Kapp, Prof. Dr. S. Schulz, Institute of Inorganic Chemistry, University of Duisburg-Essen, Universitätsstraße 5-7, 45141 Essen (Germany).

[b] Center for Nanointegration Duisburg-Essen (CENIDE), University of Duisburg-Essen, Carl-Benz-Straße 199, 47057 Duisburg (Germany).

Corresponding author: stephan.schulz@uni-due.de

Content

I. Spectroscopic characterization

Figure S1-S3. ^{1}H, $^{13}C\{^{1}H\}$ and ^{125}Te NMR spectra of DMPAs(TePh)$_2$ (**1**). S4-S5

Figure S4. IR spectrum of DMPAs(TePh)$_2$ (**1**) S5

Figure S5-S7. ^{1}H, $^{13}C\{^{1}H\}$ and ^{125}Te NMR spectra of DMPSb(TePh)$_2$ (**2**). S6-S7

Figure S8. IR spectrum of DMPSb(TePh)$_2$ (**2**). S7

Figure S9-S11. ^{1}H, $^{13}C\{^{1}H\}$ and ^{125}Te NMR spectra of DMPBi(TePh)$_2$ (**3**). S8-S9

Figure S12. IR spectrum of DMPBi(TePh)$_2$ (**3**). S9

Figure S13-S15. ^{1}H, $^{13}C\{^{1}H\}$ and ^{125}Te NMR spectra of DMPAs(μ-Te)AsDMP (**4**). S10-S11

Figure S16. IR spectrum of DMPAs(μ-Te)AsDMP (**4**). S11

Figure S17-S19. ^{1}H, $^{13}C\{^{1}H\}$ and ^{125}Te NMR spectra of DMPSb(μ-Te)SbDMP (**5**). S12-S13

Figure S20. IR spectrum of DMPSb(μ-Te)SbDMP (**5**). S13

Figure S21-S22. ^{1}H and $^{13}C\{^{1}H\}$ NMR spectra of DMPBi(μ-Te)BiDMP (**6**). S14

Figure S23. IR spectrum of DMPBi(μ-Te)BiDMP (**6**). S15

Figure S24-S26. ^{1}H, $^{13}C\{^{1}H\}$ and ^{125}Te NMR spectra of [DMPBi(TePh)]$_2$[SbF$_6$]$_2$ (**7**). S16-S17

Figure S27. IR spectrum of [DMPBi(TePh)]$_2$[SbF$_6$]$_2$ (**7**). S17

Single Crystal X-ray Diffraction (sc-XRD)

Table S1. Crystal data and structure refinement of compounds **1**, **2** and **3**. S19

Table S2. Crystal data and structure refinement of compounds **4**, **5** and **6**. S20

Table S3. Crystal data and structure refinement of compound **7**. S21

Cyclic Voltammetry (CV)

Figure S28. Cyclic voltammograms of **1**-**3** in THF (**1**, **2**) or 1,2-difluorobenzene (**3**) (0.01 M *n*-Bu$_4$N[B(C$_6$F$_5$)$_4$] as a supporting electrolyte, 0.1 Vs^{-1}, vs Fc/Fc^{+}). The cycle with Fc/Fc^{+} couple has been removed for clarity. S22

IX. Decomposition studies

Figure S29. Temperature-dependent *in situ* ^{1}H NMR spectra of compound **1**. S23

Figure S30. Temperature-dependent *in situ* ^{1}H NMR spectra of compound **2**. S24

Figure S31. Temperature-dependent *in situ* ^{1}H NMR spectra of compound **3**. S25

Figure S32. ^{1}H NMR spectra of compound **1** before and after UV irradiation. S26

Figure S33. ^{1}H NMR spectra of compound **2** before and after UV irradiation. S26

Figure S34. ^{1}H NMR spectra of compound **3** before and after UV irradiation. S27

Figure S35. Comparison of ^{1}H NMR spectrum of compound **1** with its crude decomposition ^{1}H NMR spectrum together with ^{1}H NMR spectra of successfully identified and isolated decomposition products. All spectra are measured in benzene-d_6 at room temperature. S27

Figure S36. Comparison of ^{1}H NMR spectrum of compound **2** with its crude decomposition ^{1}H NMR spectrum together with ^{1}H NMR spectra of successfully identified diphenylditelluride and other decomposition products. All spectra are measured in benzene-d_6 at room temperature. S28

Figure S37. Comparison of ^{1}H NMR spectrum of compound **3** with its crude decomposition ^{1}H NMR spectrum together with ^{1}H NMR spectra of successfully identified diphenylditelluride and other decomposition products. All spectra are measured in benzene-d_6 at room temperature. S29

Figure S38. ^{1}H NMR spectrum of DMPTeTeDMP. S29

X. Quantum chemical calculations

Figure S39. Calculated HOMO (left) and LUMO (right) of **4**. S30

Figure S40. Calculated HOMO (left) and LUMO (right) of **5**. S30

Figure S41. Calculated HOMO (left) and LUMO (right) of **6**. S30

Figure S42. Calculated HOMO (left) and LUMO (right) of **7**. S31

Table S4. NPA atomic charges (q, e), occupation numbers (ON, |e|) of the bonds according to NBO, Wiberg bond index (WBI), mayer bond order (MBO), bond polarization (P) and bond orbital character (OC) for the optimized geometry of **4**. S31

Table S5. NPA atomic charges (q, e), occupation numbers (ON, |e|) of the bonds according to NBO, Wiberg bond index (WBI), Mayer bond order (MBO), bond polarization (P) and bond orbital character (OC) for the optimized geometry of **5**. S31

Table S6. NPA atomic charges (q, e), occupation numbers (ON, |e|) of the bonds according to NBO, Wiberg bond index (WBI), Mayer bond order (MBO), bond polarization (P) and bond orbital character (OC) for the optimized geometry of **6**. S31

Table S7. NPA atomic charges (q, e), occupation numbers (ON, |e|) of the bonds according to NBO, Wiberg bond index (WBI), Mayer bond order (MBO), bond polarization (P) and bond orbital character (OC) for the optimized geometry of **7**. S31

Table S8. Cartesian coordinates (x,y,z) for the optimized geometry of **4**. S32

Table S9. Cartesian coordinates (x,y,z) for the optimized geometry of **5**. S34

Table S10. Cartesian coordinates (x,y,z) for the optimized geometry of **6**. S36

Table S11. Cartesian coordinates (x,y,z) for the optimized geometry of **7**. S38

XI. *In situ* NMR study of the formation of compound 7

Figure S43. *In situ* ^{1}H NMR spectrum of the reaction of compound **3** with $AgSbF_6$ in benzene-d_6 measured at room temperature. After addition of $AgSbF_6$ a mixture of a yellow and red precipitate formed. As shown here, the resulting supernatant features almost no resonances except from solvent residues. S40

XII. References S41

I. Spectroscopic characterization

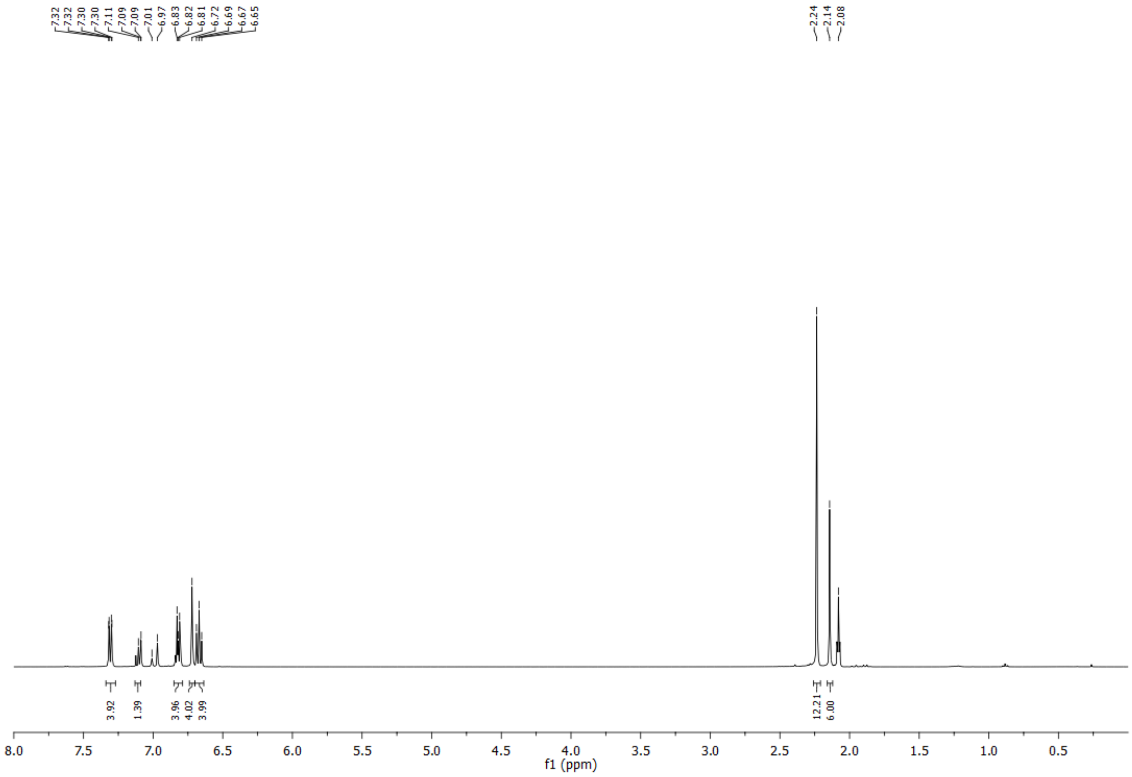

Figure S1. ^{1}H NMR spectrum of DMPAs(TePh)$_2$ (**1**) in toluene-*d₈* at room temperature.

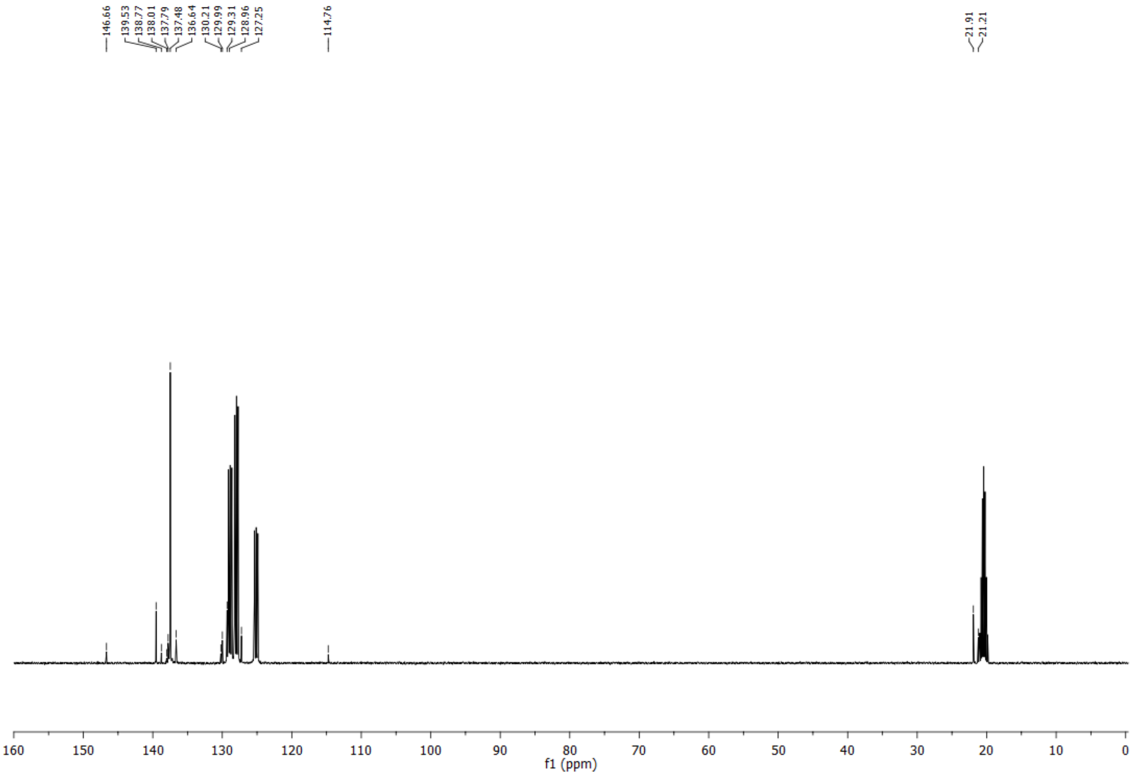

Figure S2. $^{13}C\{^{1}H\}$ NMR spectrum of DMPAs(TePh)$_2$ (**1**) in toluene-d_8 at room temperature.

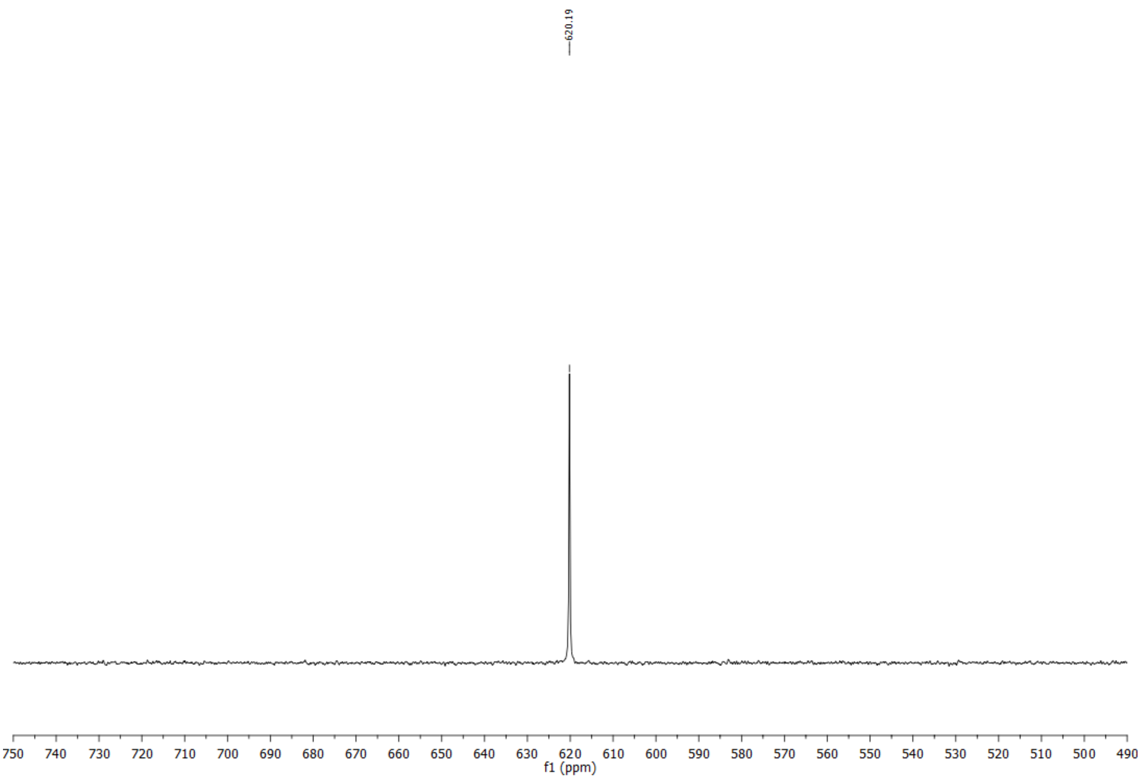

Figure S3. ^{125}Te NMR spectrum of DMPAs(TePh)$_2$ (**1**) in toluene-d_8 at room temperature.

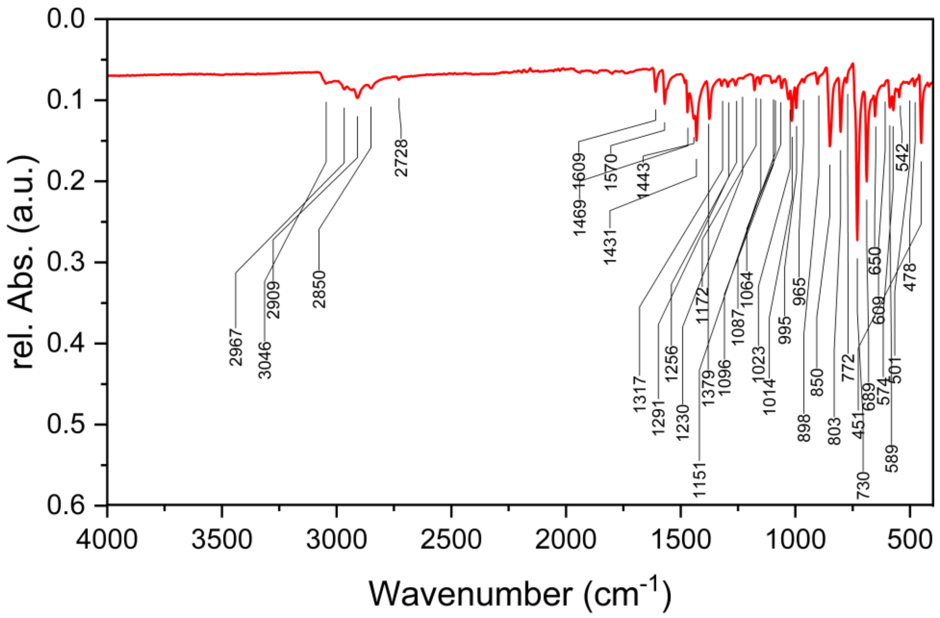

Figure S4. IR spectrum of DMPAs(TePh)$_2$ (**1**).

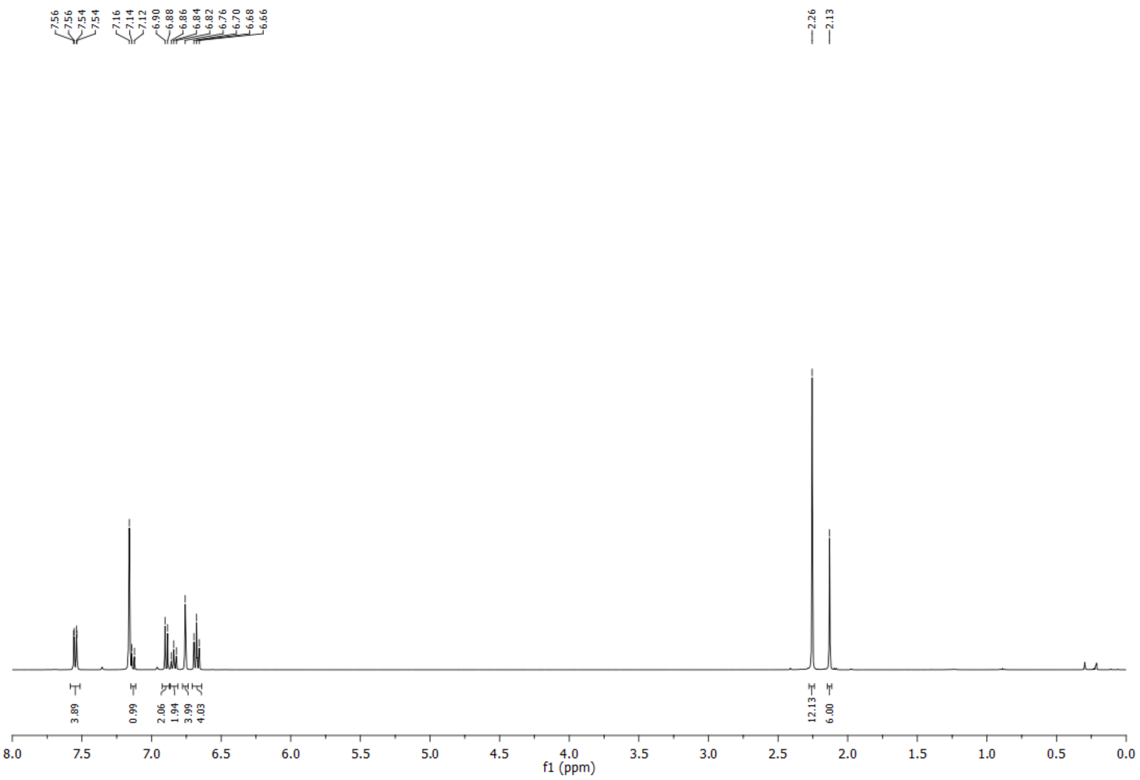

Figure S5. ^{1}H NMR spectrum of DMPSb(TePh)$_2$ (**2**) in benzene-d_6 at room temperature.

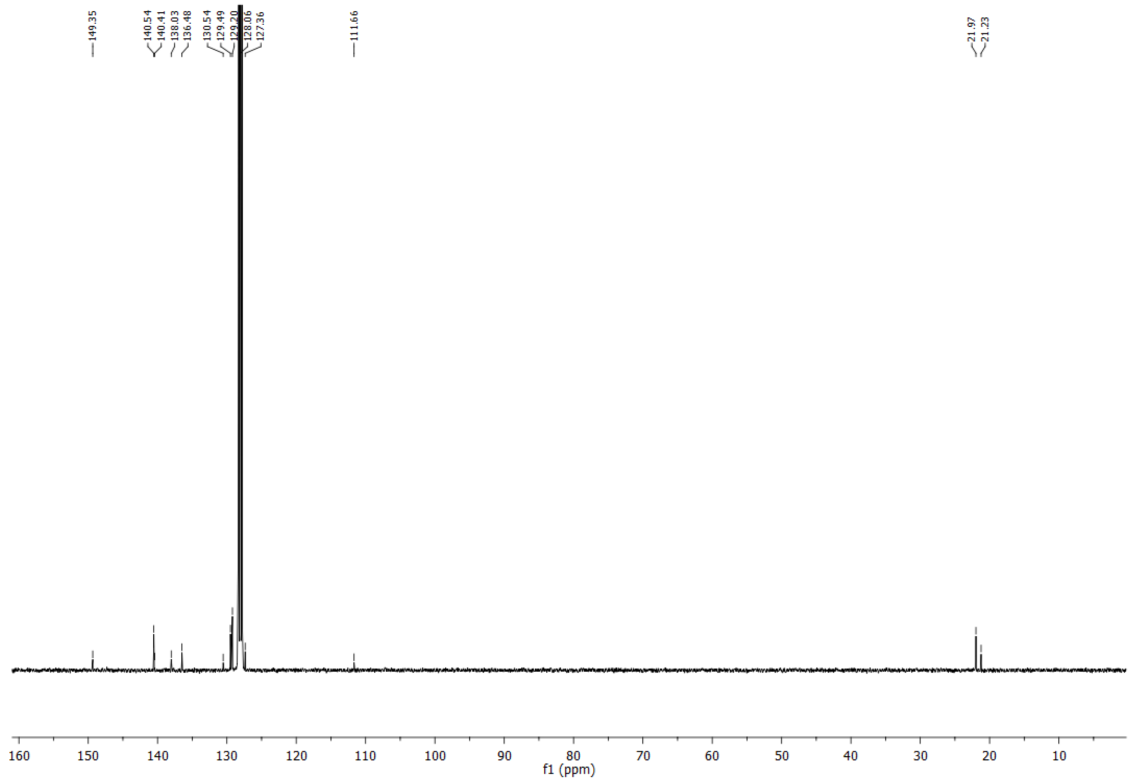

Figure S6. $^{13}C\{^{1}H\}$ NMR spectrum of DMPSb(TePh)$_2$ (**2**) in benzene-d_6 at room temperature.

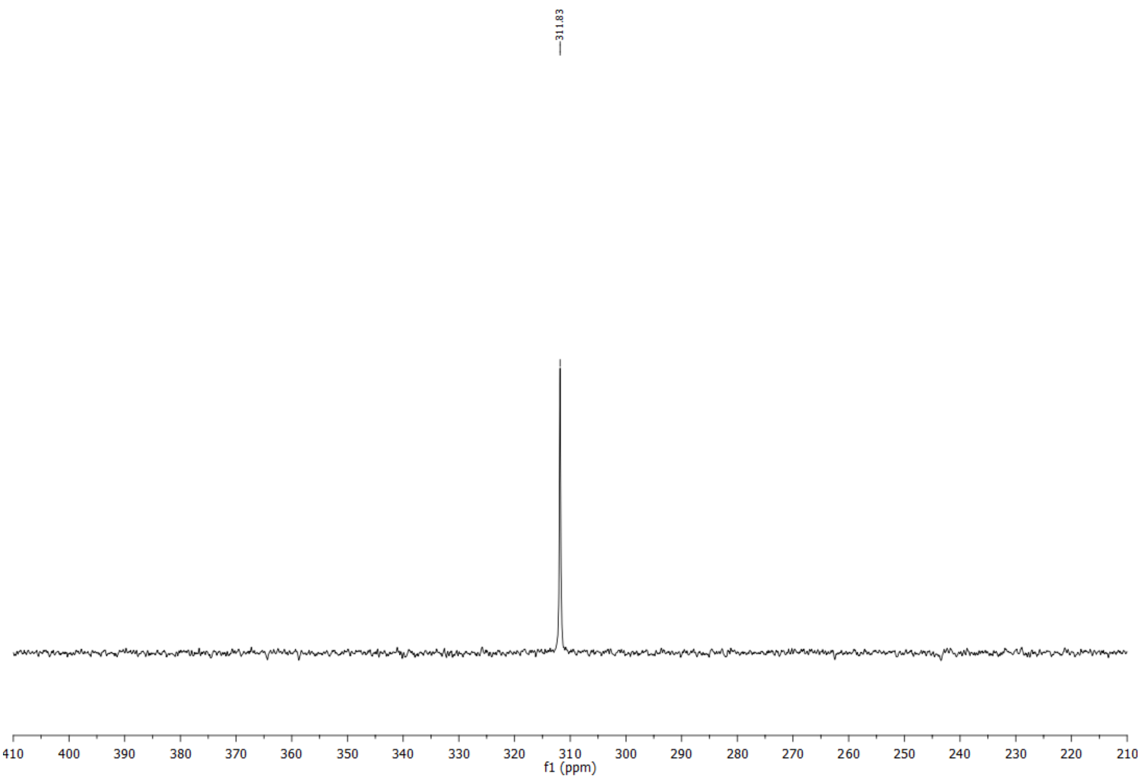

Figure S7. ^{125}Te NMR spectrum of DMPSb(TePh)$_2$ (**2**) in benzene-d_6 at room temperature.

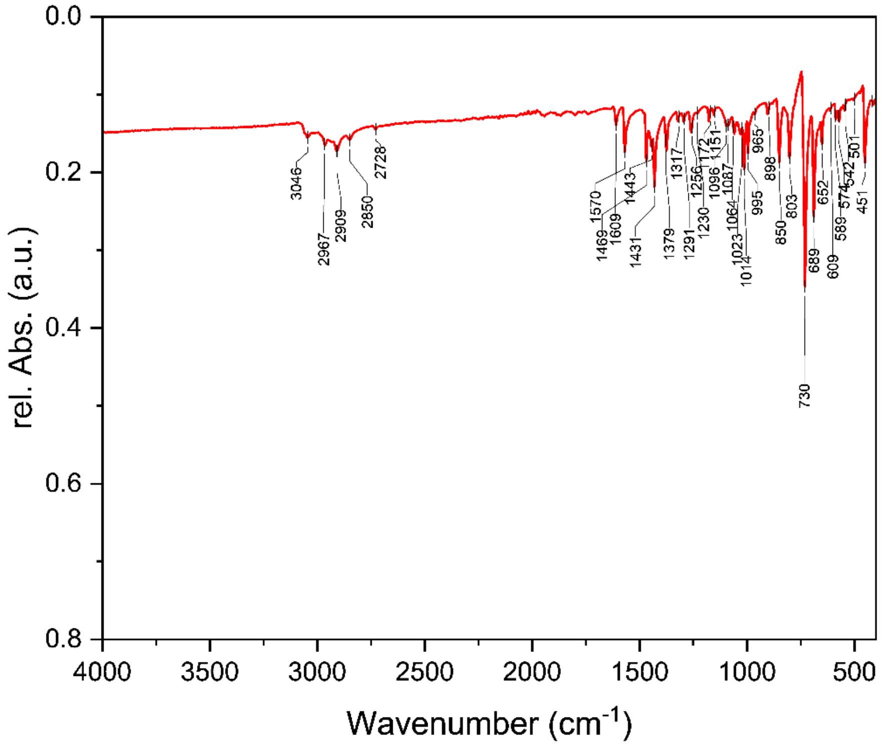

Figure S8. IR spectrum of DMPSb(TePh)$_2$ (**2**).

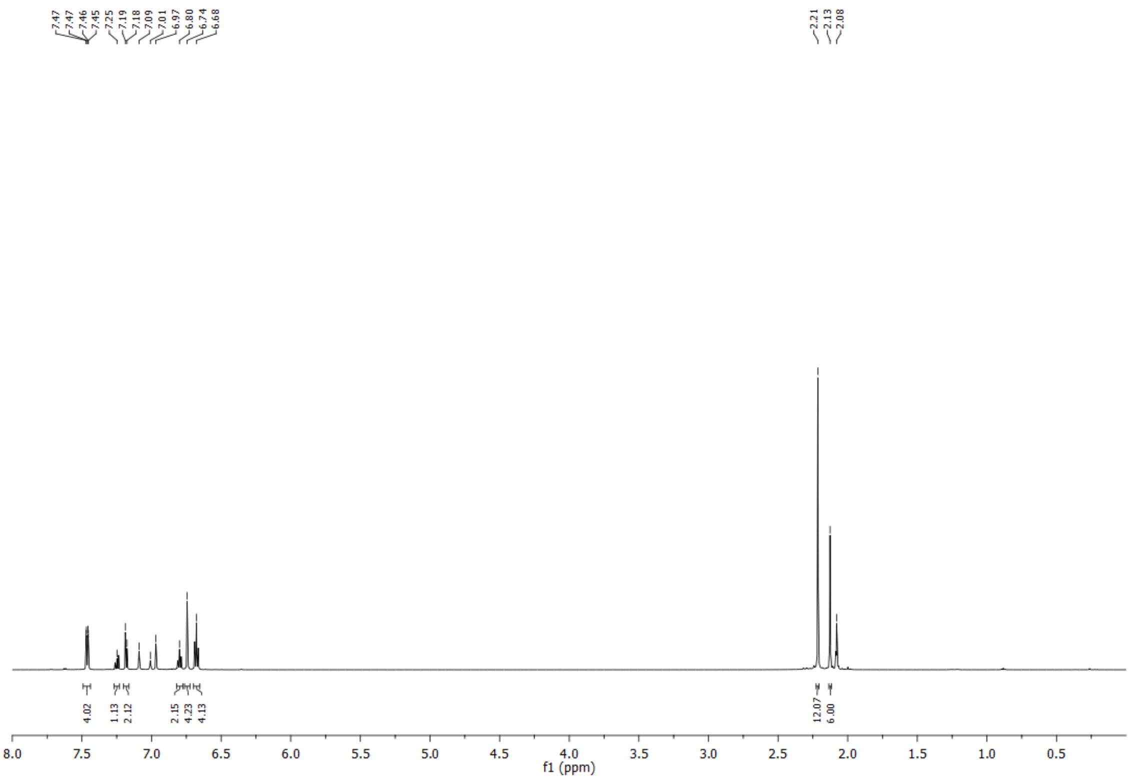

Figure S9. ^{1}H NMR spectrum of DMPBi(TePh)$_2$ (**3**) in toluene-d_8 at room temperature.

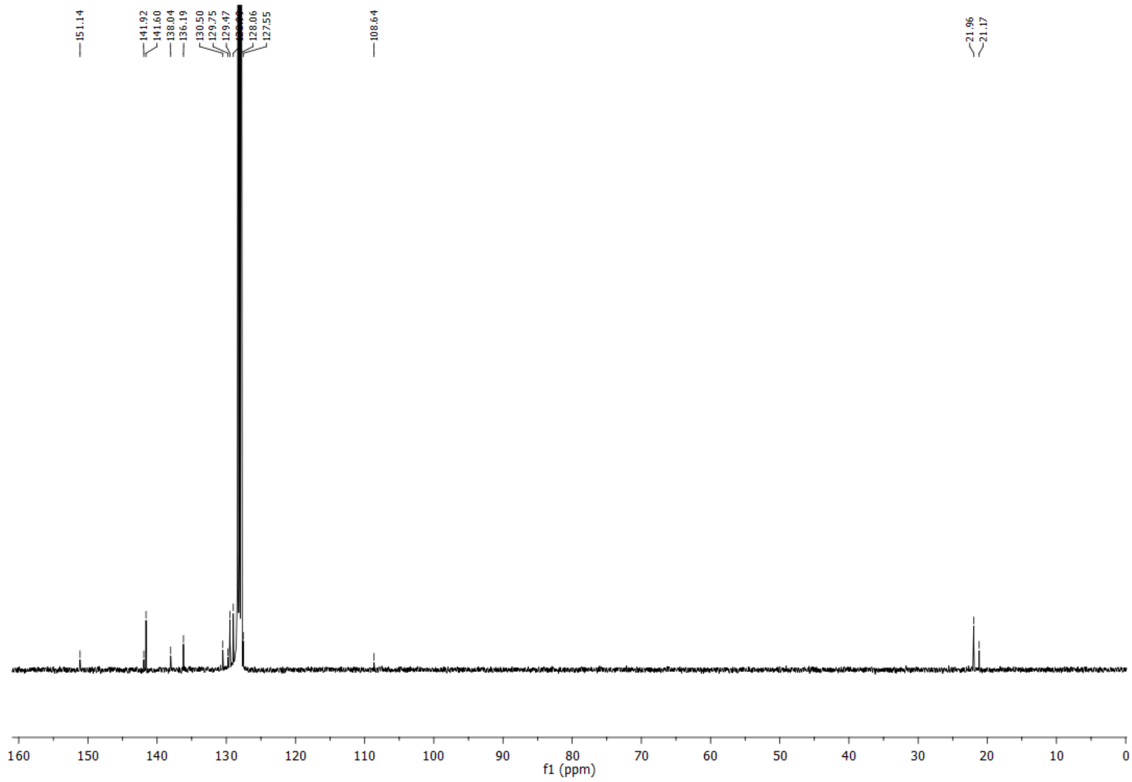

Figure S10. $^{13}C\{^{1}H\}$ NMR spectrum of DMPBi(TePh)$_2$ (**3**) in benzene-d_6 at room temperature.

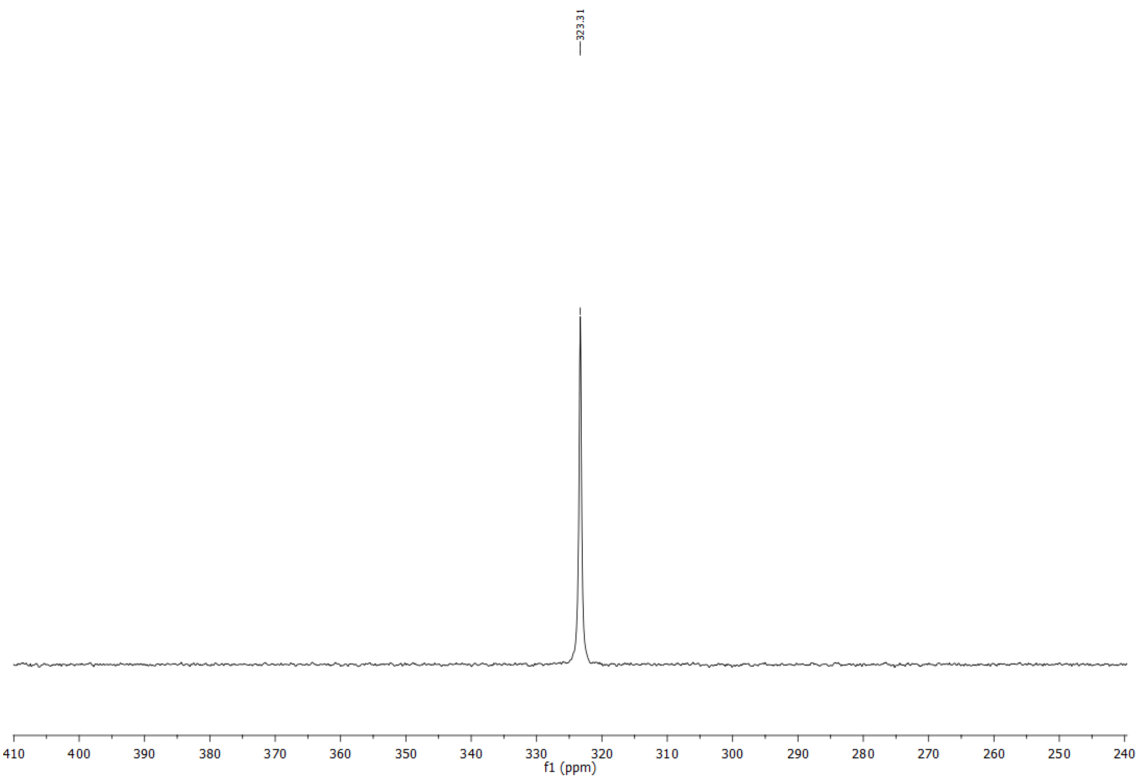

Figure S11. ^{125}Te NMR spectrum of DMPBi(TePh)$_2$ (**3**) in toluene-d_8 at room temperature.

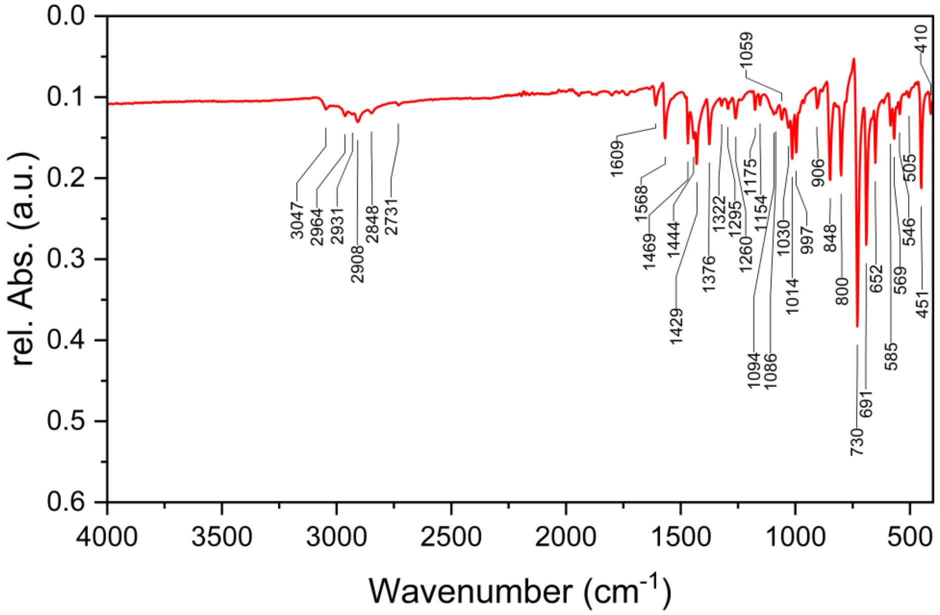

Figure S12. IR spectrum of DMPBi(TePh)$_2$ (**3**).

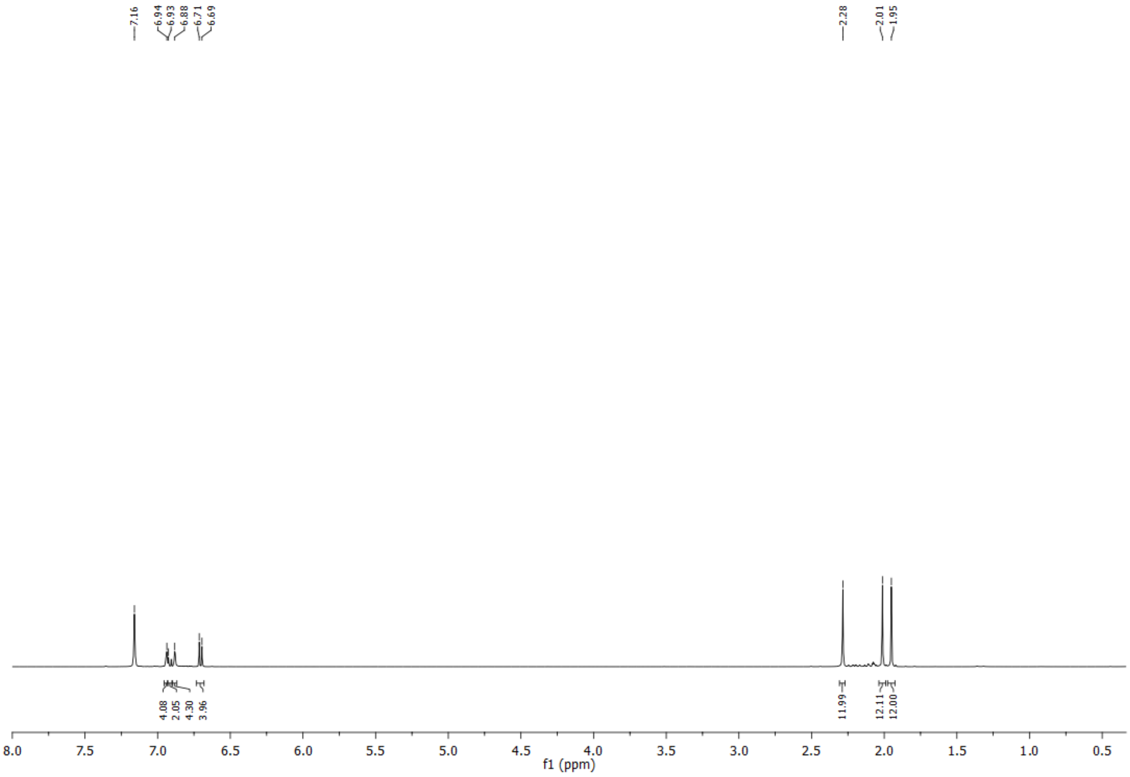

Figure S13. ^{1}H NMR spectrum of DMPAs(μ-Te)AsDMP (**4**) in benzene-d_6 at room temperature.

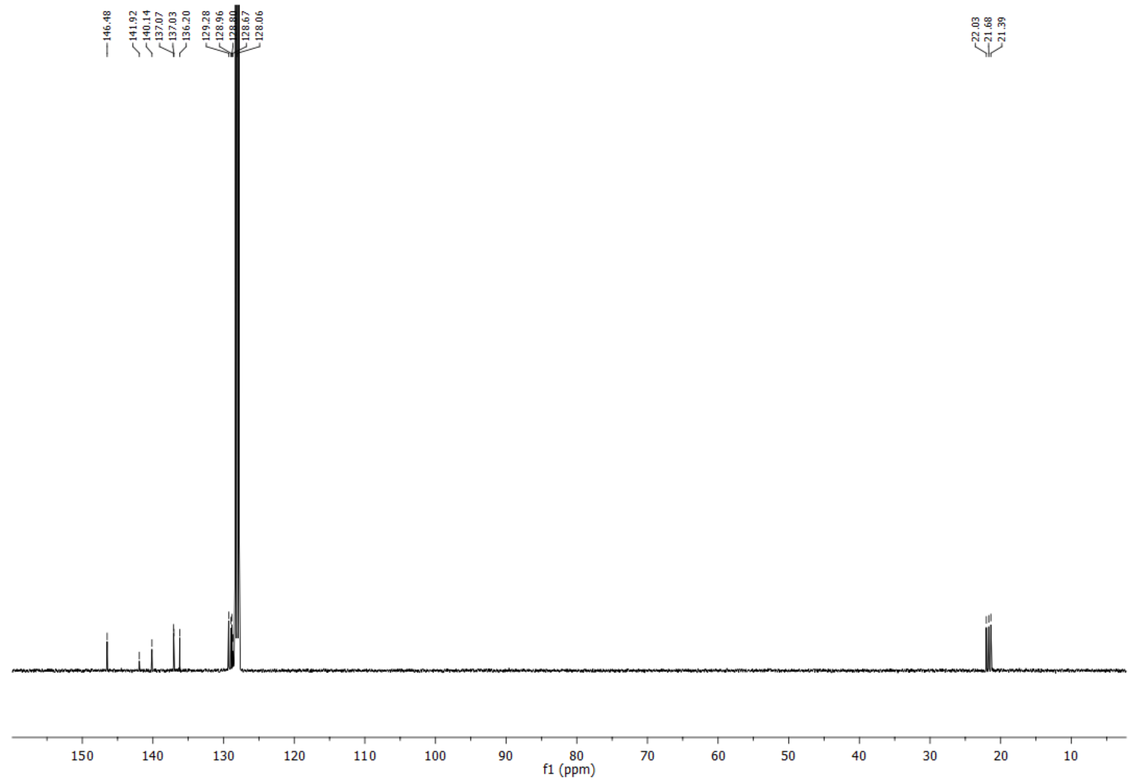

Figure S14. $^{13}C\{^{1}H\}$ NMR spectrum of DMPAs(μ-Te)AsDMP (**4**) in benzene-d_6 at room temperature.

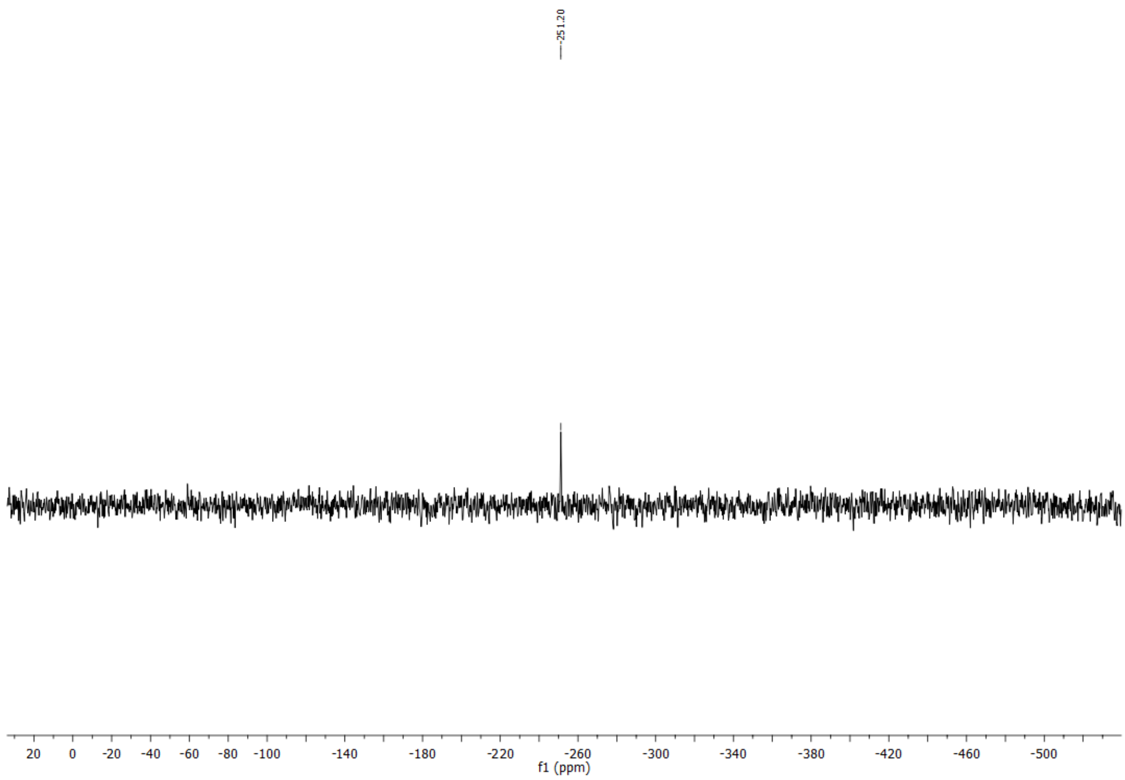

Figure S15. ^{125}Te NMR spectrum of DMPAs(μ-Te)AsDMP (**4**) in benzene-d_6 at room temperature.

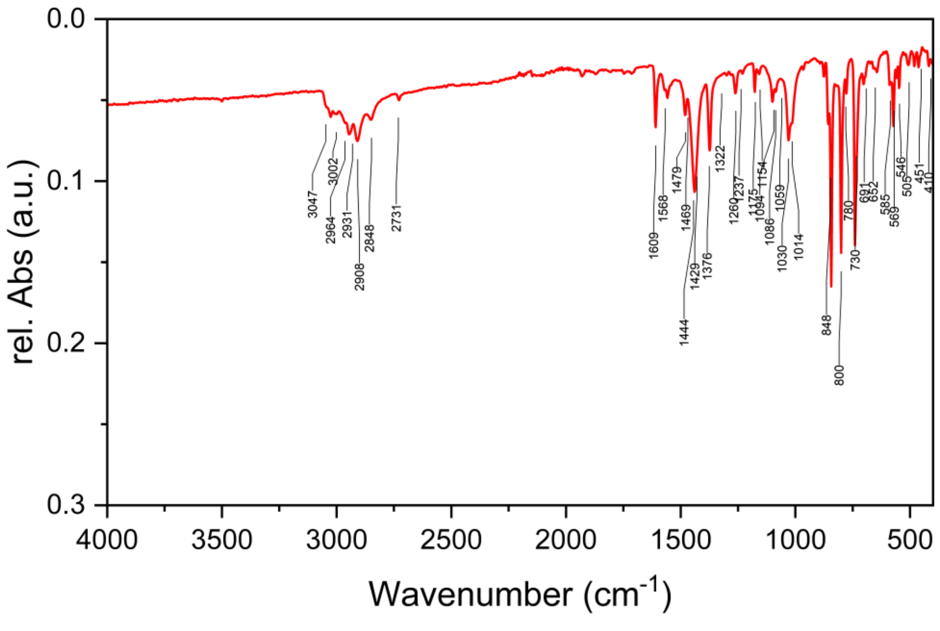

Figure S16. IR spectrum of DMPAs(μ-Te)AsDMP (**4**).

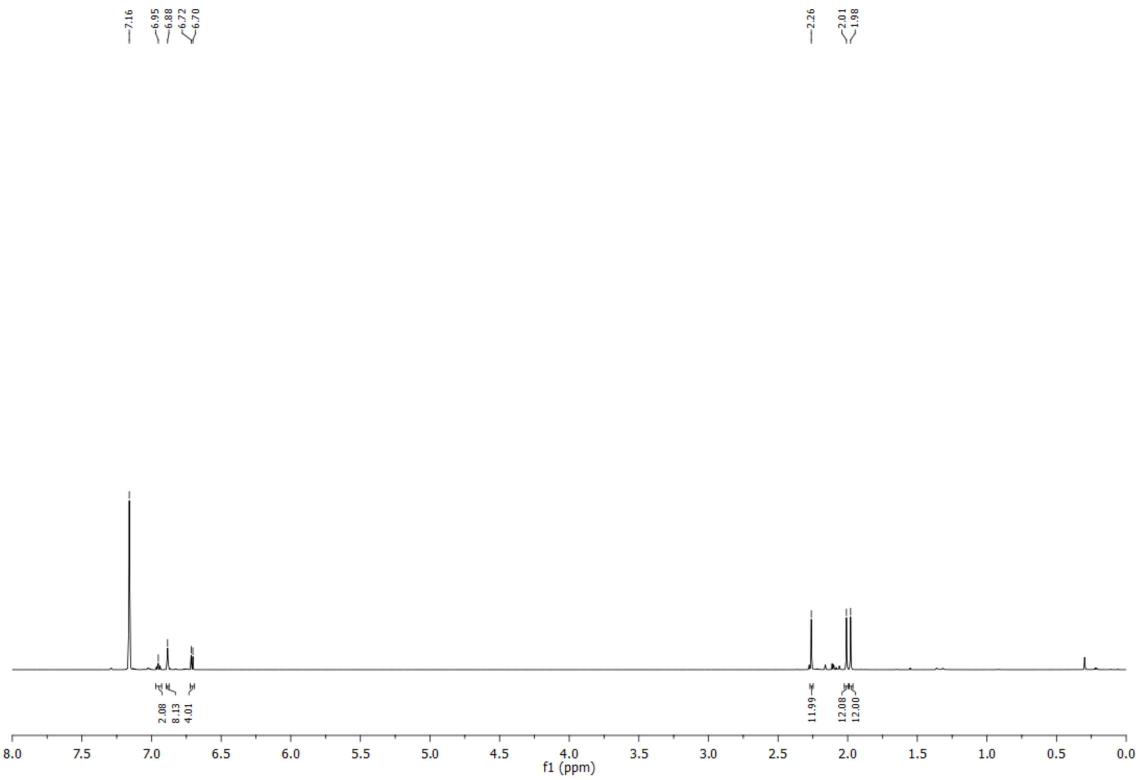

Figure S17. ^{1}H NMR spectrum of DMPSb(μ-Te)SbDMP (**5**) in benzene-d_6 at room temperature.

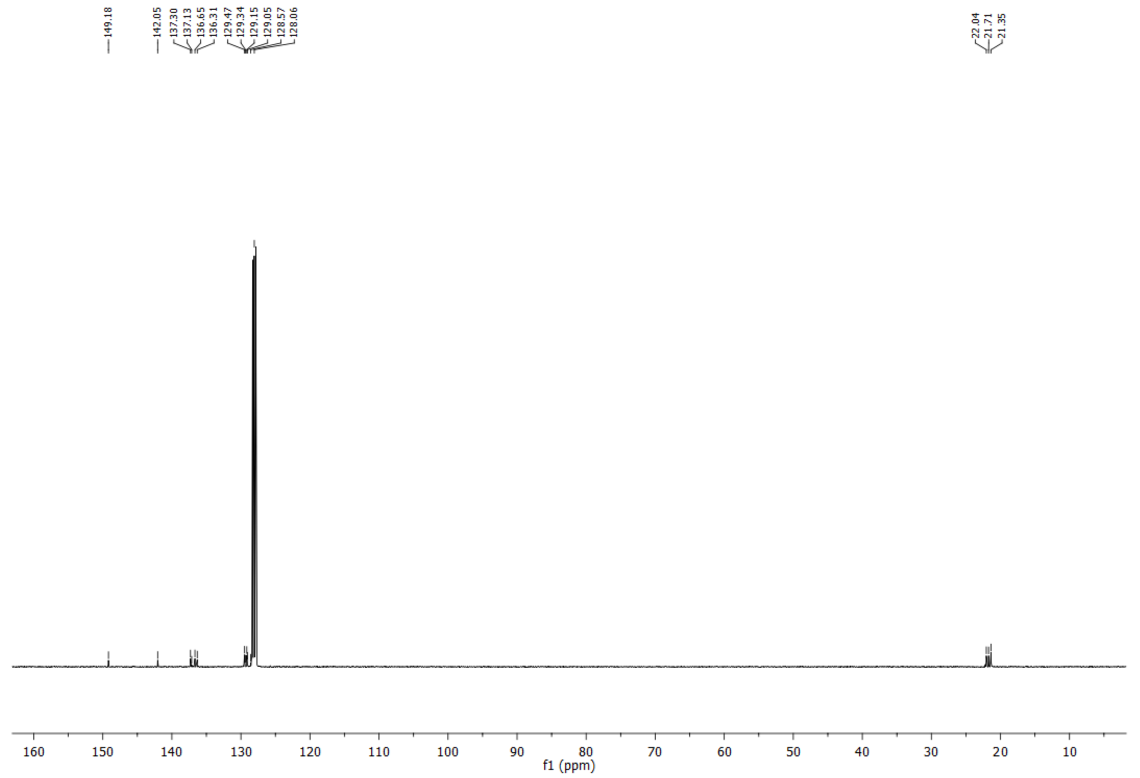

Figure S18. $^{13}C\{^{1}H\}$ NMR spectrum of DMPSb(μ-Te)SbDMP (**5**) in benzene-d_6 at room temperature.

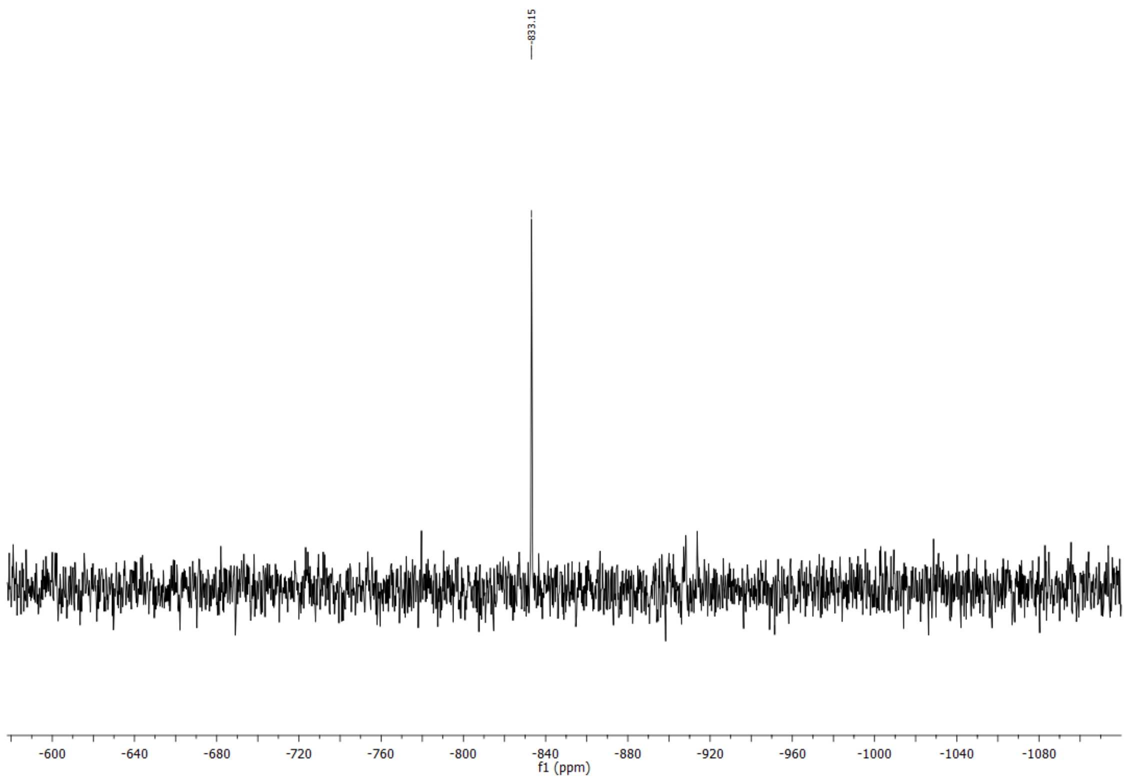

Figure S19. ^{125}Te NMR spectrum of DMPSb(μ-Te)SbDMP (**5**) in benzene-d_6 at room temperature.

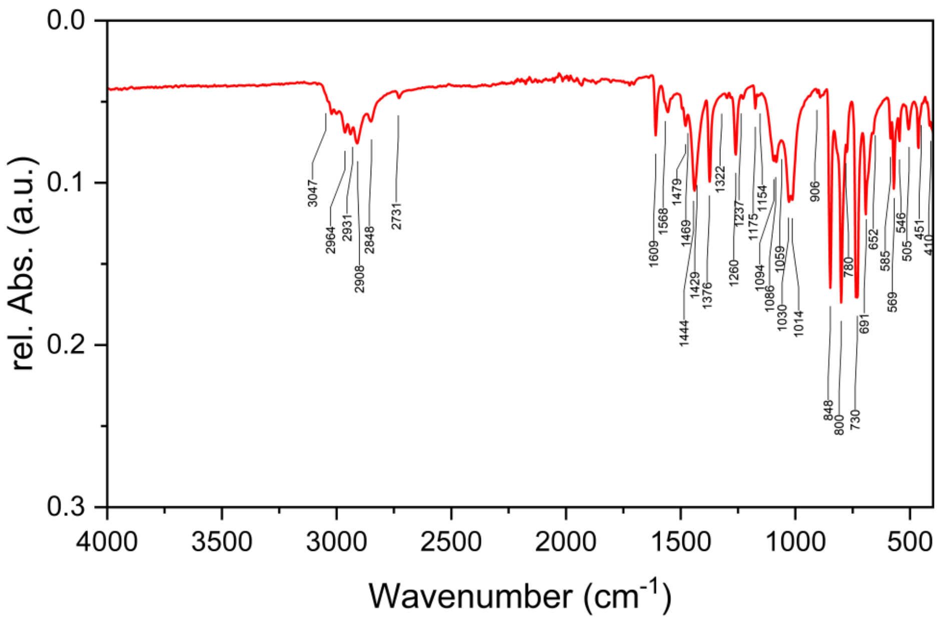

Figure S20. IR spectrum of DMPSb(μ-Te)SbDMP (**5**).

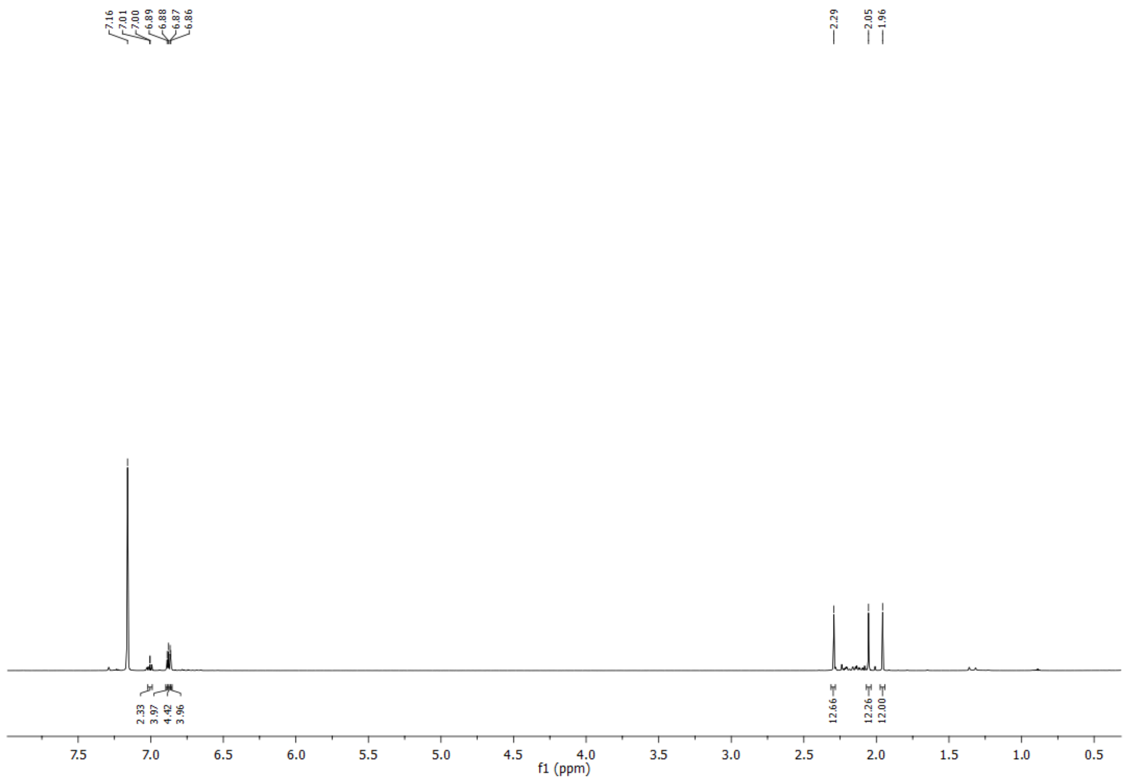

Figure S21. ^{1}H NMR spectrum of DMPBi(μ-Te)BiDMP (**6**) in benzene-d_6 at room temperature.

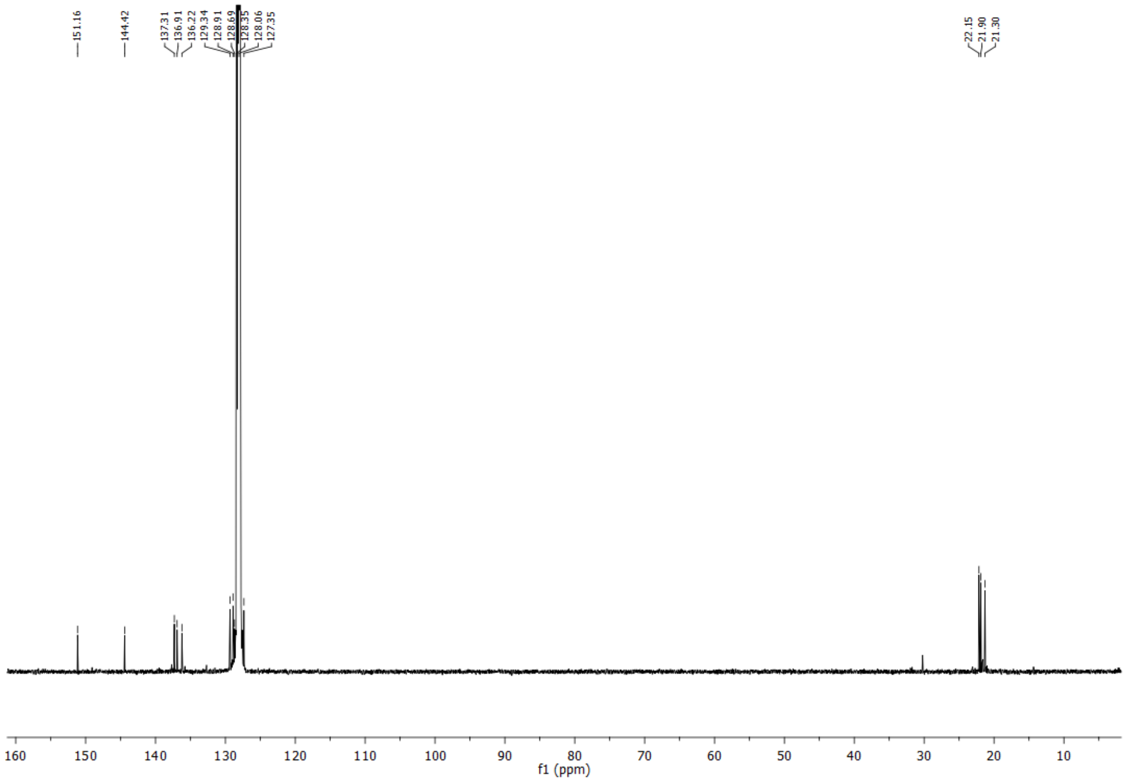

Figure S22. $^{13}C\{^{1}H\}$ NMR spectrum of DMPBi(μ-Te)BiDMP (**6**) in benzene-d_6 at room temperature.

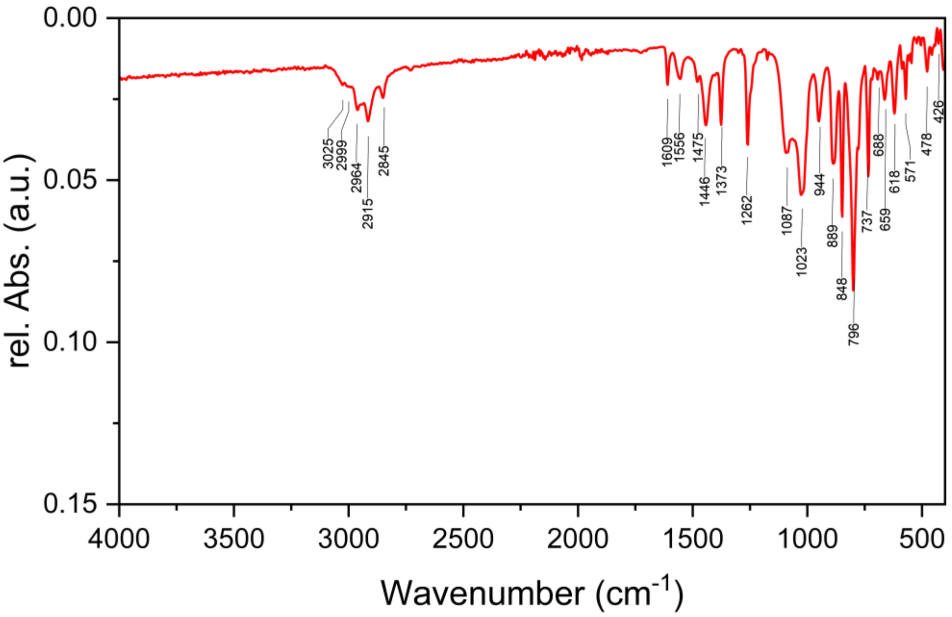

Figure S23. IR spectrum of DMPBi(μ-Te)BiDMP (**6**).

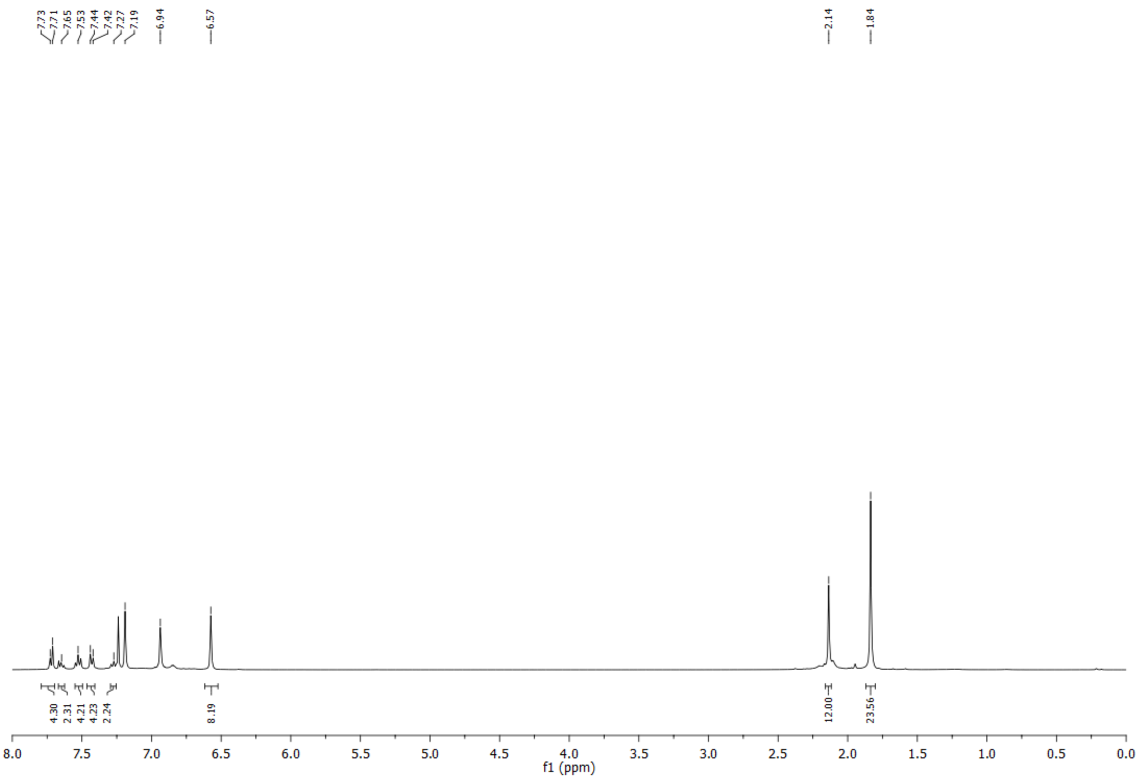

Figure S24. ^{1}H NMR spectrum of $[DMPBi(TePh)]_2[SbF_6]_2$ (**7**) in 1,2-dichlorobenzene-d_4 at room temperature.

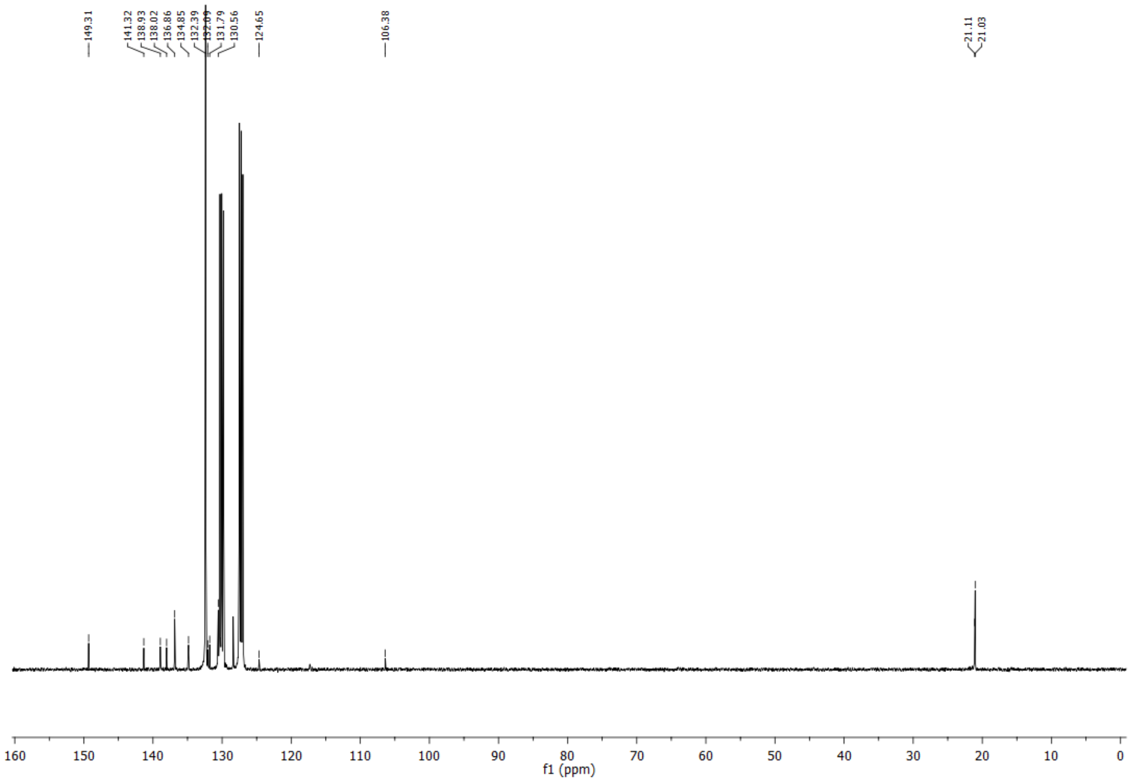

Figure S25. $^{13}C\{^{1}H\}$ NMR spectrum of $[DMPBi(TePh)]_2[SbF_6]_2$ (**7**) in 1,2-dichlorobenzene-d_4 at room temperature.

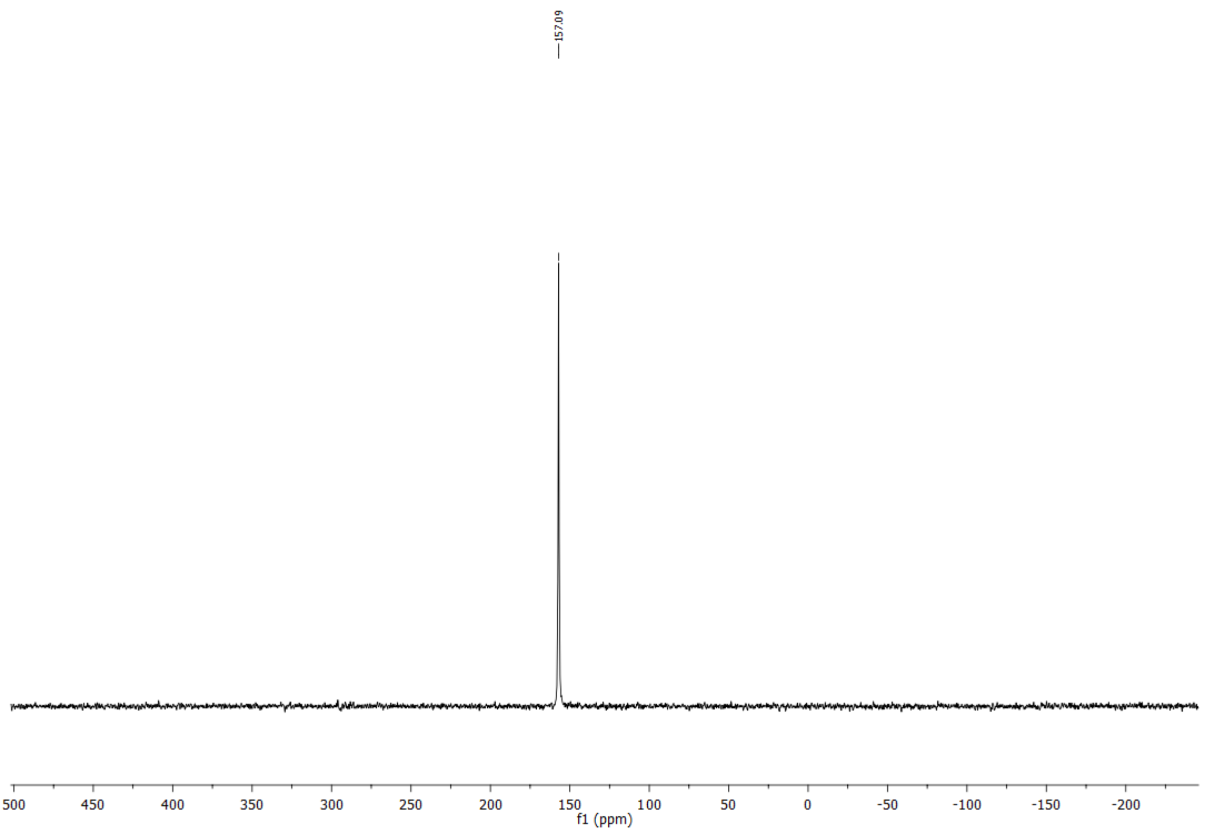

Figure S26. ^{125}Te NMR spectrum of $[DMPBi(TePh)]_2[SbF_6]_2$ (**7**) in 1,2-dichlorobenzene-d_4 at room temperature.

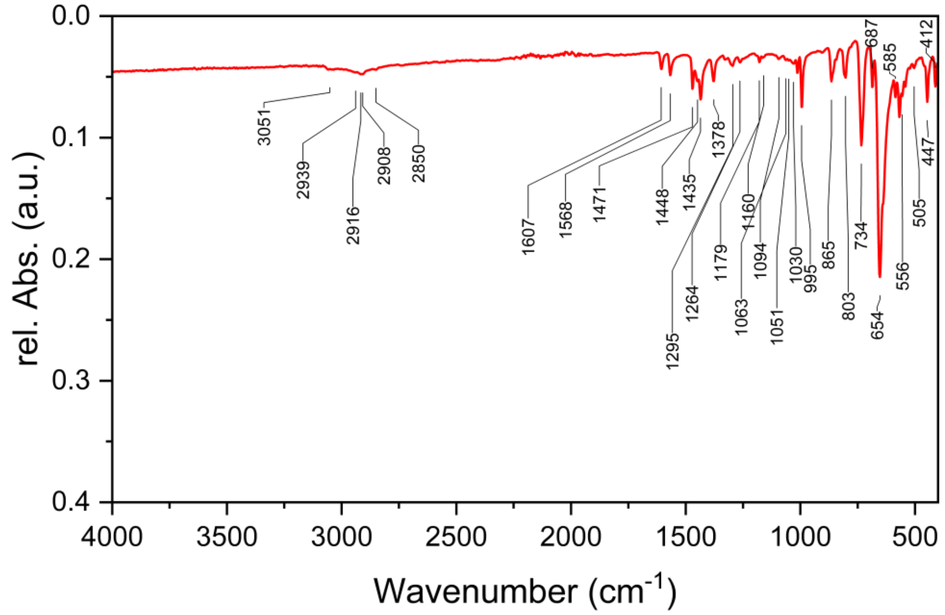

Figure S27. IR spectrum of $[DMPBi(TePh)]_2[SbF_6]_2$ (**7**).

II. Single Crystal X-ray Diffraction (sc-XRD)

The crystals were mounted on nylon loops in inert oil. Data of **1**, **2**, **4**, **5**, **6** and **7** were collected on a Bruker AXS D8 Venture diffractometer with Photon II detector (monochromated CuK_{α} radiation, λ = 1.54178 Å, microfocus source) at 100(2) or 200(2) K while those of **3** were collected on a Bruker AXS D8 Kappa diffractometer with APEX2 detector (monochromated MoK_{α} radiation, λ = 0.71073 Å) at 100(2) K. The structures were solved by Direct Methods (SHELXS-2013)[1] and refined anisotropically by fullmatrix least-squares on F^2 (SHELXL-2017).[2,3] Absorption corrections were performed semiempirically from equivalent reflections on basis of multi-scans and in case of **5** and **6** numerical form indexed faces (Bruker AXS APEX3). Hydrogen atoms were refined using a riding model or rigid methyl groups.

The structure of DMPAs$(TePh)_2$ (**1**) can be solved in monoclinic *C*2/*c* with *Z'* = 1 but the displacement parameters refine to unrealistic values and the *R*-values are significantly worse. At 100 K a change of colour to yellow and the formation of a superstructure was observed. The resulting unit cell is the alternate monoclinic cell with *a* and *b* doubled. Attempts to refine this structure yielded unrealistic values for the displacement parameters with several of them going to npd. This points to a possible modulation and the doubling of *a* and *b* being just an approximate description.

For DMPSb$(TePh)_2$ (**2**) at 100K a different phase is observed. Its colour is yellow and *a* and *b* of the unit cell are doubled. The displacement parameters refine to unrealistic values with very poor *R*-values. This suggests a possible modulation and the superstructure model to be nothing but a poor approximation.

The crystal of DMPBi$(TePh)_2$ (**3**) was a non-merohedral twin and structure was refined against de-twinned HKLF4 data. A phenyl ring is disordered over two positions. Its bond lengths and angles were restrained to be equal (SADI) and RIGU and SIMU restraints were applied to its displacement parameters. The atoms of the group were restrained to lie on a common plane (FLAT).

In DMPSb(µ-Te)SbDMP (**5**) the toluene molecule is disordered over a centre of inversion. The bond lengths and angles of its phenyl ring were restrained to be equal (SADI) and the local symmetry was ignored in the refinement (negative PART).

Table S1. Crystal data and structure refinement of compounds **1**, **2** and **3**.

Compound	DMPAs(TePh)$_2$ (1)	DMPSb(TePh)$_2$ (2)	DMPBi(TePh)$_2$ (3)
Emp. formula	$C_{36}H_{35}AsTe_2$	$C_{36}H_{35}SbTe_2$	$C_{36}H_{35}BiTe_2$
Formula weight [Da]	797.76	844.59	931.82
Temperature [K]	200(2)	200(2)	100(2)
Crystal system	triclinic	monoclinic	monoclinic
Space group	$P\bar{1}$	$C2/c$	$C2/c$
a [Å]	11.9529(4)	21.0898(5)	20.964(4)
b [Å]	19.5673(6)	11.1489(4)	11.074(2)
c [Å]	28.2861(9)	29.0540(12)	28.781(6)
α [°]	96.9250(10)	90	90
β [°]	98.6414(9)	105.9077(18)	105.548(4)
γ [°]	95.1077(9)	90	90
V [Å^3]	6454.1(4)	6572.5(4)	6437(2)
Z	8	8	8
Density (calc.) [g·cm^{-3}]	1.642	1.707	1.923
μ [mm^{-1}]	15.562	20.541	7.277
F (000)	3104	3248	3504
Crystal size [mm^3]	0.320 × 0.228 × 0.211	0.274 × 0.170 × 0.107	0.149 × 0.047 × 0.034
ϑ max [°]	67.679	67.679	25.242
Index ranges	$-15 \le h \le 15$<br>$-24 \le k \le 24$<br>$-36 \le l \le 35$	$-26 \le h \le 26$<br>$-13 \le k \le 14$<br>$-36 \le l \le 36$	$-28 \le h \le 27$<br>$0 \le k \le 14$<br>$0 \le l \le 38$
Reflections collected	30448	15032	8080
Independent reflections	27702	7108	8080
R_{int}	0.0666	0.0637	0.0930
Data/restraints/parameters	27702 / 0 / 1429	7108 / 0 / 358	8080 / 390 / 413
Goodness-of-fit on F^2	1.037	1.098	1.476
R_1 [$I > 2\sigma(I)$]	0.0375	0.0409	0.0673
wR_2 [$I > 2\sigma(I)$]	0.1040	0.1133	0.1166
R_1 (all data)	0.0391	0.0443	0.0871
wR_2 (all data)	0.1057	0.1175	0.1204
Largest diff. peak/hole [e·Å^{-3}]	1.359 / −1.536	1.576 / −0.703	1.645 / −2.136

Table S2. Crystal data and structure refinement of compounds **4**, **5** and **6**.

**Compound**	**DMPAs(μTe)AsDMP (4)**	**DMPSb(μTe)SbDMP (5)**	**DMPBi(μTe)BiDMP (6)**
Emp. formula	$C_{48}H_{50}As_2Te$	$C_{51.50}H_{54}Sb_2Te$	$C_{51}H_{53}Bi_2Te$
Formula weight [Da]	904.32	1044.04	1211.49
Temperature [K]	100(2)	100(2)	100(2)
Crystal system	monoclinic	monoclinic	monoclinic
Space group	$P2_1/n$	$P2_1/c$	$P2_1/c$
a [Å]	12.9665(5)	14.1262(5)	14.2932(7)
b [Å]	19.9151(7)	19.3526(7)	19.3058(10)
c [Å]	16.8682(6)	17.6493(6)	17.5967(9)
α [°]	90	90	90
β [°]	111.0878(11)	112.2555(16)	113.038(2)
γ [°]	90	90	90
V [Å^3]	4064.1(3)	4465.5(3)	4468.4(4)
Z	4	4	4
Density (calc.) [g·cm^{-3}]	1.478	1.553	1.801
μ [mm^{-1}]	7.817	14.884	20.564
F (000)	1824	2068	2308
Crystal size [mm^3]	0.193 × 0.131 × 0.034	0.309 × 0.110 × 0.018	0.281 × 0.080 × 0.037
ϑ max [°]	79.575	79.371	80.740
Index ranges	$-16 \le h \le 16$<br>$-25 \le k \le 24$<br>$-21 \le l \le 21$	$-17 \le h \le 17$<br>$-24 \le k \le 24$<br>$-19 \le l \le 22$	$-18 \le h \le 18$<br>$-24 \le k \le 24$<br>$-22 \le l \le 19$
Reflections collected	159006	227750	183595
Independent reflections	8816	9627	9762
R_{int}	0.0613	0.0809	0.0665
Data/restraints/parameters	8816 / 0 / 472	9627 / 30 / 536	9762 / 0 / 499
Goodness-of-fit on F^2	1.049	1.068	1.035
R_1 [$I > 2\sigma(I)$]	0.0283	0.0301	0.0220
wR_2 [$I > 2\sigma(I)$]	0.0763	0.0716	0.0540
R_1 (all data)	0.0298	0.0366	0.0246
wR_2 (all data)	0.0777	0.0772	0.0560
Largest diff. peak/hole [e·Å^{-3}]	1.536 / –0.900	1.613 / –0.667	0.774 / –1.236

Table S3. Crystal data and structure refinement of compound **7**.

Compound	$[DMPBi(TePh)]_2[SbF_6]_2$ (7)
Emp. formula	C_{72} $H_{70.92}$ Bi_2 $F_{13.08}$ Sb_2 Te_2
Formula weight [Da]	2101.47
Temperature [K]	100(2)
Crystal system	triclinic
Space group	$P\bar{1}$
a [Å]	12.3004(8)
b [Å]	13.4246(8)
c [Å]	22.7656(15)
α [°]	74.823(3)
β [°]	81.902(3)
γ [°]	77.265(3)
V [Å^3]	3525.0(4)
Z	2
Density (calc.) [g·cm^{-3}]	1.980
μ [mm^{-1}]	22.674
F (000)	1985
Crystal size [mm^3]	0.146 × 0.128 × 0.062
ϑ max [°]	81.029
Index ranges	$-14 \leq h \leq 15$<br>$-17 \leq k \leq 17$<br>$-29 \leq l \leq 28$
Reflections collected	197805
Independent reflections	15285
R_{int}	0.0663
Data/restraints/parameters	15285 / 1236 / 1096
Goodness-of-fit on F^2	1.041
R_1 [$I > 2\sigma(I)$]	0.0354
wR_2 [$I > 2\sigma(I)$]	0.0848
R_1 (all data)	0.0409
wR_2 (all data)	0.0896
Largest diff. peak/hole [e·Å^{-3}]	2.602 / −2.103

III. Cyclic Voltammetry (CV)

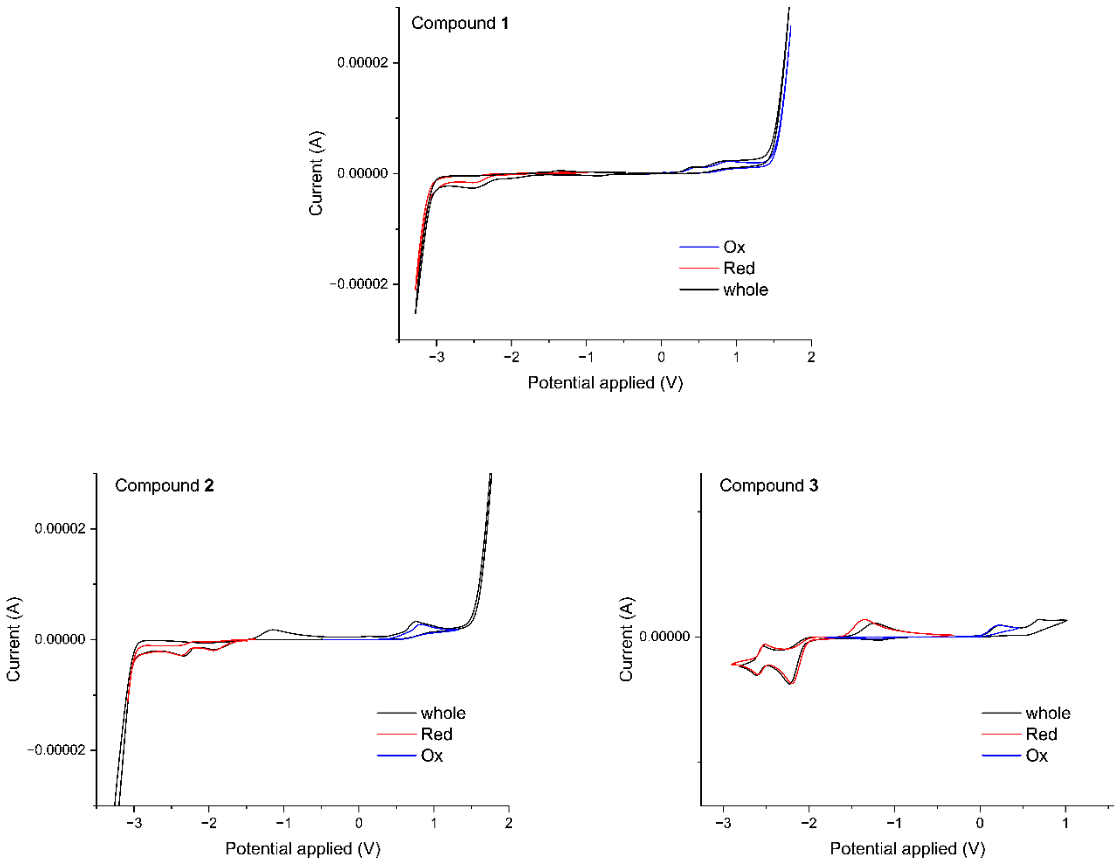

Figure S28. Cyclic voltammograms of **1**-**3** in THF (**1**, **2**) or 1,2-difluorobenzene (**3**) (0.01 M *n*-$Bu_4N[B(C_6F_5)_4]$ as a supporting electrolyte, 0.1 Vs^{-1}, vs Fc/Fc^+). The cycle with Fc/Fc^+ couple has been removed for clarity.

IX. Decomposition studies

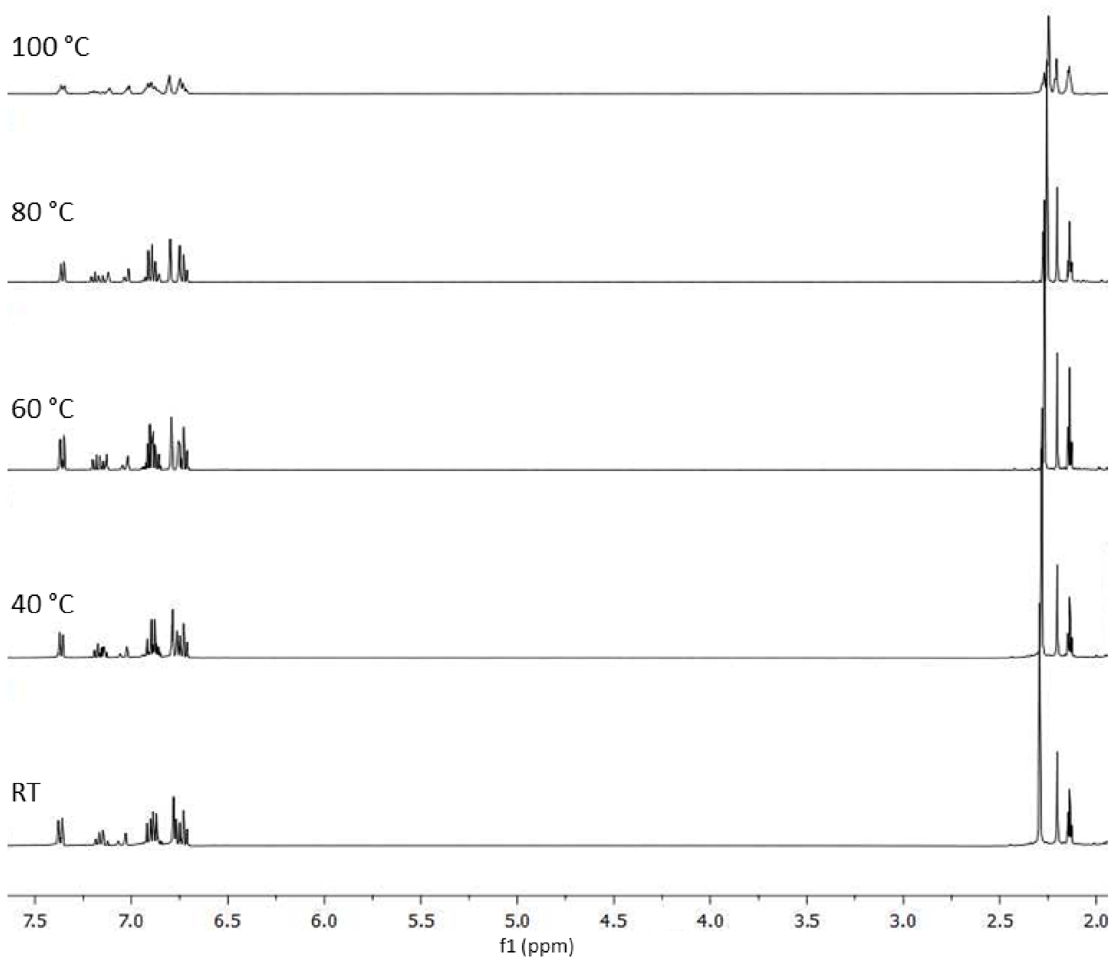

Figure S29. Temperature-dependent *in situ* ^{1}H NMR spectra of compound **1** in toluene-d_8. Each temperature was hold for 30 min before measurement.

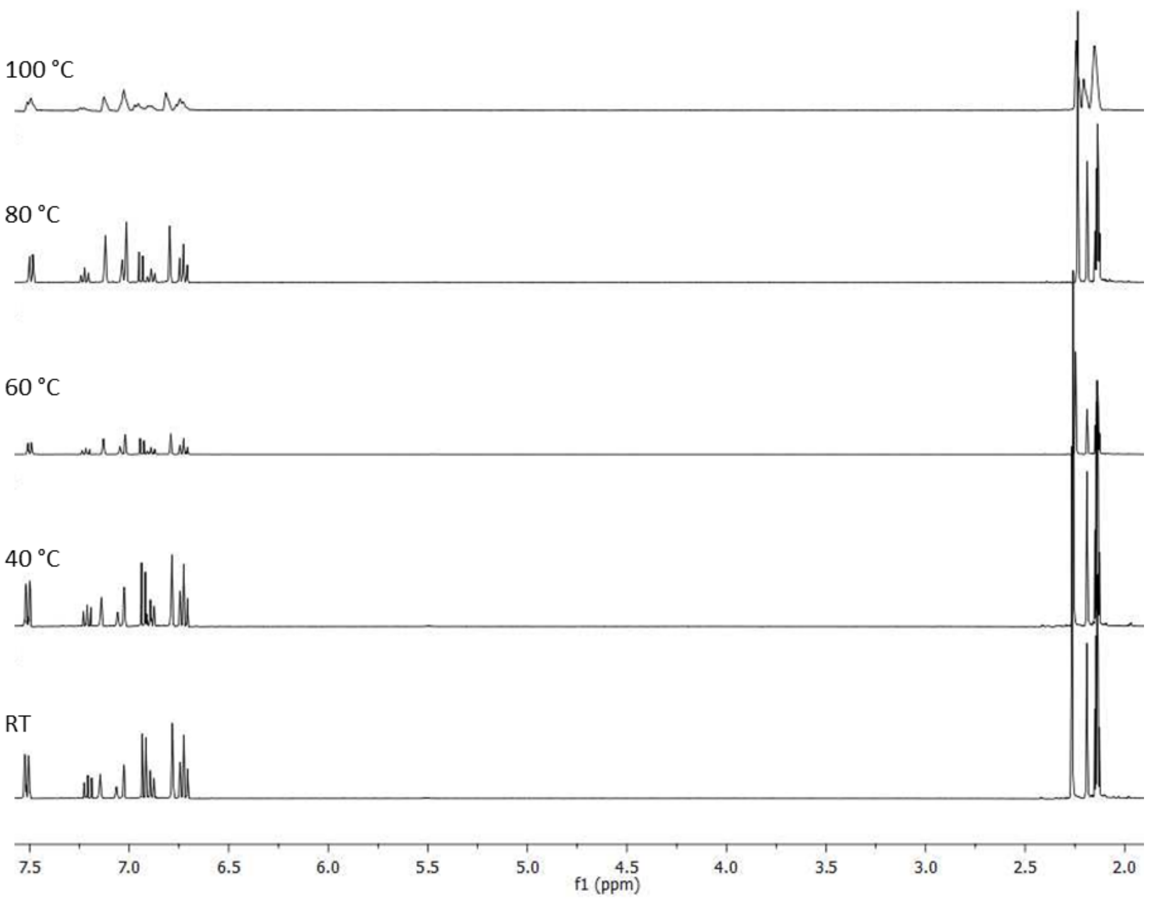

Figure S30. Temperature-dependent *in situ* ^{1}H NMR spectra of compound **2** in toluene-d_8. Each temperature was hold for 30 min before measurement.

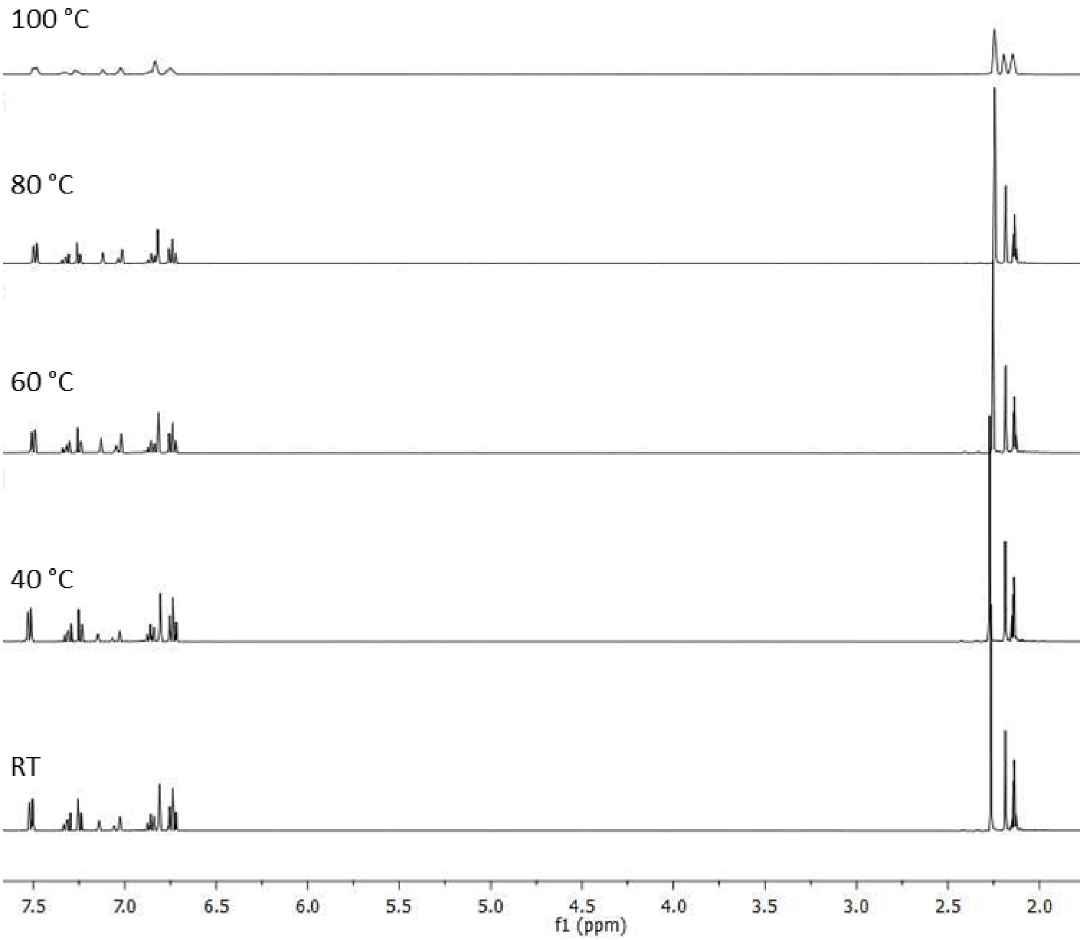

Figure S31. Temperature-dependent *in situ* ^{1}H NMR spectra of compound **3** in toluene-d_8. Each temperature was hold for 30 min before measurement.

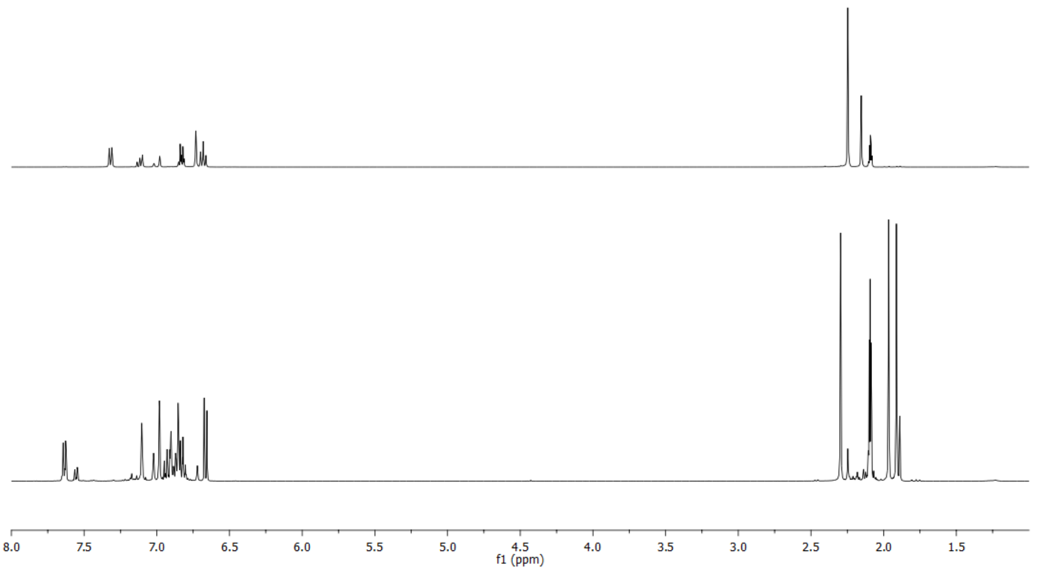

Figure S32. ^{1}H NMR spectra of compound **1** in toluene-d_8 measured at room temperature before (top) and after UV irradiation (bottom).

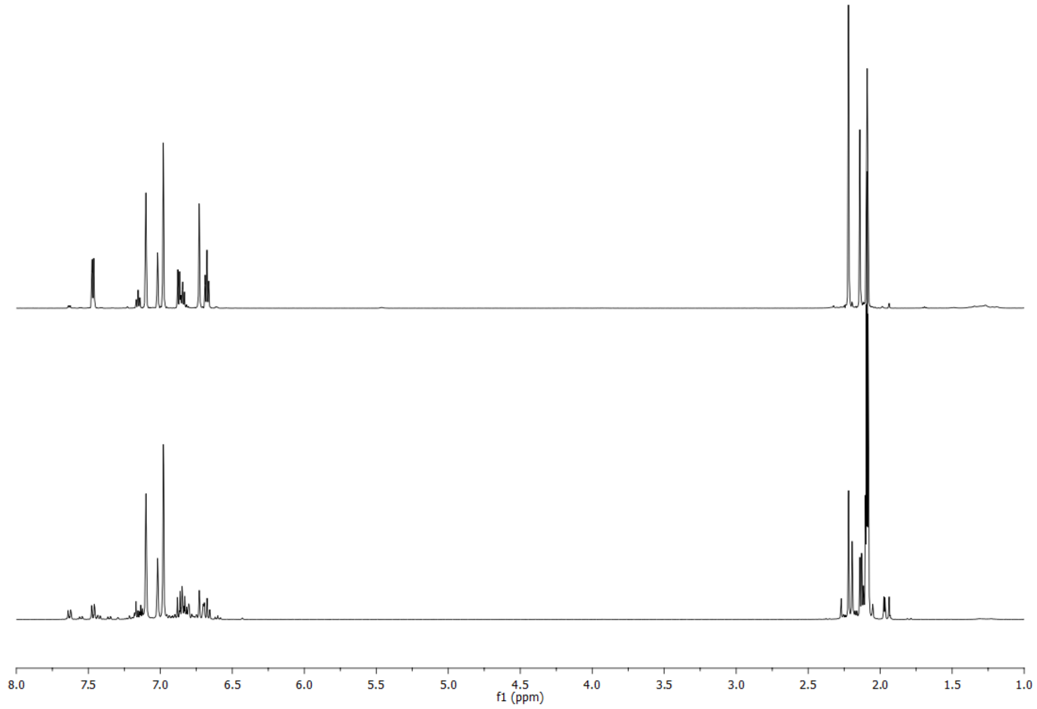

Figure S33. ^{1}H NMR spectra of compound **2** in toluene-d_8 measured at room temperature before (top) and after UV irradiation (bottom).

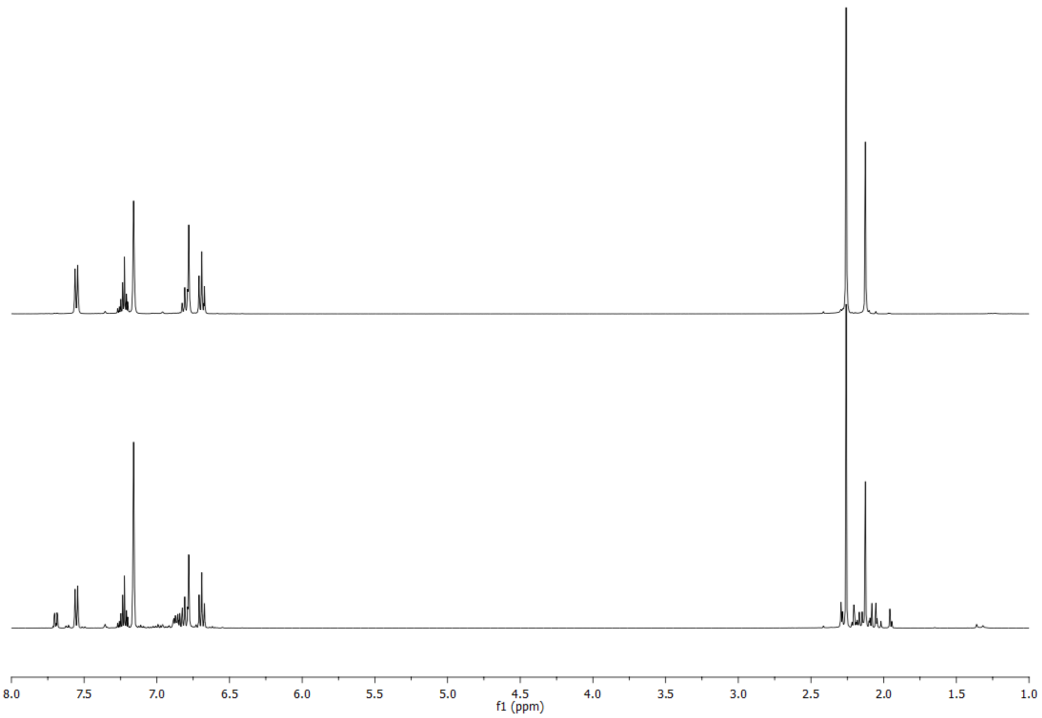

Figure S34. ^{1}H NMR spectra of compound **3** in benzene-d_6 measured at room temperature before (top) and after UV irradiation (bottom).

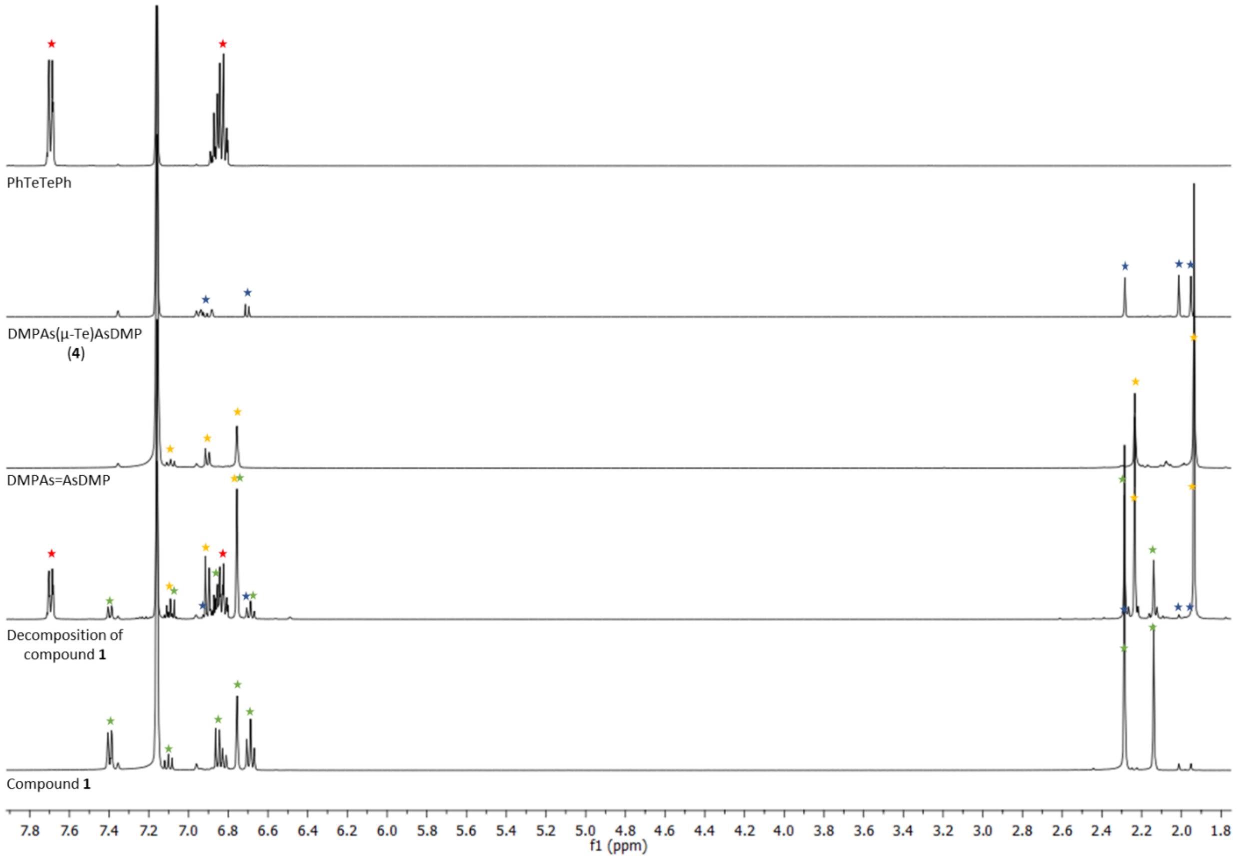

Figure S35. Comparison of ^{1}H NMR spectrum of compound **1** with its crude decomposition ^{1}H NMR spectrum together with ^{1}H NMR spectra of successfully identified and isolated decomposition products. All spectra are measured in benzene-d_6 at room temperature.

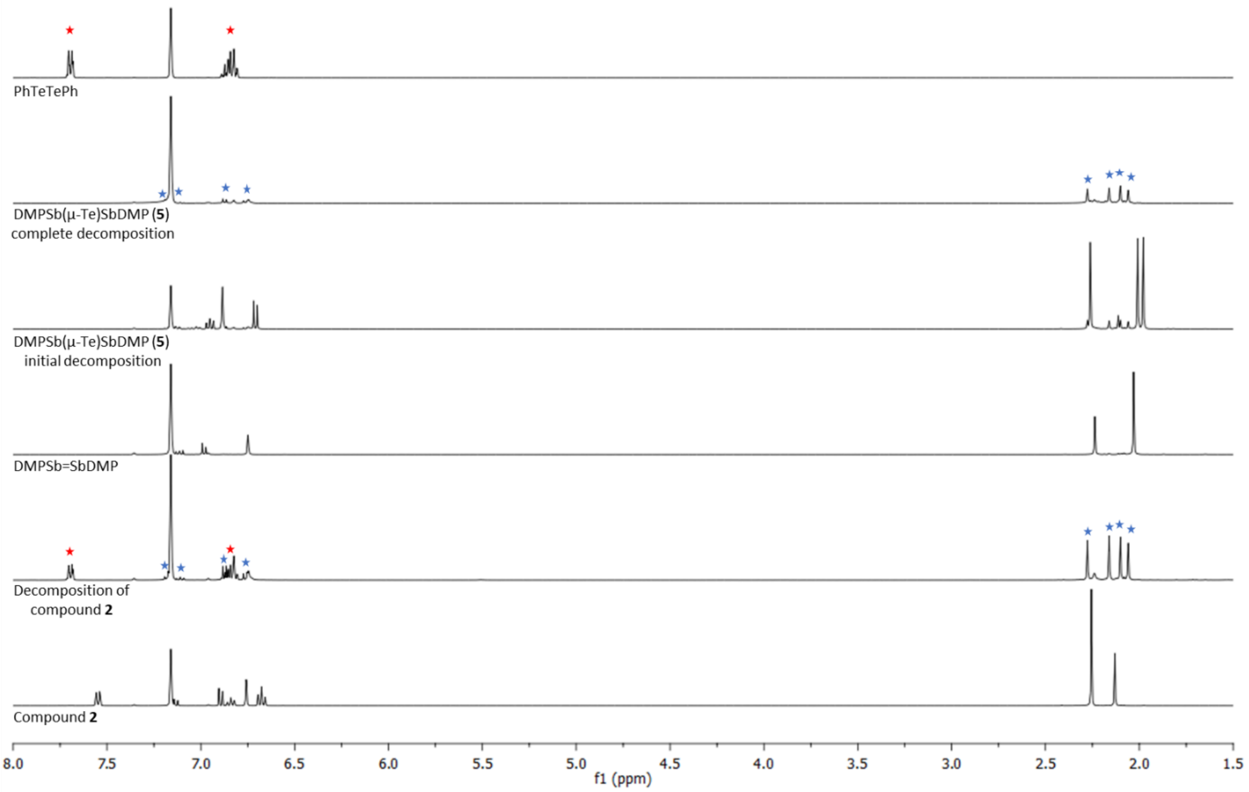

Figure S36. Comparison of ^{1}H NMR spectrum of compound **2** with its crude decomposition ^{1}H NMR spectrum together with ^{1}H NMR spectra of successfully identified diphenylditelluride and other decomposition products. All spectra are measured in benzene-d_6 at room temperature.

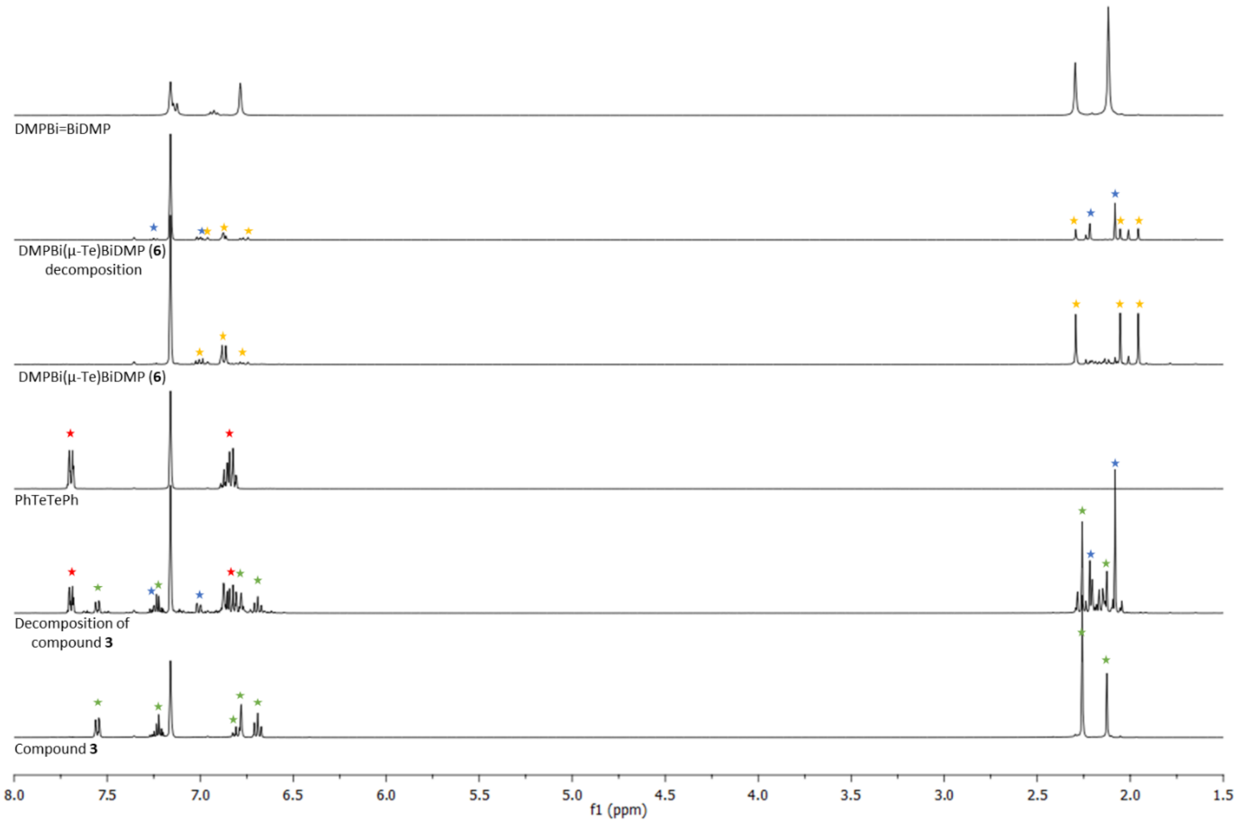

Figure S37. Comparison of ^{1}H NMR spectrum of compound **3** with its crude decomposition ^{1}H NMR spectrum together with ^{1}H NMR spectra of successfully identified diphenylditelluride and other decomposition products. All spectra are measured in benzene-d_6 at room temperature.

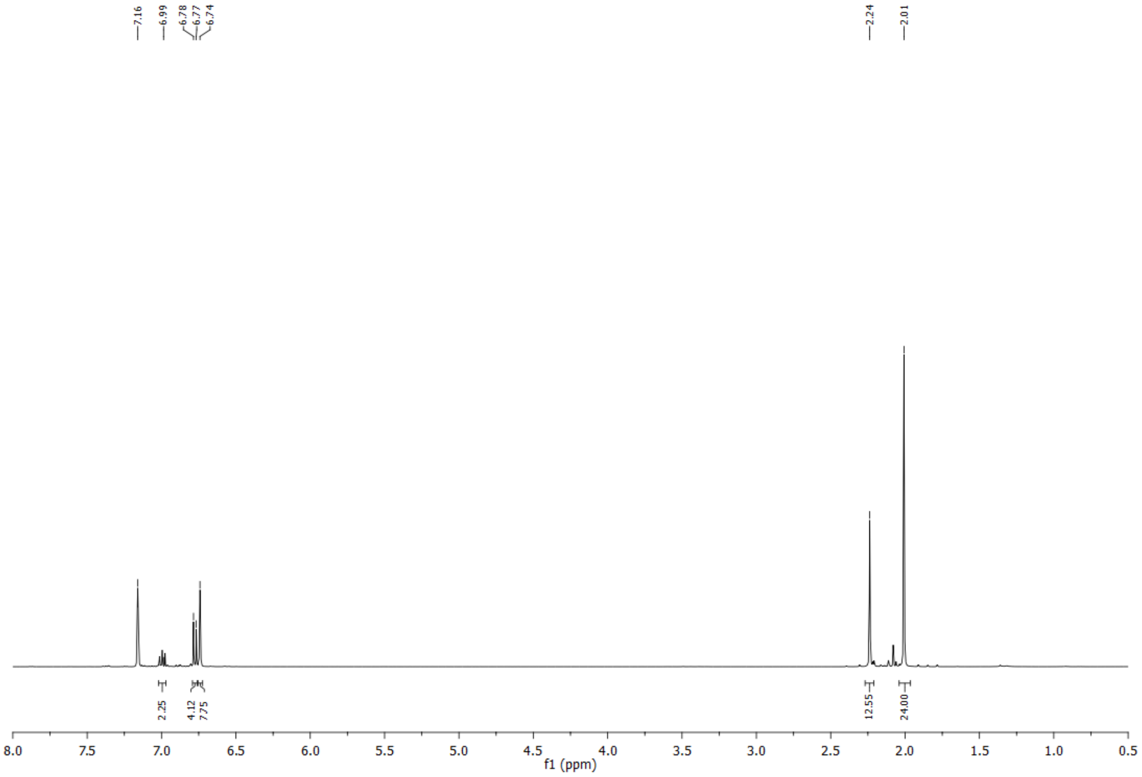

Figure S38. ^{1}H NMR spectrum of DMPTeTeDMP measured in benzene-d_6 at room temperature.

X. Quantum chemical calculations

All quantum chemical calculations for compounds **4-7** were performed by using the program package Orca 5.0.[4] The geometrical parameters were optimized by means of the density functional methods PBE0[5] with dispersion correction D4[6]. The basis sets def2-TZVPP[7], def2-QZVP[7] (for all atoms E>Ne) and the auxiliary basis set def2/J[8]. Frequency calculations were carried out for all optimized structures. Natural bond orbital analysis was performed using NBO version 7.0.[9]

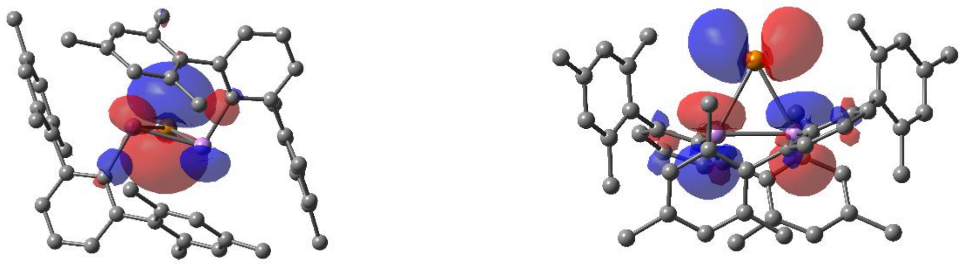

Figure S39. Calculated HOMO (left) and LUMO (right) of **4**.

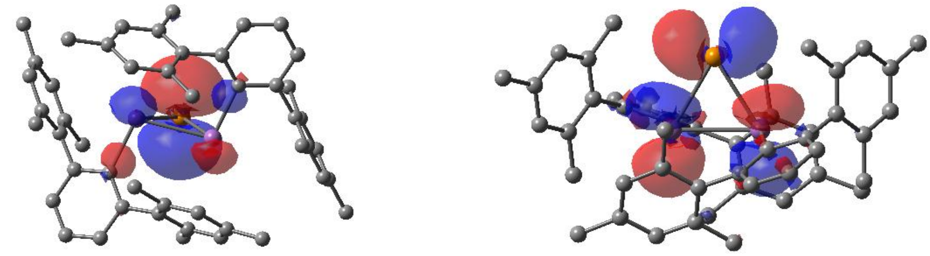

Figure S40. Calculated HOMO (left) and LUMO (right) of **5**.

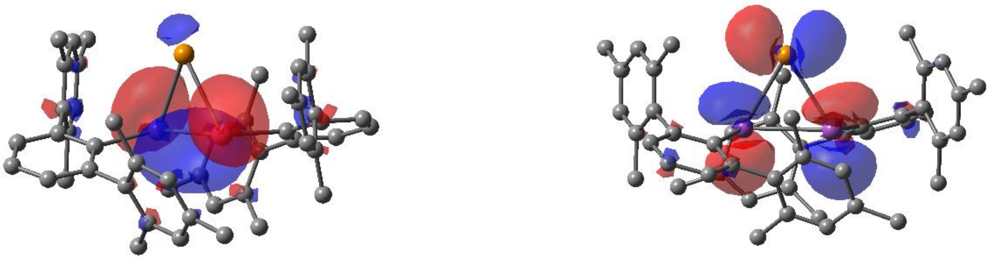

Figure S41. Calculated HOMO (left) and LUMO (right) of **6**.

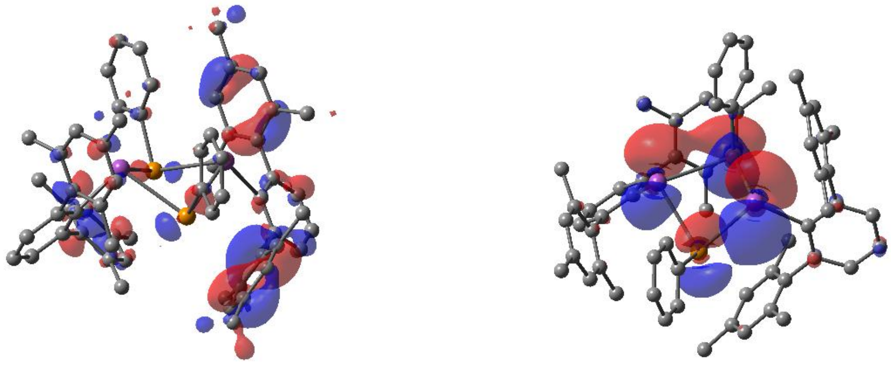

Figure S42. Calculated HOMO (left) and LUMO (right) of **7**.

Table S4. NPA atomic charges (q, e), occupation numbers (ON, |e|) of the bonds according to NBO, Wiberg bond index (WBI), Mayer bond order (MBO), bond polarization (P) and bond orbital character (OC) for the optimized geometry of **4**.

X-Y	q(X)	q(Y)	ON	WBI	MBO	P (X;Y)	OC (X;Y)
**Te-As1**	−0.13	0.39	1.95	0.95	0.95	55%; 43%	6% s, 94% p; 3% s, 96% p
**Te-As2**	−0.13	0.39	1.95	0.95	0.93	55%, 43%	6% s, 94% p; 3% s, 96% p
**As1-As2**	0.39	0.39	1.95	0.94	0.93	49%, 49%	8% s, 92% p; 7% s, 92% p

Table S5. NPA atomic charges (q, e), occupation numbers (ON, |e|) of the bonds according to NBO, Wiberg bond index (WBI), Mayer bond order (MBO), bond polarization (P) and bond orbital character (OC) for the optimized geometry of **5**.

X-Y	q(X)	q(Y)	ON	WBI	MBO	P (X;Y)	OC (X;Y)
**Te-Sb1**	−0.32	0.55	1.94	0.95	0.91	60%; 39%	6% s, 93% p; 3% s, 96% p
**Te-Sb2**	−0.32	0.56	1.94	0.95	0.88	60%; 39%	6% s, 93% p; 3% s, 96% p
**Sb1-Sb2**	0.55	0.56	1.96	0.92	0.96	49%; 49%	5% s, 94% p; 5% s, 94% p

Table S6. NPA atomic charges (q, e), occupation numbers (ON, |e|) of the bonds according to NBO, Wiberg bond index (WBI), Mayer bond order (MBO), bond polarization (P) and bond orbital character (OC) for the optimized geometry of **6**.

X-Y	q(X)	q(Y)	ON	WBI	MBO	P (X;Y)	OC (X;Y)
**Te-Bi1**	−0.40	0.62	1.94	0.92	0.93	62%; 37%	7% s, 93% p; 2% s, 97% p
**Te-Bi2**	−0.40	0.61	1.94	0.94	0.89	62%; 37%	6% s, 93% p; 2% s, 98% p
**Bi1-Bi2**	0.61	0.62	1.97	0.94	0.91	49%; 49%	3% s, 96% p; 3% s, 96% p

Table S7. NPA atomic charges (q, e), occupation numbers (ON, |e|) of the bonds according to NBO, Wiberg bond index (WBI), Mayer bond order (MBO), bond polarization (P) and bond orbital character (OC) for the optimized geometry of **7**.

X-Y	q(X)	q(Y)	ON	WBI	MBO	P (X;Y)	OC (X;Y)
**Te1-Bi1**	0.43	0.90	1.94	0.81	0.84	64%; 34%	6% s, 94% p; 2% s, 97% p
**Te1-Bi2**	0.43	0.87	1.95	0.73	0.74	68%; 29%	8% s, 92% p; 1% s, 98% p
**Te2-Bi1**	0.45	0.90	1.95	0.73	0.77	68%; 29%	7% s, 93% p; 1% s, 98% p
**Te2-Bi2**	0.45	0.87	1.95	0.82	0.85	65%; 35%	6% s, 93% p; 3% s, 97% p

Table S8. Cartesian coordinates (x,y,z) for the optimized geometry of **4**.

Te	21.546075	7.735857	0.570750
As	25.331059	8.083111	3.722446
As	21.071582	9.466276	5.166207
C	26.974206	11.208400	2.405539
C	19.638504	6.403252	6.833390
C	26.443205	13.726046	3.030591
C	28.975859	10.671985	0.738873
C	20.954002	4.457423	8.056987
C	16.979087	6.381760	6.833260
C	27.880337	15.652075	1.941068
C	24.448314	14.499012	4.853214
C	30.368479	12.630393	-0.326894
C	29.644681	8.005661	0.120021
C	19.595080	2.497364	9.186466
C	23.753359	4.367870	8.304083
C	15.676471	4.401240	7.969697
C	15.524737	8.493451	5.663973
H	27.461971	17.590294	2.443831
C	29.821417	15.122830	0.266135
C	22.194509	15.571936	3.966117
C	24.864304	14.241827	7.456270
H	31.895439	12.180927	-1.611794
C	28.716425	6.876011	-2.087458
C	31.201229	6.630737	1.767346
H	20.629650	1.006787	10.130826
C	16.978456	2.450908	9.138432
C	25.154738	2.644249	6.862345
C	24.974258	5.932092	10.059367
H	13.631076	4.413991	7.947561
C	14.515767	8.236935	3.231597
C	15.172210	10.747420	7.020576
H	30.912642	16.643894	-0.556875
C	20.398976	16.390709	5.705154
C	21.666675	15.793491	1.180173
C	23.018943	15.079903	9.131280
C	27.245089	13.052630	8.458154
C	29.256987	4.343188	-2.543609
C	27.184336	8.368110	-3.960923
C	31.711700	4.109650	1.233198
C	32.272638	7.834918	4.107494
H	15.960265	0.917073	10.028573
C	27.755701	2.491070	7.223260
C	23.908200	1.018360	4.889534
C	27.579004	5.730390	10.352088
C	23.521658	7.814898	11.619071
C	13.279391	10.289846	2.142269
C	14.716347	5.785330	1.804627
C	13.910981	12.742754	5.872302
C	16.174032	11.036544	9.664478
H	18.646839	17.191431	5.012901
C	20.783111	16.184003	8.293598

H	22.923089	17.153832	0.271546
H	19.727098	16.401601	0.857377
H	21.937310	13.988260	0.222334
H	23.349113	14.890321	11.144176
H	27.236338	11.005225	8.191124
H	27.445863	13.407968	10.475841
H	28.918950	13.771711	7.497041
H	28.511138	3.460745	-4.234779
C	30.724672	2.918805	-0.892724
H	28.384629	9.662334	-5.033021
H	25.747104	9.516276	-3.035352
H	26.249766	7.119397	-5.304621
H	32.909057	3.048490	2.512160
H	33.797814	6.692180	4.881111
H	30.829929	8.021292	5.572996
H	32.997887	9.724549	3.729248
H	28.834107	1.176050	6.084191
C	29.001389	3.999484	8.972690
H	22.703038	2.137982	3.647133
H	22.713383	-0.433927	5.738153
H	25.323784	0.077801	3.728508
H	28.517453	6.932940	11.719656
H	22.912483	9.433821	10.491624
H	24.685372	8.538895	13.155292
H	21.820953	6.980133	12.428132
H	12.535425	10.101150	0.243520
C	12.977207	12.563962	3.419529
H	14.306394	6.065292	-0.193590
H	13.372000	4.395641	2.531244
H	16.590023	4.948639	1.968906
H	13.656050	14.484828	6.919450
H	18.210176	11.381880	9.654824
H	15.849038	9.344688	10.791754
H	15.281807	12.631542	10.608716
C	18.878332	17.203118	10.140466
C	31.202593	0.157075	-1.369734
C	31.791722	3.716779	9.434488
C	11.651312	14.770520	2.211047
H	19.359188	19.134157	10.696272
H	18.805781	16.069692	11.859060
H	16.990188	17.251833	9.320392
H	31.057459	-0.298723	-3.373455
H	29.822427	-1.012557	-0.370943
H	33.073148	-0.409067	-0.715086
H	32.734317	2.839038	7.828290
H	32.142246	2.525305	11.085504
H	32.688904	5.537055	9.791979
H	11.146037	14.364269	0.256839
H	12.843378	16.455181	2.221109
H	9.917550	15.248272	3.225299

Table S9. Cartesian coordinates (x,y,z) for the optimized geometry of **5**.

Te	0.284801	11.594586	19.941257
Sb	2.147232	9.361102	24.362570
Sb	-3.155027	10.174838	23.690048
C	3.324548	12.765325	26.443195
C	-4.445175	6.528411	22.165427
C	5.945622	13.182158	26.329320
C	1.828164	14.385034	27.911193
C	-3.335708	4.145584	22.489710
C	-6.756797	6.712661	20.869149
C	7.033525	15.202456	27.617280
C	7.610691	11.449083	24.855530
C	2.963841	16.392295	29.195717
C	-0.952784	14.097807	28.233333
C	-4.533093	2.013225	21.495406
C	-0.921486	3.716205	23.871027
C	-7.907850	4.562512	19.883235
C	-8.010084	9.217984	20.552265
H	9.055520	15.491877	27.498063
C	5.544548	16.811223	29.051240
C	8.551123	9.248078	25.989977
C	8.214600	12.011677	22.334932
H	1.789746	17.626954	30.328575
C	-1.924831	12.280951	29.897754
C	-2.598243	15.741715	26.959544
H	-3.665642	0.179705	21.764364
C	-6.797505	2.209921	20.195489
C	-0.872835	3.717216	26.518676
C	1.293290	3.194777	22.506758
H	-9.683047	4.746645	18.882669
C	-9.602839	10.148662	22.455463
C	-7.565545	10.653705	18.368744
H	6.390321	18.375660	30.061081
C	10.032704	7.608712	24.561787
C	7.921933	8.609989	28.688298
C	9.684933	10.315833	20.970205
C	7.278284	14.403421	21.118680
C	-4.525972	12.149329	30.271822
C	-0.212754	10.453800	31.242332
C	-5.185603	15.562484	27.404260
C	-1.608853	17.634714	25.081655
H	-7.696617	0.535532	19.440160
C	1.386702	3.184352	27.758370
C	-3.197419	4.329908	28.035526
C	3.506432	2.645795	23.818422
C	1.320647	3.296616	19.664400
C	-10.655950	12.542319	22.180426
C	-10.135149	8.624210	24.796105

C	-8.641800	13.043903	18.176141
C	-5.950182	9.636305	16.263863
H	10.753142	5.904982	25.440307
C	10.606312	8.098213	22.046325
H	5.953435	8.009465	28.874419
H	8.166827	10.232389	29.932659
H	9.106565	7.073416	29.373119
H	10.121062	10.736036	19.013417
H	7.781177	14.457876	19.123710
H	8.084769	16.069758	22.030041
H	5.229803	14.574908	21.270106
H	-5.270493	10.736617	31.554731
C	-6.187487	13.791910	29.064368
H	0.562026	9.052795	29.935895
H	-1.236787	9.432107	32.706867
H	1.392944	11.409907	32.109250
H	-6.450823	16.815386	26.393831
H	-0.370194	16.730764	23.703812
H	-0.510850	19.119595	26.000152
H	-3.160140	18.523537	24.062355
H	1.415968	3.189416	29.806613
C	3.591495	2.615749	26.443145
H	-3.585450	6.360217	28.018606
H	-2.959391	3.766342	30.001202
H	-4.869888	3.394067	27.282664
H	5.216579	2.272676	22.756415
H	0.125329	1.835923	18.834698
H	3.233274	3.042789	18.948484
H	0.615852	5.110660	18.981021
H	-11.886593	13.258869	23.652329
C	-10.172276	14.033353	20.071889
H	-10.621622	6.675062	24.342486
H	-11.682648	9.453217	25.868856
H	-8.484776	8.551963	26.037886
H	-8.274159	14.161651	16.499736
H	-6.835370	8.002408	15.365666
H	-4.111793	9.013967	16.956297
H	-5.638629	11.069095	14.819459
C	12.226227	6.316099	20.533920
C	-8.981797	13.685424	29.570556
C	5.985870	1.939227	27.816366
C	-11.245841	16.658098	19.861977
H	11.426495	5.979257	18.663295
H	12.428607	4.498011	21.480123
H	14.121084	7.089844	20.255837
H	-10.063775	14.183403	27.890545
H	-9.510302	15.013976	31.061998
H	-9.567395	11.807702	30.181075

H	7.644304	2.619585	26.802487
H	6.172841	-0.109903	28.002717
H	6.018219	2.737319	29.714497
H	-11.673904	17.143488	17.905571
H	-9.900892	18.062067	20.561574
H	-12.973763	16.854755	20.966489

Table S10. Cartesian coordinates (x,y,z) for the optimized geometry of **6**.

Bi	-3.447343	26.333569	23.423922
Bi	2.128744	27.278610	24.112989
Te	0.156608	24.919439	19.611440
C	-4.800464	30.175302	21.863504
C	3.335898	23.738653	26.359138
C	-3.653087	32.541445	22.154142
C	-7.150604	30.022510	20.640548
C	1.806089	22.091803	27.756586
C	5.962056	23.362769	26.354858
C	-4.858023	34.694813	21.212384
C	-1.173507	32.932224	23.430588
C	-8.316087	32.189529	19.706889
C	-8.420941	27.523623	20.341881
C	2.914379	20.091077	29.077216
C	-0.989532	22.349474	27.977372
C	7.028967	21.351062	27.676196
C	7.655567	25.149165	24.980138
H	-3.958378	36.517319	21.452229
C	-7.170800	34.529163	19.994732
C	-1.014735	32.993861	26.075228
C	0.995595	33.363133	21.967005
H	-10.124421	32.030601	18.762078
C	-10.017827	26.612994	22.250141
C	-7.962151	26.059110	18.179666
H	1.713852	18.832762	30.155846
C	5.505107	19.709663	29.035443
C	-2.038363	24.123553	29.643395
C	-2.574973	20.713694	26.618924
H	9.058906	21.093340	27.643914
C	8.534560	27.326250	26.205146
C	8.339669	24.670842	22.461713
H	-8.076878	36.216853	19.278168
C	1.304772	33.499583	27.209467
C	-3.288958	32.463806	27.694016
C	3.275670	33.890882	23.175510
C	0.898635	33.200834	19.129541
C	-11.072993	24.216402	21.997652
C	-10.562966	28.168904	24.568794
C	-9.040828	23.668308	18.008537

C	-6.314360	27.042946	16.084680
H	6.331227	18.150384	30.069345
C	-4.652535	24.224086	29.930931
C	-0.392116	25.942002	31.077121
C	-5.179702	20.860469	26.977558
C	-1.500010	18.860588	24.749393
C	10.048959	29.019827	24.875973
C	7.828080	27.872387	28.906474
C	9.840231	26.417620	21.197536
C	7.450942	22.316534	21.140643
H	1.417467	33.539576	29.254710
C	3.469432	33.982221	25.792404
H	-4.965274	33.434894	26.996646
H	-2.955156	33.039579	29.642039
H	-3.729387	30.443507	27.711035
H	4.947205	34.205389	22.036080
H	0.111553	31.394352	18.521355
H	2.783928	33.382654	18.324737
H	-0.290962	34.678763	18.322327
H	-12.310581	23.517789	23.471988
C	-10.582907	22.702220	19.907203
H	-11.224086	30.057525	24.077084
H	-8.872608	28.424970	25.728213
H	-11.993131	27.260836	25.736180
H	-8.663191	22.530125	16.348250
H	-6.087368	25.631097	14.604571
H	-4.440200	27.548634	16.778862
H	-7.117738	28.743555	15.236445
H	-5.454646	25.604094	31.214668
C	-6.256500	22.591845	28.633483
H	0.418796	27.363847	29.814854
H	1.188397	24.984373	31.987183
H	-1.476737	26.935297	32.517274
H	-6.396735	19.611405	25.905280
H	-3.004850	17.933580	23.695121
H	-0.376241	17.404699	25.682011
H	-0.262717	19.810521	23.401271
H	10.731071	30.697390	25.830794
C	10.706872	28.610491	22.367464
H	8.913586	29.459693	29.639001
H	8.152790	26.241835	30.122295
H	5.824575	28.344139	29.084390
H	10.348653	26.056053	19.246529
H	5.392306	22.194139	21.125241
H	8.141168	20.620173	22.089715
H	8.108196	22.277090	19.190883
C	5.932649	34.617800	27.058550
C	-11.658888	20.076758	19.718040

C	-9.068760	22.675288	29.032037
C	12.314349	30.475468	20.944262
H	7.527247	34.246539	25.811390
H	6.196908	33.521742	28.782710
H	5.997349	36.614894	27.583472
H	-12.150193	19.601966	17.773719
H	-13.349629	19.871041	20.876825
H	-10.289503	18.670399	20.363154
H	-9.698909	24.561478	29.568279
H	-9.638339	21.381515	30.538798
H	-10.077643	22.120364	27.325118
H	13.938130	29.551049	20.069947
H	13.021351	31.959120	22.185385
H	11.242412	31.379110	19.428504

Table S11. Cartesian coordinates (x,y,z) for the optimized geometry of **7**.

Bi	11.146668	12.477780	10.520701
Te	5.678086	12.481823	9.341792
Te	10.444879	7.000052	9.221278
C	12.711262	14.127845	6.941226
Bi	5.772257	7.541952	12.209625
C	4.625469	14.788427	12.454533
C	12.955296	5.794508	12.111551
C	13.423846	12.929550	4.702235
C	13.114258	16.717004	7.319642
C	3.272920	5.054432	9.844910
C	2.867249	16.684770	11.988675
C	5.633426	14.452943	14.859674
C	12.685657	6.511297	14.626135
C	14.965822	4.282357	11.351824
C	14.568215	14.402590	2.835340
C	12.989974	10.200178	4.191390
C	14.254459	18.133700	5.425427
C	12.301569	17.852684	9.770166
C	1.360879	5.780841	8.179429
C	3.660157	2.521910	10.502435
H	2.082150	16.946003	10.122247
C	2.108878	18.251947	13.956578
C	4.861952	16.024264	16.813910
H	7.015843	12.995357	15.238230
H	11.116229	7.670010	15.236399
C	14.452947	5.707662	16.390964
C	16.729707	3.491525	13.132432
H	15.173194	3.723857	9.397133

H	15.135535	13.511788	1.084215
C	14.982164	16.968948	3.188761
C	10.742388	9.443837	3.008796
C	14.853268	8.413832	4.786993
H	14.573645	20.133744	5.705067
C	9.856899	18.875895	10.018519
C	13.977698	17.866781	11.849106
C	-0.159753	3.876115	7.161727
C	0.857571	8.446188	7.451069
C	2.124365	0.672318	9.442954
C	5.675904	1.870115	12.360038

XI. *In situ* NMR study of the formation of compound 7

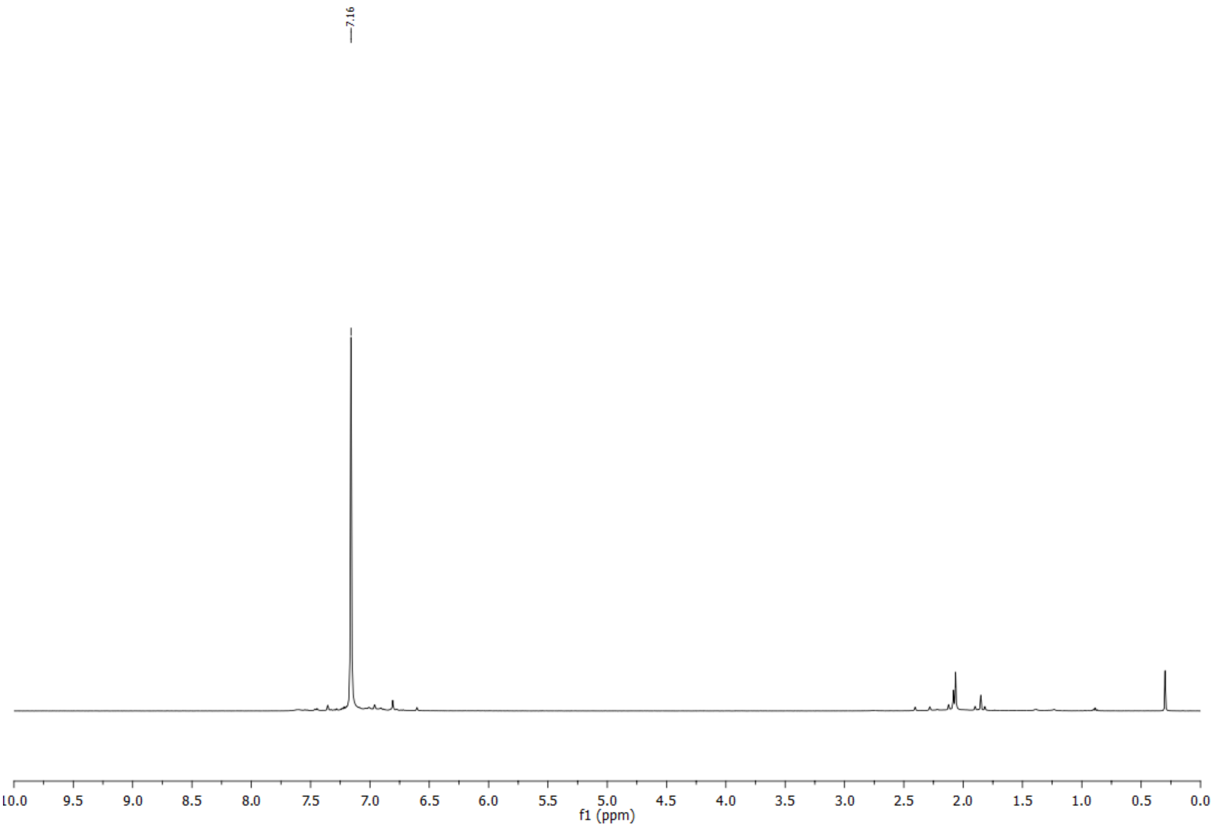

Figure S43. *In situ* ^{1}H NMR spectrum of the reaction of compound **3** with $AgSbF_6$ in benzene-d_6 measured at room temperature. After addition of $AgSbF_6$ a mixture of a yellow and red precipitate formed. As shown here, the resulting supernatant features almost no resonances except from solvent residues.

XI. References

1 G. M. Sheldrick, *Acta Crystallogr.*, 1990, **A46**, 467–473.

2 G. M. Sheldrick, SHELXL-2017, Program for the Refinement of Crystal Structures University of Göttingen, 2017. See also: G. M. Sheldrick, *Acta Cryst.*, *2015*, **C71**, 3–8.

3 C. B. Hübschle, G. M. Sheldrick and B. Dittrich, *J. Appl. Cryst.*, 2011, **44**, 1281–1284.

4 F. Neese, F. Wennmohs, U. Becker and C. Riplinger, *J. Chem. Phys.,* 2020, **152**, 224108.

5 C. Adamo and V. Barone, *J. Chem. Phys.,* 1999, **110**, 6158–6170

6 E. Caldeweyher, J.-M. Mewes, S. Ehlert and S. Grimme, *Phys. Chem. Chem. Phys.,* 2020, **22**, 8499–8512.

7 F. Weigend and R. Ahlrichs, *Phys. Chem. Chem. Phys.,* 2005, **7**, 3297–3305.

8 F. Weigend, *Phys. Chem. Chem. Phys.,* 2006, **8**, 1057–1065.

9 E. D. Glendening, J. K. Badenhoop, A. E. Reed, J. E. Carpenter, J. A. Bohmann, C. M. Morales, P. Karafiloglou, C. R. Landis and F. Weinhold, *NBO 7.0, Theoretical Chemistry Institute*, University of Wisconsin, Madison, WI, 2018.
